# An integrated taxonomic approach refines the *Imperator torosus* species concept and reveals the cryptic *I. occultus* sp. nov. (*Boletales*, *Boletaceae*)

**DOI:** 10.3897/mycokeys.142.208955

**Published:** 2026-11-02

**Authors:** Matteo Gelardi, Neven Ferenčak, Alona Yu. Biketova, Marko Požnjak, Artemis Diana Treindl, Andrin Gross, Günter Saar, Marco Cartabia, Jacques Guinberteau, László Albert, Michal Mikšík, Eva Zupan, Pierre-Arthur Moreau, Jean-Michel Bellanger, Boris Assyov, Bálint Dima, Alfredo Vizzini

**Affiliations:** 1 Jodrell Laboratory, Royal Botanic Gardens, Kew, Richmond TW9 3DS, UK Jodrell Laboratory, Royal Botanic Gardens, Kew Richmond United Kingdom; 2 Via dei Barattoli 3A, I-00061 Anguillara Sabazia (RM), Italy Unaffiliated Anguillara Sabazia Italy; 3 Vladimira Nazora 12, 42000 Varaždin, Croatia Unaffiliated Varaždin Croatia; 4 Institute of Evolution and Department of Evolutionary & Environmental Biology, University of Haifa, Aba Khoushi Ave. 199, Mt. Carmel, Haifa 3498838, Israel Institute of Evolution and Department of Evolutionary & Environmental Biology, University of Haifa Haifa Israel; 5 Dragojla Kušlana 34A, 10000 Zagreb, Croatia Unaffiliated Zagreb Croatia; 6 ETH Zurich, Mycological Collection Z+ZT, Universitätsstrasse 16, Zurich, Switzerland ETH Zurich, Mycological Collection Z+ZT Zurich Switzerland; 7 Swiss Federal Institute for Forest, Snow and Landscape Research WSL, Zürcherstrasse 111, Birmensdorf, Switzerland Swiss Federal Institute for Forest, Snow and Landscape Research WSL Birmensdorf Switzerland; 8 Dammenmühle 7, 77933 Lahr, Germany Unaffiliated Lahr Germany; 9 Via Lazio 19, 21100 Varese, Italy Unaffiliated Varese Italy; 10 No 9 rue Pierre Chaumaure, F-05200 Puy-Sanieres, France Unaffiliated Puy-Sanieres France; 11 Hungarian Mycological Society, Pázmány Péter sétány 1/c, H-1117, Budapest, Hungary Hungarian Mycological Society Budapest Hungary; 12 Pobřežní 40, Prague 8, 18600, Czech Republic Unaffiliated Prague Czech Republic; 13 Šalovci 22, 2277 Središče ob Dravi, Slovenia Unaffiliated Središče ob Dravi Slovenia; 14 UFR3S Département de Pharmacie, Université de Lille, ULR 4515 – LGCgE, Laboratoire de Génie Civil et géo-Environnement, 3 rue du Pr Laguesse, 59000 Lille, France UFR3S Département de Pharmacie, Université de Lille, ULR 4515 – LGCgE, Laboratoire de Génie Civil et géo-Environnement Lille France; 15 CEFE, Univ Montpellier, CNRS, EPHE, IRD, INSERM, Montpellier, France CEFE, Univ Montpellier, CNRS, EPHE, IRD, INSERM Montpellier France; 16 Institute of Biodiversity and Ecosystem Research, Bulgarian Academy of Sciences, 2 Gagarin Str., 1113 Sofia, Bulgaria Institute of Biodiversity and Ecosystem Research, Bulgarian Academy of Sciences Sofia Bulgaria; 17 Department of Plant Anatomy, Institute of Biology, Eötvös Loránd University, Pázmány Péter sétány 1/c, H-1117, Budapest, Hungary Department of Plant Anatomy, Institute of Biology, Eötvös Loránd University Budapest Hungary; 18 Department of Life Sciences and Systems Biology, University of Turin, Viale P.A. Mattioli 25, I-10125, Turin, Italy Department of Life Sciences and Systems Biology, University of Turin Turin Italy

**Keywords:** *
Agaricomycetes
*, European boletes, macrofungi, molecular phylogeny, new species

## Abstract

*Imperator torosus* is a polymorphic, critical Friesian bolete, originally described from Central Europe, but with a convoluted taxonomic history which caused much uncertainty in delimiting it from similar-looking species. Previous molecular phylogenetic analyses confidently indicated that *I. torosus*, as presently circumscribed, is a polyphyletic species in need of taxonomic revision. Based on a two-locus (ITS and LSU) phylogeny, pairwise distance and percent nucleotide similarity analyses, as well as morphological and ecological evidence, *I. torosus* s. str. is defined and *Imperator occultus* described as a novel species. Besides unravelling the intricate taxonomic history of *I. torosus* s. l., detailed macro- and micro-morphological descriptions based on well-documented collections are provided for both *I. torosus* s. str. and *I. occultus*, accompanied by colour photographs taken in their natural habitats and line drawings of their main microscopic features. A comprehensive and circumstantial discussion upon the reasons why the recent epitypification of *B. torosus* is deemed untenable is presented. Morphological comparisons with xanthoid phenotypes of *Imperator rhodopurpureus* (*I. rhodopurpureus* f. xanthopurpureus and *I. xanthocyaneus*), as well as their taxonomic placement within the genus *Imperator* are also elucidated and discussed.

## Introduction

*Boletus torosus* Fr. was named by Fries in Fries and Hök (1835) based on a description by Secretan (1833) under the misapplied name *Boletus pachypus* Fr. (Fries 1815). The Swiss mycologist observed this species in the beech forest of Sauvabelin (“coteau oriental”), located on the northern shore of Lake Geneva, not far from Lausanne, canton of Vaud, Switzerland (Secretan 1833). Since its initial description, the taxonomic concept of *B. torosus* was prone to misinterpretation because Fries’ diagnosis (Fries and Hök 1835) was very short and elusive, and only based on Secretan’s description (Fries 1838). Moreover, the absence of supporting holotype material (no specimen or illustrations available), the surprising chromatic variability of the basidiomes, together with the infrequent occurrence of this species in nature led to divergent interpretations over time.

It was not until Quélet (1886, 1888) that *B. torosus* was described in more detail and in a non-compilatory manner, based on fresh collections made in Fontainebleau and southern Jura (France). To the same period should be dated the first known coloured illustration of *B. torosus* by the French botanist and entomologist Jean-Henri Fabre in 1890 under the name *B. pachypus*, although with some obvious incongruences (see Mikšík et al. 2025). Quélet’s concept of *B. torosus* was later followed by Barbier (1904), Migula (1912), Sartory and Maire (1931), Gilbert (1936) and Konrad and Maublanc (1936), although Konrad (1932) downgraded *B. torosus* to the rank of subspecies of *B. appendiculatus* Schaeff. Nonetheless, another French mycologist, Peltereau (1926), published a coloured illustration named “*B. regius* Krombh. forme (*Boletus torosus*?)”, in fact a faintly depigmented phenotype of a red-pored bolete currently known as *Rubroboletus legaliae* f. *spinarii* (Hlaváček) Mikšík. Prior to the aforementioned authors, the Swiss mycologist Knapp (1924a, 1924b) offered a detailed and “modern” description of *B. torosus*, especially with respect to the pileal tint, which was reported as follows: “pale greenish, gray-yellow-greenish, pale gray-yellow, also yellow in places, or almost completely yellow like *Boletus elegans*, but later pale brownish, leather or dead leaf brownish, somewhat leather brown and red-spotted, blue-red, red or blue black when handled” (“grünlichblass, grünlichgraugelb, fahlgraugelb, auch stellenweise gelb, oder fast ganz gelb wie *Bol. elegans*, später aber blassbräunlich, leder- oder laubbräunlich- fast lederbraun und rotfleckig, bei Druck blau-rot, rot oder blauschwarz”) (Knapp 1924a). This author reassessed the same species some eleven years later (Knapp 1935) and his interpretation was then reinforced by Kern (1946). Around the same period, Hans Walty’s Schweizer Pilztafeln für den praktischen Pilzsammler, II. Band, issued by the Verband Schweizerischer Vereine für Pilzkunde (Walty 1944 [1^st^ edn], 1947 [2^nd^ edn]; later reissued in 1957, 1965 and 1979), provided a short but concise description of *B. torosus*, accompanied by the first meaningful coloured illustration of this species (based on three original 1927 Walty watercolours – A-273, A-274 and A-275 (Walty 1927a, 1927b, 1927c) – preserved in the Swiss National Library, Prints & Drawings Dept.). Plate A-274 was published in Mangold (2025). These plates were reproduced later by Singer (1967) and subsequently by Pilát and Dermek (1974) and Dermek and Pilát (1988), who contributed to shaping the concept of this species in its modern sense.

In the same period, however, two morphologically similar taxa were introduced. One was described from the former Czechoslovakia by Smotlacha (1952) as a simple xanthoid phenotype of the newly published *B. rhodopurpureus* Smotl. (*B. rhodopurpureus* f. xanthopurpureus Smotl.). The other was presented, albeit invalidly (Art. 39.1, Latin diagnosis absent), by Ramain (1948) as *B. purpureus* subsp. *xanthocyaneus* Ramain, based on material collected at various sites on the southern shore of Lake Geneva, near Douvaine (forest of Douvaine-Loisin), lower Chablais, Haute-Savoie, France. Probably due to their resemblance and geographic proximity, in the following decades *B. purpureus* subsp. *xanthocyaneus* was largely ignored or almost unanimously considered a heterotypic synonym of *B. torosus*, resulting in the related descriptions being generally mixed up (Imler 1950; Konrad and Maublanc 1952; Kühner and Romagnesi 1953; Heim 1957; Michael and Hennig 1958; Hotz 1963; Piane 1966; Singer 1967; Leclair and Essette 1969; Burkhard 1971; Balletto 1972; Pilát and Dermek 1974; Galli 1980; Alessio 1985, 1993; Angarano 1989; Breitenbach and Kränzlin 1989). Romagnesi (1976) was the first to cast doubts about the conspecificity of *B. torosus* and *B. purpureus* subsp. *xanthocyaneus*. The French author validated Ramain’s taxon, designating a new collection from the north of Paris as holotype, and eventually raised it to species level as *B. xanthocyaneus* Ramain ex Romagn.

The presence of morphologically similar taxa contributed to confusion in the literature. For example, Dermek and Lizoň (1980), Engel et al. (1983) and Alessio (1985) produced novel colour plates of *B. torosus*, all being ambiguous. Several other authors misinterpreted this taxon, describing different species (Table 1: A). On the other hand, Cetto (1970, 1983, 1987, the latter two both as *B. torosus* var. *xanthus* Cetto nom. inval.), Angarano (1989, as “*B. torosus* (var. *xanthus*? Fr.?)” nom. inval.), Ricek (1989) and Foiera et al. (1993) provided good illustrations or colour photographs of *B. torosus*, which largely fit the modern definition of this species.

**Table 1.** Column A lists misapplications of the epithet *torosus* to species other than *I. torosus* s. str. and *I. occultus* (citations are followed by the country of origin in brackets). Column B covers the broad concept of *I. torosus* in modern literature, encompassing *I. torosus* s. str. and *I. occultus*. Sources in column B may concern either species or both. Column C lists records or illustrations likely to represent *I. occultus*. A publication may appear in more than one column, and an entry may refer to only part of that publication. Inclusion in Column B alone does not establish identification as *I. torosus* s. str. Literature interpretations are based on morphological descriptions and/or illustrations. All bibliographic citations are arranged chronologically.

**A. Misapplications of the name *I. torosus* s. l. in modern literature**		**B. Modern literature treating *B. torosus* in the broad sense**	**C. Records of *I. torosus* s. l. likely to be referred to *I. occultus***
**Literature citations and country of origin**:	**Current identity**:		
Blum 1962, 1968 (France)	Mixed species	Merlet 1997; Piltaver 1997, 2009; Galli 1998, 2007, 2013, 2023; Peintner et al. 1999; Krieglsteiner 2000; Cazzoli and Consiglio 2001; Lannoy and Estadès 2001; Waldvogel et al. 2001; Klofac 2003, 2007, 2011; Estadès and Lannoy 2004; Vázquez Pardo et al. 2004; Röger 2004, 2007, 2009; Horak 2005; Moingeon and Moingeon 2005, 2007; Muñoz 2005; Redeuilh and Alpago Novello 2005; Alpago Novello 2006; Benedek and Pál-Fám 2006; Gröger 2006; Courtecuisse and Duhem 2007; Fernández-Vicente and Undagoitia 2007; Boccardo et al. 2008; Flammer 2008; Gelardi 2008b; Martinelli and Senn-Irlet 2008; Riva 2008; Schreiner 2008; Surault 2008; Albizu and Teres 2009; Consiglio and Papetti 2009; Padovan 2009; Šutara et al. 2009*pro parte*; Noordeloos 2010*pro parte* (figs 6, 7); Albert 2011; Kibby 2011, 2017; Maletti 2011; Boutard and Hervé 2012; Chéreau et al. 2012; Simonini and Bertolini 2012; Reichert and Reil 2012; Zamotajlov et al. 2012; Bertolini and Simonini 2013; Giolo 2013; Reumaux and Carteret 2013; Šikić 2013; Vasquez 2014; Hagara 2015; Hahn 2015; Mikšík 2015, 2017*pro parte*; Kunca 2016; Dämon and Krisai-Greilhuber 2017; Todeschini et al. 2017; Noordeloos et al. 2018; Raya López and Moreno Arroyo 2018; Ferrarese 2019; Klofac and Krisai-Greilhuber 2020; Oppicelli 2020, 2025; Padovan et al. 2020; Page et al. 2021; Illice et al. 2023; Winkler and Keller 2023; Gasparini 2024; Gürzoğlu et al. 2024; Mikšík and Polčák 2024; Wald 2024; Konstantinidis 2025; Mikšík et al. 2025; Mancari 2026	Raris 1970a; Dermek 1979; Gallinari et al. 1983; Galli 1987, 1996, 1998, 2007, 2013, 2023; Fourré 1990, 2003a*pro parte*, 2003b *pro parte*, 2004 *pro parte*, 2006; Redeuilh 1992b, 2002; Brillouet and Brillouet 1993; Rimóczi 1994; Simonini 1995, 1996; Merlet 1997; Venturella et al. 1999; Cazzoli and Consiglio 2001; Redeuilh et al. 2004; Moingeon and Moingeon 2005, 2007; Muñoz 2005; Redeuilh and Alpago Novello 2005; Benedek and Pál-Fám 2006; Karadelev et al. 2007, 2018; Courtecuisse and Duhem 2007; Fernández-Vicente and Undagoitia 2007; Boccardo et al. 2008; Flammer 2008; Martinelli and Senn-Irlet 2008; Riva 2008; Surault 2008; Albizu and Teres 2009; Consiglio and Papetti 2009; Šutara et al. 2009*pro parte*; Maletti 2011; Wuilbaut 2011; Boutard and Hervé 2012; Lacombe 2012; Lannoy 2012; Rodà 2012; Simonini and Bertolini 2012; Reumaux and Carteret 2013; Šikić 2013; Vasquez 2014; You 2016; Mikšík 2017*pro parte*; Todeschini et al. 2017; Mazza 2018; Siniscalco et al. 2019a; Oppicelli 2020, 2025; Padovan et al. 2020; Sarkina 2020b; Illice et al. 2023; Mikšík and Polčák 2024, as “*Imperator* sp.”; Mikšík et al. 2025, as “*Imperator cf. torosus*”; Benigni et al. 2026
Avizohar-Hershenzon and Binyamini 1972 (Israel)	*Suillellus comptus* (Simonini) Vizzini, Simonini & Gelardi		
Marchand 1973 (France)	Mixed species		
Binyamini 1975 (Israel)	*S. comptus*		
Merlo et al. 1980 (Italy)	*Imperator rhodopurpureus* f. xanthopurpureus (Smotl.) Mikšík		
Galli 1982 (Italy)	*Imperator luteocupreus* (Bertéa & Estadès) Assyov et al.		
Hagara 1982 (Slovakia)	Mixed species		
Rocabruna 1985 (Spain)	*I. luteocupreus*		
Moser and Jülich 1986 (Central Europe)	*Imperator rhodopurpureus* (Smotl.) Assyov et al.		
Vaasma et al. 1986 (south-western Russia)	Mixed species		
Focht 1987 (Croatia)	*I. rhodopurpureus*		
Angarano 1989 (Italy)	Mixed species		
Breitenbach and Kränzlin 1989, 1991 (Switzerland)	*I. luteocupreus*		
Kluzák et al. 1990 (Czechia)	*I. rhodopurpureus* f. xanthopurpureus		
Brotzu 1993 (Italy, Sardinia)	*Cyanoboletus poikilochromus* (Pöder, Cetto & Zuccher.) M. Carbone, D. Puddu & P. Alvarado		
Cetto 1993 (as “*Boletus torosus* Fr. f.ma”) (Italy)	*I. rhodopurpureus* var. *polypurpureus* (Smotl.) Mikšík		
Hlaváček 1996, 1997 (Czechia)	*I. rhodopurpureus* f. xanthopurpureus		
Hills 1997, 2007 (UK)	*I. rhodopurpureus* f. xanthopurpureus		
Ruiz Fernández 1997 (Spain)	*I. rhodopurpureus* and *I. rhodopurpureus* f. xanthopurpureus		
Hagara et al. 1999 (Czechia)	*Rubroboletus rhodoxanthus* (Krombh.) Kuan Zhao & Zhu L. Yang		
Gerhardt et al. 2000 (Spain)	*I. rhodopurpureus*		
Papoušek 2004 (Czechia)	*I. rhodopurpureus* f. xanthopurpureus		
Watling and Hills 2005	*I. rhodopurpureus* f. xanthopurpureus		
Ruiz Fernández and Ruiz Pastor 2006 (Spain)	*I. rhodopurpureus*		
Calzada Domínguez 2007 (Spain)	? *Neoboletus xanthopus* (Klofac & A. Urb.) Klofac & A. Urb.		
Egri 2008, 2009 (Hungary)	*I. luteocupreus*		
Brotzu and Colomo 2009 (Italy, Sardinia)	*I. luteocupreus* and *C. poikilochromus*		
Noordeloos 2010 (Netherlands)	*I. rhodopurpureus*		
Bellù and Veroi 2014 (Italy)	*I. luteocupreus*		
Veerkamp and Dam 2019 (Netherlands)	*I. rhodopurpureus*		

In recent times, Redeuilh (1992a, 1992b) was one of the first boletologists to provide a comprehensive historical overview accompanied by a very detailed description of *B. torosus* based on abundant fresh collections yielded by G. Redeuilh himself, R.-C. Azéma and A. Estadès in the forest of Chizé (Deux-Sèvres), Jura and Isère. In his papers, *B. torosus* is described as a fleshy and massive, dense, compact, particularly heavy species with a pileus showing three main colours: yellow, grayish and greenish, with one tint usually prevailing over the other two (especially yellow in the early developmental stage) and often with scattered reddish vinaceous to purple brown or dull olivaceous brown spots at maturity, tending to spread gradually and becoming predominant and uniform in aged specimens; pores long-lasting bright yellow but later taking a faint reddish hue; a robust stipe mostly yellow but reddish at the base and ornamented by a well-developed concolourous reticulum; yellow context; tissues immediately and intensely bluing on handling or exposure then turning reddish; ellipsoid-fusiform, smooth basidiospores, a trichodermal pileipellis and the occurrence under broadleaved trees of the family *Fagaceae* (*Castanea*, *Fagus*, *Quercus*) or in mixed stands with conifers on basic-calcareous soil, in summer and early autumn (Redeuilh 1992b). A purported conspecificity of *B. torosus* and *B. xanthocyaneus* was maintained doubtful and Redeuilh also sustained the hypothesis of a possible close affinity of the latter species with *B. rhodopurpureus* f. xanthopurpureus Smotl. (Redeuilh 1992b). *Boletus torosus* and related species were also grouped within the informal “stirps *Inquinans*” (Redeuilh 1992b). Previously, Bertéa and Estadès (1990) and Cazzoli (1991) had already assigned *B. torosus* to the informal groupings “group *Torosus*” and “stirps *Torosus*”, respectively, together with closely allied species characterized by a rapid and strong blueing upon handling. Similarly, the “series *Torosi*”, as defined many years later by Lannoy and Estadès (2001) as well as *Boletus* (subg. *Suillellus*) sect. *Purpurei* Watling & A.E. Hills (Watling and Hills 2005) were both invalidly published.

Brillouet and Brillouet (1993), based on previous informative publications (Brillouet 1987; Brillouet et al. 1989), provided further insights pertaining to the reciprocal separation of *B. torosus* from *B. xanthocyaneus*. They essentially retained Redeuilh’s interpretation, emphasizing the macro-morphological features reported by the French author as the most useful field characters for identification, but also highlighting the importance of different ecological requirements, *B. xanthocyaneus* showing a strong preference for acidic soils. Simonini (1995, 1998) also adopted this concept of *B. torosus*, and he integrated their observations by outlining the diagnostic value of a significant organoleptic trait, i. e. a pungent odour of expired medicines, pharmaceutical lab, preservatives or recalling that of *Bromelia ananas* fruit (see also Singer 1967). Curiously, this unique scent was previously specified in the protologue by Secretan – “L’odeur est très-pénétrante” (“The smell is very strong”; Secretan 1833) and repeated in the original diagnosis by Fries – “Odor fortis” (“Strong smell”; Fries and Hök 1835) but was overlooked by most of the authors. In addition, Simonini (1995, 1996, 1998) highlighted the remarkable sordid purple-olivaceous-brown colour of the pileus in aged basidiomes as matching the definitions of “basane livide” by Secretan (1833) and “fuscescente” by Fries and Hök (1835). In the second half of the 1990s, Hlaváček (1996, 1997) assigned *B. torosus* and allies to the formally recognised *Boletus* sect. *Luridi* subsect. *Torosi* Hlaváček, with the same *B. torosus* as the type species, whereas Fourré (1998a, 1998b), in two different papers, addressed the problem of the edibility of this bolete.

Shortly after, Estadès and Deïana (2004) provided the most detailed and comprehensive macroscopic description of *B. torosus* known to date, based on more than one hundred specimens yielded at various localities in France (départements of Ain, Isère, Jura, Savoie) and critically re-examined morphological and edaphic traits discriminating *B. torosus* and *B. xanthocyaneus* from each other. Based on the available knowledge at that time, the taxonomy of *B. torosus* was then stabilized by a consolidated tradition and contemporaneous and subsequent descriptions and/or illustrations mostly showed remarkable consistency in the species concept (Table 1: B) with only a few relevant exceptions of misidentification with other similar taxa (Table 1: A). Notably, British records of *B. torosus* (Hills 1997, 2007; Watling and Hills 2005) were also misidentified with xanthoid phenotypes of *B. rhodopurpureus* according to the accompanying illustrations which did not fit the species concept properly, even though A.E. Hills strenuously defended his collections as being the genuine *B. torosus* (Hills 2007). Simonini and Bertolini (2012) summarised past and current systematic, taxonomic and nomenclatural knowledge about *B. torosus* and furnished an exhaustive list of bibliographic sources.

Based on a preliminary molecular phylogenetic analysis, Assyov et al. (2015; http://boletales.com/phylogenetics/) ultimately transferred *B. torosus* and related species to the newly established genus *Imperator* Koller et al., designating the former as the type species. On the other hand, the recombination of *B. torosus* in the genus *Suillellus* Murrill by Blanco-Dios (2015) was made without any phylogenetic justification.

Before the erection of the genus *Imperator*, however, Bertolini and Simonini (2013), following the suggestion by Estadès and Deïana (2004), officially designated a neotype (iconotype) of *B. torosus* based on a colour plate published in Pilát and Dermek (1974), matching the modern concept of this species according to the historical interpretation of the Swiss and French authors. However, they did not select an epitype because despite various efforts, they were unable to find fresh material in the original site of collection. They wrote: “…only findings from the type locality (Sauvabelin) would represent the best solution for selecting a neotype; in the absence of such a circumstance (should it happen in the future it would allow an epitype to be eligible) we believe the best solution is to select the colour plate by Dermek in Pilát and Dermek as a neotype” (“…solamente raccolte dal “locus typicus” (Sauvabelin) consentirebbero la migliore soluzione per la creazione di un neotypus; in assenza di questa eventualità (che se si presenterà in futuro potrà consentire la definizione di un epitypus) crediamo che la migliore soluzione sia la creazione di un neotypus rappresentato dalla tavola a colori di Dermek, in Pilát and Dermek”) (Bertolini and Simonini 2013: 119). At that time, this was a problem of minor concern since the scientific community was unaware of the fact that *I. torosus* represented a species complex. At present, as more data accumulate, there is emerging evidence of the polyphyly of this species. Therefore, it is imperative to define the true *I. torosus*, and to delimit it clearly from the other species of the same complex. To resolve this, Simonini et al. (2025) designated an epitype; however, this creates a taxonomic conflict with Pilát and Dermek’s neotype illustration, as the plate and the newly selected epitype represent two distinct species.

In this study, we pursued the following goals: 1) perform a comprehensive taxonomic revision of the *I. torosus* species complex, including morphological variability, geographic distribution and ecology; 2) circumscribe the concept and taxonomic limits of *I. torosus* s. str. and introduce *I. occultus* as a new species; 3) elucidate the taxonomic status of the critical species *I. xanthocyaneus*; 4) assess the phylogenetic relationships among *Imperator* species through an internal transcribed spacer and the nuclear ribosomal large subunit (ITS-LSU) analysis, calculate pairwise distances (PD) and percent nucleotide similarity (PNS) for ITS, LSU, translation elongation factor 1-α (*tef*1), and DNA-directed RNA polymerase II subunit 2 (*rpb*2) loci; 5) discuss the inconsistency of the recently designated epitype of *B. torosus* with respect to: a) the original descriptions by Secretan and Fries, b) the historical interpretations and iconographic continuity by subsequent authors, c) the edaphic requirements, and d) the established neotype.

## Materials and methods

### Collection sites and metadata

The studied specimens of *Imperator* spp. and *Cyanoboletus poikilochromus* were collected from Bulgaria, Croatia, Czechia, France, Germany, Hungary, Italy, Romania, Slovenia, Spain, and Switzerland. They were deposited in public collections: CNF, LJF, K, PRM, SAF, SOMF, Z, and ZT (Thiers 2026) and in the private fungaria: “AB”, “AL”, “FR”, “CR”, “GS-”, “JG”, “JS”, “KW”, “MC”, “MG”, “MMB”, “PN”, and “FRJ”, which are maintained by Alona Yu. Biketova, László Albert, the Franck Richard team at CEFE, Claudio Rossi, Günter Saar, Jacques Guinberteau, Jürgen Schreiner, Karl Wehr, Marco Cartabia, Matteo Gelardi, Michal Mikšík, Paula Nimigean, and Renato Jonny Ferrari, respectively. Fungarium numbers, unless otherwise stated, are cited for all collections from which morphological features were examined. In the field, latitude, longitude, and elevation (geospatial references) were determined by Global Positioning System (GPS) receivers. Photographs of fungal collections were captured in natural habitat using digital cameras. Three types of data were recorded on site: (1) ecological and habitat observations including associated plant communities; (2) population size and grouping (collections of the same species that are 100 m apart are considered as different individuals of a population for distributional purposes); and (3) examination of the most relevant morphological features of the specimens to facilitate accurate identification. A few representative specimens were then collected and wrapped in aluminium foil, labeled and placed in storage containers. Once back from the foray, samples were examined as soon as possible; otherwise they were stored in a refrigerator at 4–8 °C and processed within 24 hours. For Croatian fungal collections soil pH was measured as follows: Soil samples were taken at a depth of about 5–10 cm and dried in the laboratory at room temperature of 20 °C. Roughly 50 g of dry soil was then dissolved in up to 2 dl of demineralized water in the beaker followed by filtration through a cheesecloth to remove insoluble debris. pH was then measured by using colour indicator strips according to the manufacturer’s instructions (MQuant, pH 2.0–9.0, with graduations of 0.5 pH units, Cat. No. 1.09584.0001; Merck KGaA, Darmstadt, Germany). The abbreviation “GP” (genetically proven) indicates the distribution of target *Imperator* species by countries based on collections whose identification was verified using DNA barcoding and phylogenetic methods. The metadata of most studied collections is given as follows: COUNTRY, First-Order Administrative Division: locality, coordinates (DMS), elevation (m), number of basidiomes and/or primordia collected, habitat with putative host plants, date (dd Mmm yyyy), collector’s name(s), collection number (additional collection number; GP if available). Names of all taxa and nomenclatural author citations throughout the text follow the Index Fungorum (2026). Geographic distribution of the studied species has also been checked in specialised literature and iNaturalist (2026). The acronym “GP” in species distribution stands for “genetically proven”.

### Climatic context

We compared the historical climatic context of Sauvabelin (Lausanne, Switzerland) in 1811–1847 with the instrumental context of the Castelvecchio/Caldaro epitype locality (South Tyrol, Italy) in 1977–2013. These 37-year windows place the reference years 1833 and 1999 in the same relative position, with 22 preceding and 14 following years. The comparison describes the climatic contexts of the two periods relevant to the historical account and the epitype.

For Sauvabelin, daily mean temperature and precipitation were extracted from the published 1 km reconstructions of Imfeld et al. (2022, 2023), and daily maximum temperature from Imfeld and Brönnimann (2025, 2026). We used the grid cell nearest the focal coordinates reported by Simonini et al. (2025), 46.531389°N, 6.642500°E: LV95 E 2538500, N 1153500 (46.529968°N, 6.637037°E), approximately 447 m from the focal point. The source series were CH_temp_EnKF, CH_precip_QMAP and CH_tmax_EnKF (Tmax version 1.1), selected by the same spatial coordinates in their respective grids. The MeteoSwiss 1991–2020 mean-temperature normal at the same cell provided modern Swiss context.

For Castelvecchio, we used the official, manually corrected Monticolo/Montiggl station products (89100MS; 530 m a.s.l.) for monthly and annual temperature and precipitation in 1977–2013 (Provincia Autonoma di Bolzano [Meteo Alto Adige] n.d.). Monticolo lies approximately 6.51 km away from the epitype coordinates (46.382500°N, 11.238333°E; 600 m a.s.l.), only 70 m lower, and its historical series includes 1999. This combination of proximity, similar elevation and coverage of the epitype period makes it an appropriate primary observational proxy. We recalculated Monticolo Tmean by averaging all available published year-specific monthly and annual values for 1977–2013 and retained the provider’s published multiannual summaries for Tmax and precipitation; available records comprise 34 August temperature values, 33 annual temperature values and 37 years of precipitation. Missing observations were not imputed.

For the Swiss reconstructions, monthly and annual temperature indices were calculated as means of daily values and precipitation as sums of daily amounts, followed by averaging the 37 monthly or annual results. Tmax denotes the mean of daily maximum temperatures over the specified month or year, not an absolute temperature extreme. Monticolo’s monthly mean temperature is defined by the provider as the average of mean daily minimum and maximum temperatures. Values are reported to one decimal place, with rounding performed only after calculating means and contrasts. The comparison is descriptive; daily observations were not treated as independent replicates for significance testing. Complete monthly climatic summaries are provided in Suppl. material 2: table SS3.

### Geologic-pedologic data acquisition

We compiled and compared geologic-pedologic baseline evidence for the protologue locality (Sauvabelin, Lausanne, Switzerland), the epitype locality (Castelvecchio/Caldaro, South Tyrol, Italy), and the southeastern Slovenia near-sympatry test area (Podlesje vs. Stari Trg ob Kolpi). For each locality, we combined (i) official geological mapping products and portals, (ii) national or regional soil survey and geochemical baselines where available, and (iii) harmonised point-based soil-property estimates from a common pan-European/global framework, with the aim of characterising parent material context, base-status tendencies, and skeletal versus stone-poor soil structure in a way that is comparable across countries.

#### Swiss baseline (Sauvabelin/Lausanne)

Regional geologic-pedologic context for the Lausanne–Sauvabelin frame was documented from Swiss sources and complemented by SoilGrids point estimates at the published focal coordinate. The WSL Long-term Forest Ecosystem Research (LWF) Lausanne plot (Bois-du-Benenté), approximately 5.9 km away from the focal point, was used as a measured regional forest-soil reference (Walthert et al. 2003: 146–153; WSL LWF [Lausanne]). Its investigated profile is a deep, weakly pseudogleyed Dystric Cambisol developed in Würm moraine within a regional Molasse setting. The broader Swiss topsoil context was taken from the Geochemical Soil Atlas of Switzerland (Reusser et al. 2023).

#### Italian baseline (Castelvecchio/Caldaro)

Regional geological context was taken from the official South Tyrol forest manual, Waldtypisierung Südtirol, Band 2 (Autonomous Province of Bolzano–South Tyrol 2010: 169–171). The published epitype coordinate was intersected with the official ForestTypes vector layer (p_bz-Forestry:ForestTypes; equivalent scale 1:25,000; EPSG:25832), and the corresponding Bu2 forest-type sheet was used for its substrate and soil descriptions. The regional geological sequence and the soil description of the mapped forest type were considered separately.

#### Soil-property quantification by point extraction

To quantify and standardize pedologic contrasts across localities, we extracted point-level soil-property estimates from ISRIC SoilGrids (2026) v2.0 (250-m resolution) using the published REST properties/query endpoint, and checked the median values against the corresponding official Web Map Service layers. Four fixed focal points were used: Sauvabelin, Switzerland (46.531389°N, 6.642500°E), the presumptive original-locality point published by Simonini et al. (2025); Castelvecchio/Caldaro, Italy (46.382500°N, 11.238333°E), the epitype locality; Podlesje, Slovenia (45.503000°N, 14.992167°E), the *I. occultus* locality (LJF 8893); and Stari Trg ob Kolpi, Slovenia (45.515444°N, 15.078167°E), the *I. torosus* locality (CNF 10/320). Predictions for pH in water (phh2o) and coarse-fragment content (cfvo) were retrieved for the six standard depth intervals (0–5, 5–15, 15–30, 30–60, 60–100 and 100–200 cm). Median predictions (Q0.5) were used throughout. Returned integer values were divided by 10 (d_factor = 10), yielding pH units for phh2o and cm^3^/100 cm^3^ (vol.%) for cfvo. These modelled values were summarised as near-surface indicators (0–5 cm) and as depth-profile tendencies, and interpreted alongside the regional geological sources (Poggio et al. 2021; ISRIC SoilGrids 2026). Complete depth-resolved values are provided in Suppl. material 2: table SS2.

#### Pedologic terminology

Soil-profile classifications and forest-type soil descriptions were retained in the terminology of their respective sources (Walthert et al. 2003; Autonomous Province of Bolzano–South Tyrol n.d. [Bu2 type sheet]).

#### Southeastern Slovenia comparison

The Podlesje and Stari Trg ob Kolpi localities were considered within the broader Dinaric carbonate-karst region using the Geological Survey of Slovenia GIS portal (GeoZS) and the World Karst Aquifer Map (WOKAM). The same SoilGrids point-extraction workflow was applied to both points to keep soil-property descriptors directly comparable to the Swiss and Italian localities.

### Vegetation and fungal-assemblage context

Historical habitat evidence for Sauvabelin was taken from Secretan (1833, III: 24–25). Later vegetation evidence was considered separately, using Galland’s (1987) Flon-valley survey, the official Vaud forest-phytosociology point layer and the NaiS VD-36 Sauvabelin plot (ILEX 2025). We retrieved the public phytosociological points within 1.5 km of the Sauvabelin focal coordinate published by Simonini et al. (2025; 46.531389°N, 6.642500°E) from layer 616, vd.phytosociologie_points, and interpreted their codes using the cantonal typology (Clot and Delarze 2009; État de Vaud n.d. [Phytosociologie forestière vaudoise], data retrieved 3 September 2026). These later sources provide locality and regional context; they were not used to reconstruct the vegetation of 1833.

For Castelvecchio, the local account of prevalent trees and accompanying boletes was taken directly from Simonini et al. (2025: 32). Independent landscape context was obtained from the official South Tyrol potential natural forest-type layer (ForestTypes; 1:25,000). We identified the polygon intersecting the epitype coordinate (46.382500°N, 11.238333°E; 600 m) and calculated forest-group and forest-type areas in non-overlapping 0–100, 100–250 and 250–500 m annuli in ETRS89/UTM zone 32N. Percentages refer to mapped forest within each annulus; mapped coverage was calculated separately. Types were interpreted using the official type sheets and Waldtypisierung Südtirol, Band 2 (Autonomous Province of Bolzano–South Tyrol 2010 [Waldtypisierung Südtirol, Band 2]; Autonomous Province of Bolzano–South Tyrol n.d. [Bu2 type sheet]; data retrieved 3 September 2026).

The fungal comparison was restricted to the six accompanying species listed by Simonini et al. (2025: 32) and official SwissFungi records explicitly naming Sauvabelin in their locality or habitat descriptions (Gross et al. 2024; atlas queried 3 September 2026). The 5 × 5 km atlas cell containing the published focal point was searched (NETID 518; LV03 cell centre E537500, N152500), including relevant synonyms; generalised cell coordinates alone were not accepted as locality confirmation. The Slovenian comparison used field observations associated with the molecularly identified vouchers LJF 8893 (*I. occultus*, Podlesje) and CNF 10/320 (*I. torosus* s. str., Stari Trg ob Kolpi), and the great-circle distance between their recorded coordinates.

### Morphological studies

Macroscopic descriptions, macro-chemical reactions (Melzer’s reagent, Baral’s iodine, lugol, Guaiac, aniline, phenol, 25% NH_4_OH, 20% KOH, FeSO_4_) and ecological information, such as habitat notations, time of fruiting and associated plant communities are derived from detailed field notes of the fresh basidiomes (except for Swiss collections). Colour codes were taken from Kornerup and Wanscher (1978). Macro-chemical tests were carried out on fresh material. Spore prints from mature basidiomes were also obtained whenever possible. Specimens were then dried with an electric field air dryer at a temperature of ca. 50 °C in a climate-controlled room and packed in plastic zipped bags with or without small amounts of silica gel. Microscopic features were observed and recorded from revived dried material; sections were rehydrated either in water, 5% potassium hydroxide (KOH) or in anionic solution saturated with Congo red. All microscopic structures were observed and measured from preparations in anionic Congo red or 5% KOH. Colours and pigments were described after examination in water and 5% KOH. Measurements were made at 1000× using a calibrated ocular micrometer (Nikon Eclipse E200 optical light microscope). Basidiospores were measured from spore prints and/or directly from the hymenophore of mature basidiomes; dimensions are given as (absolute minimum) average ± standard deviation (absolute maximum), Q = length/width ratio with the extreme values in parentheses, Qm = average quotient (length/width ratio) ± standard deviation and average spore volume was approximated as a rotation ellipsoid [V = (π.L.W^2^) / 6 ± standard deviation]. The notation [n/m/p] indicates that measurements were made on “n” randomly selected basidiospores from “m” basidiomes of “p” collections. The width of each basidium was measured at the widest part, and the length was measured from the apex (sterigmata excluded) to the basal septum. Radial and/or vertical sections of the pileipellis were taken midway between the center and margin of the pileus. Sections of the stipitipellis were taken from the upper part along the longitudinal axis of the stipe. Metachromatic, cyanophilic and iodine reactions were tested by staining the basidiospores in brilliant cresyl blue, cotton blue in lactophenol and Melzer’s reagent (https://www.titolchimica.it) (Imler 1950; Clémençon 2009), respectively. Light micrographs were obtained via Micro Capture 6.9.12 Imaging software using a 5.0 Mp digital camera from Diagnostic Instruments. Line drawings of microstructures were traced in free hand based on digital photomicrographs of rehydrated material.

### DNA extraction, PCR amplification and DNA sequencing

DNA extraction and PCR amplification were performed from dried basidiomes using either a modified CTAB protocol, NucleoSpin Plant II (MACHEREY NAGEL GmbH & Co. KG, Düren, Germany), or REDExtract-N-Amp Plant PCR kits (Sigma-Aldrich, St. Louis, MO, USA) as described in previous publications (Martin and Rygiewicz 2005; Loizides et al. 2019; Biketova et al. 2022). Primers ITS1F, ITS1, ITS2, ITS3, ITS4, ITS4B, ITS5, and NLB4 were used for amplification of the ITS region (White et al. 1990; Gardes and Bruns 1993; Martin and Rygiewicz 2005); LR0R, ITS4Brev, LR5, and LR6 – for partial LSU (D1/D2 domains) (Vilgalys and Hester 1990; Cubeta et al. 1991; Hopple and Vilgalys 1999; Fachada et al. 2023); EF1-983F and EF1-1567R – for partial *tef*1 (1–2 domains) (Rehner and Buckley 2005); as well as bRPB2-6F and bRPB2-7R2 – for partial *rpb*2 (6–7 domains) genes (Matheny 2005; Matheny et al. 2007). PCR products were sent to ALVALAB (Oviedo, Spain), LGC Genomics (Berlin, Germany), Eurofins Genomics (Ebersberg, Germany), and Microsynth (Balgach, Switzerland) for sequencing. Sequences were manually edited and assembled using Sequencher 5.4.6 (Gene Codes Corporation, Ann Arbor, Michigan, USA), Geneious Prime 2025.2.1 (Dotmatics, Bishops Stortford, England, UK), or CodonCode Aligner 9.0.2 package (CodonCode Corp., Centerville, Massachusetts, USA).

### Sequence alignment, phylogenetic, pairwise distance, and percent nucleotide similarity analyses

A total of 107 sequences (94 ITS and 17 LSU) from 94 specimens (collections), including 76 newly generated (64 ITS, 4 ITS-LSU, and 8 LSU) and 31 retrieved from INSDC and UNITE databases (26 ITS and 5 LSU), were used in the multilocus phylogenetic analyses (Suppl. material 1). *Cyanoboletus poikilochromus* was chosen as an outgroup taxon due to certain morphological similarity with *I. torosus*, and also based on its belonging to the same subfamily *Suillelloideae*. Another outgroup taxon was *Rubroboletus sinicus* – the type species of the closest genus to *Imperator* based on phylogenetic inference (http://boletales.com/phylogenetics/). An ITS-LSU alignment was generated in MAFFT 7 (Katoh et al. 2019) using algorithm E-INS-i, and later manually adjusted.

In both ML and BI analyses, the GTR+G model with four partitions (ITS1, 5.8S, ITS2, LSU) was employed. The ML phylogenetic analyses were run in the raxmlGUI 2.0 (Edler et al. 2021), estimating unique model parameters for alignment and using 1000 rapid bootstrap replicates (BS) and branch lengths saved in the bootstrap trees (BS brL enabled). BI analysis was performed with MrBayes 3.2.7a software (Ronquist et al. 2012), with two parallel searches and four chains, with 10 mln generations, 40% burn-in, and a sampling frequency of every 1000^th^ generation.

The resulting phylograms were visualised in FigTree 1.4.4 (Rambaut 2018) and Inkscape 1.4 (Inkscape Project 2024; https://inkscape.org/de/). Only PP ≥ 0.9 and BS ≥ 50% values are reported in the resulting trees. Clades with PP ≥ 0.95 and BS ≥ 70% were considered as well-supported.

PD and PNS analyses were performed as additional species delimitation tools, to evaluate threshold values for DNA-barcoding in the genus *Imperator* (particularly between *I. torosus* and *I. occultus*), and to investigate barcoding gaps in the four most relevant marker loci: ITS, LSU, *tef*1 and *rpb*2 (Zhao et al. 2011; Garnica et al. 2016; Vu et al. 2019). Thereby, we also generated *tef*1 and *rpb*2 sequences from the holotype of *I. occultus* (CNF 10/34) and from CNF 10/249, the specimen intended for proposal as the conserved type of *Boletus torosus*. PD analysis for separate ITS, LSU, *tef*1, and *rpb*2 alignments was performed in MEGA 12 (Kumar et al. 2024) using the Maximum Composite Likelihood method, with standard errors (SE) estimated from 1,000 bootstrap replicates and all other settings left at their defaults. The ITS dataset comprised 86 sequences containing the ITS1, 5.8S, and ITS2 regions; the LSU, *tef*1, and *rpb*2 datasets comprised 14, 4, and 3 sequences, respectively. PD and SE values were multiplied by 100 and reported as percentages (%).

PNS was calculated from the same alignments using a custom Python script. For each pair of sequences, positions were compared site by site, considering only positions containing unambiguous nucleotides (A, C, G or T) in both sequences. Sites containing gaps, missing data, or ambiguous nucleotide codes in either sequence were excluded from both the numerator and the denominator. PNS was calculated as the number of identical comparable positions divided by the total number of comparable positions × 100 (%). The percentage of observed nucleotide differences was calculated as 100-PNS. Observed nucleotide differences and PNS values were summarised for each marker and taxon pair and are reported as ranges when more than one pairwise comparison was available. For within-taxon comparisons, self-comparisons and duplicate reciprocal comparisons were excluded.

## Results

### Phylogenetic, pairwise distance, and percent nucleotide similarity analyses

**Figure 1.**
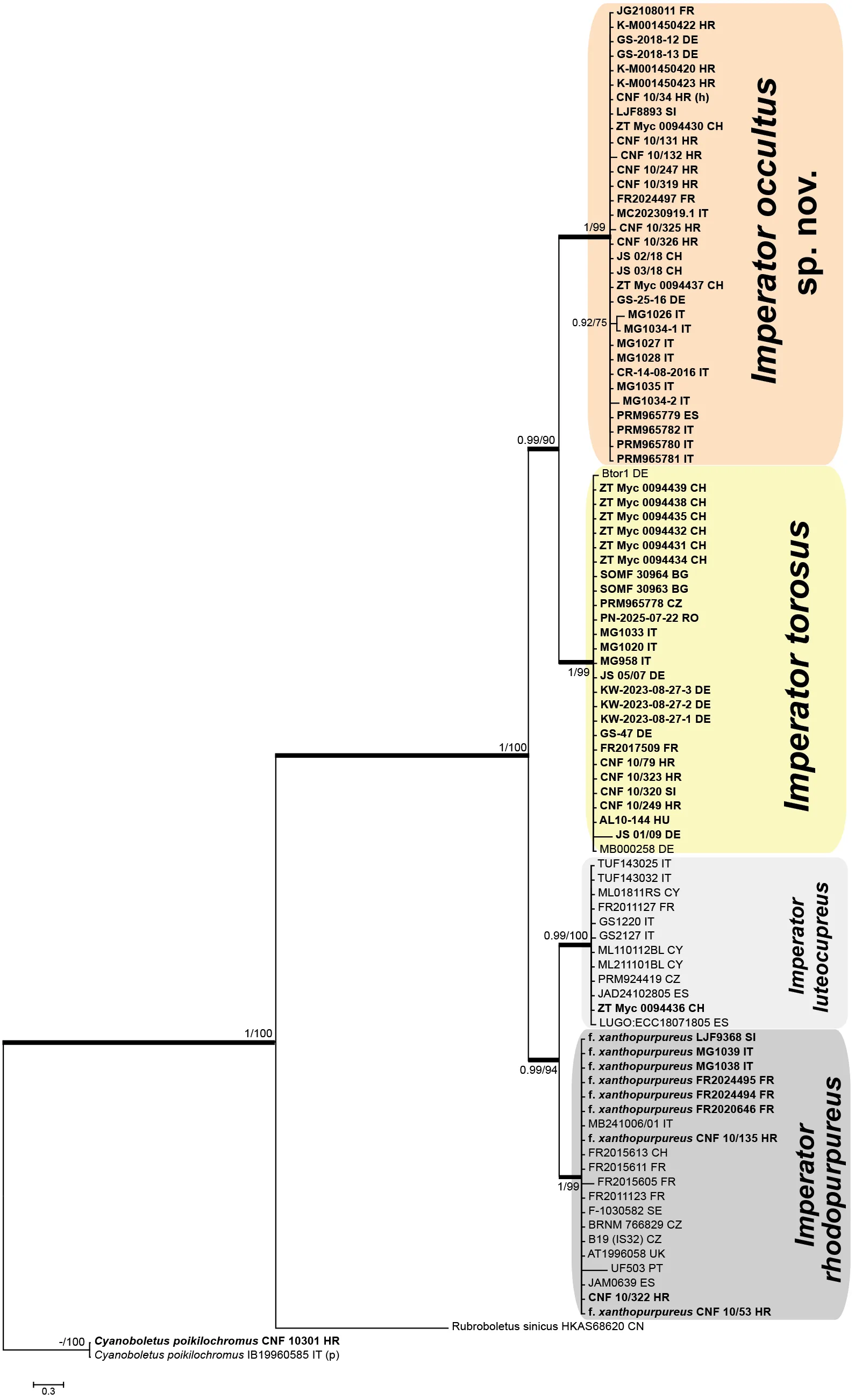
BI phylogenetic tree of *Imperator* generated from ITS-LSU dataset. PP values ≥ 0.9 and BS support values ≥ 50% are shown at the nodes. Thickened branches indicate PP ≥ 0.95 and BS support ≥ 70%. Newly sequenced collections are indicated in bold. Two-letter country codes (ISO 3166-1 alpha-2) reflecting the origin of specimens are given.

An ITS-LSU alignment consisted of 1958 aligned bases with gaps, including 144 (7.4%) parsimony informative sites (Suppl. material 3). The BI and ML analyses generated almost identical tree topologies with minimal variation in statistical support values; thus the BI tree with both posterior probability (PP) and bootstrap support (BS) values was selected for the purposes of display (Fig. 1).

*Imperator* forms a well-supported genus-level clade (PP = 1.00, BS = 100%), consisting of four species: three known and one newly described, *I. occultus*. *Imperator torosus* and *I. occultus* are sister species that form both a well-supported common branch (PP = 0.99, BS = 90%) and species-level terminal clades (PP = 1.00, BS = 99%). *Imperator rhodopurpureus* and *I. luteocupreus* are another pair of sister species that also form both a well-supported common branch (PP = 0.99, BS = 94%) and species-level terminal clades (PP = 1.00, BS = 99% and PP = 0.99, BS = 100%, respectively).

Results of the PD and PNS analyses of *I. torosus* and *I. occultus* are shown in Table 2. The ranges of interspecific differences calculated as PD and as 100-PNS were, respectively: 3.03–3.74% and 2.95–3.62% for ITS, 0.28–0.38% and 0.27–0.38% for LSU, 2.95–3.13% and 2.86–3.04% for *tef*1, and 1.87–1.97% and 1.84–1.93% for *rpb*2. The low interspecific divergence observed in partial LSU compared to other loci reflects the higher evolutionary constraint on structural rRNA genes relative to the non-coding ITS spacers and partial protein-coding *tef*1 (with introns) and *rpb*2. The ranges of intraspecific differences were in both cases (PD and 100-PNS): 0–0.43% for ITS, 0% for LSU, *tef*1, and *rpb*2. These results suggest that clear barcoding gaps exist in these species. However, a single intraspecific comparison for each of the *tef*1 and *rpb*2 genes was performed only for *I. torosus*. Future studies should aim at increasing the amount of sequenced collections in the four currently known *Imperator* species and obtain higher statistical robustness for all four loci.

**Table 2.** Percent nucleotide similarity (PNS), nucleotide differences (100-PNS), and pairwise distances (PD) for pairwise comparisons among target *Imperator* taxa by marker. PD and standard error (SE) values are expressed as percentages; SE ranges are shown where applicable.

**Marker**	**Taxon pair**	**No. of seq**.	**No. comparisons**	**Length of compared sequences (bp)**	**No. different positions (bp)**	**PNS (%)**	**100-PNS (%)**	**PD (%)**	**SE range in PD (%)**
**ITS**	*Imperator occultus* / *Imperator torosus*	32 / 25	800	606–678	19–23	96.38–97.05	2.95–3.62	3.03–3.74	0.72–0.83
	*Imperator occultus* / *Imperator occultus*	32 / 32	496	676–698	0–3	99.57–100.00	0.00–0.43	0.00–0.43	0.00–0.25
	*Imperator torosus* / *Imperator torosus*	25 / 25	300	628–685	0	100.00	0.00	0.00	0.00
**LSU**	*Imperator occultus* / *Imperator torosus*	3 / 9	27	799–1094	3	99.62–99.73	0.27–0.38	0.28–0.38	0.16–0.22
	*Imperator occultus* / *Imperator occultus*	3 / 3	3	903–931	0	100.00	0.00	0.00	0.00
	*Imperator torosus* / *Imperator torosus*	9 / 9	36	800–1111	0	100.00	0.00	0.00	0.00
***tef*1**	*Imperator occultus* / *Imperator torosus*	1 / 2	2	419–560	12–17	96.96–97.14	2.86–3.04	2.95–3.13	0.83–0.91
	*Imperator torosus* / *Imperator torosus*	2 / 2	1	421	0	100.00	0.00	0.00	0.00
***rpb*2**	*Imperator occultus* / *Imperator torosus*	1 / 2	2	599–673	11–13	98.07–98.16	1.84–1.93	1.87–1.97	0.53–0.55
	*Imperator torosus* / *Imperator torosus*	2 / 2	1	599	0	100.00	0.00	0.00	0.00

### Climatic context

The historical Sauvabelin reconstruction yielded an August mean temperature of 16.9 °C and a mean daily maximum of 21.3 °C, compared with the Monticolo means of 21.5 °C and 28.4 °C, respectively (Table 3). The corresponding August temperature contrasts were approximately 4.5 °C and 7.1 °C. Annual mean temperature and mean daily maximum were 8.9 °C and 12.1 °C at Sauvabelin, compared with 12.0 °C and 17.6 °C at Monticolo, respectively. Mean annual precipitation was 997.0 mm at Sauvabelin and 814.6 mm at Monticolo; August precipitation differed less, at 100.8 and 91.3 mm, respectively. The modern August mean-temperature normal at the Sauvabelin cell was 19.4 °C for 1991–2020 (MeteoSwiss [gridded normals]). Swiss values were calculated from complete daily reconstructions for all 37 years (Imfeld et al. 2022, 2023; Imfeld and Brönnimann 2025, 2026). Monticolo values are recalculated Tmean averages and the provider’s published Tmax and precipitation summaries, with 34 available August temperature years, 33 annual temperature years and 37 precipitation years (Provincia Autonoma di Bolzano [Meteo Alto Adige] n.d.). The periods describe historical Swiss and modern Italian climatic contexts. Monthly values are reported in Suppl. material 2: table SS3.

**Table 3.** Climatic context of historical Sauvabelin and the Castelvecchio epitype proxy, compared across 37-year reference windows.

**Variable**	**Sauvabelin, 1811–1847 (reconstruction)**	**Monticolo, 1977–2013 (station data)**
August mean temperature (°C)	16.9	21.5
August mean daily maximum temperature (°C)	21.3	28.4
August mean precipitation (mm)	100.8	91.3
Annual mean temperature (°C)	8.9	12.0
Annual mean daily maximum temperature (°C)	12.1	17.6
Annual mean precipitation (mm)	997.0	814.6

### Geologic-pedologic comparison

Regional sources place the Lausanne–Sauvabelin frame within the Swiss Plateau Molasse and associated Quaternary-cover setting (Walthert et al. 2003). At Castelvecchio, the published epitype coordinate intersects the official ForestTypes polygon Bu2. The forest-type description sheet describes medium-to-deep, base-poor, partly podzolised brown soils on silicate substrates, with moderate to very high skeletal content. The regional manual independently reports that Bolzano quartz porphyry reaches the Altenburg/Castelvecchio terraces (Autonomous Province of Bolzano–South Tyrol 2010: 169–171; Autonomous Province of Bolzano–South Tyrol n.d. [Bu2 type sheet]).

SoilGrids v2.0 median (Q0.5) predictions showed near-surface pH(H_2_O) of 5.6 at Sauvabelin and 6.4 at Castelvecchio. Across the six depth intervals spanning 0–200 cm, predicted pH ranges partly overlapped (5.6–6.2 and 5.8–6.7, respectively). Coarse-fragment predictions showed a stronger contrast: 5–9 vol.% at Sauvabelin versus 19–31 vol.% at Castelvecchio. At Podlesje and Stari Trg ob Kolpi, near-surface pH(H_2_O) was 5.8 and 6.1, respectively, while median coarse-fragment predictions ranged from 3.8–6.4 and 2.7–3.1 vol.%. Complete depth-specific values are provided in Suppl. material 2: table SS2.

### Vegetation and fungal-assemblage comparison

Secretan (1833, III: 25) described the Sauvabelin collection as growing under dense beeches in a moist place on the eastern slope. The Vaud query returned 108 points: 90 classified within the 100-series beech forests and 18 indeterminate. None of the sampled points was assigned to the 400-series thermophilous mixed forests or the 600-series oak forests. The modern NaiS VD-36 Sauvabelin plot (ILEX 2025) was classified as 7a/115, Hêtraie à Aspérule typique, with *Fagus sylvatica* representing 50% of its recorded tree composition. Galland’s (1987) later survey documented a heterogeneous beech–oak forest margin, including local dry-site elements; it did not identify a thermophilous oak stand at the published focal point.

Simonini et al. (2025: 32) reported prevalent *Fagus sylvatica* with *Quercus* spp., mainly *Q. pubescens*, in the Castelvecchio collecting area. The epitype coordinate intersected forest type Bu2, siliceous beech forest with *Luzula nivea*, described in the provincial typology as a submontane beech forest of shaded to moderately fresh sites within the South Tyrolean warmth-loving beech-forest group. Beech types accounted for 100.0%, 91.7% and 61.4% of mapped forest in the 0–100, 100–250 and 250–500 m annuli, respectively. The outer annulus also contained 30.9% manna-ash–hop-hornbeam and mixed-oak forest, 6.0% oak–pine forest and 0.7% oak forest. Mapped coverage was 100%, 100% and 97.1%, respectively. Types Bu6 and Bu4, representing drier or hop-hornbeam-associated beech settings, were already present within 100 m (22.7% and 2.6%).

The six associates listed by Simonini et al. (2025: 32) were *I. luteocupreus*, *Rubroboletus satanas* (Lenz) Kuan Zhao & Zhu L. Yang, *R. rhodoxanthus*, *R. lupinus*, *Suillellus luridus* (Schaeff.) Murrill and *Boletus aestivalis* Paulet. Of these, only *R. satanas* was represented in the official SwissFungi records explicitly naming Sauvabelin; the matching record was from 2013. The other five species were not documented for Sauvabelin in the locality-specific records examined. The two molecularly confirmed Slovenian collections were approximately 6.8 km apart; field observations placed *I. occultus* in a microsite with a thermophilous vegetation component and *I. torosus* s. str. in a mesic mixed forest.

### Taxonomy

#### Imperator torosus

Taxon classificationFungiBoletalesBoletaceae

(Fr.) Assyov, Bellanger, Bertéa, Courtec., G. Koller, Loizides, G. Marques, J.A. Muñoz, Oppicelli, D. Puddu, F. Rich. & P.-A. Moreau, Index Fungorum 243: 1. 2015.

CD2E0A82-10BC-5758-9838-378A5D9790CF

550512

Figs 2, 3, 7, 8a–d

Boletus torosus Fr. in Fries and Hök, Boleti, fungorum generis illustratio: 10. 1835. (Basionym)
≡ Dictyopus torosus (Fr.) Quél., Enchiridion fungorum in Europa media et praesertim in Gallia Vigentium: 160. 1886.
≡ Suillus torosus (Fr.) Kuntze, Revisio generum plantarum 3(3): 536. 1898.
≡ Boletus appendiculatus subsp. *torosus* (Fr.) Konrad, Bulletin de la Société Linnéenne de Lyon 1(9): 143. 1932.
≡ Tubiporus torosus (Fr.) Imler, Bulletin de la Société Mycologique de France 66(4): 179. 1950.
≡ Suillellus torosus (Fr.) Blanco-Dios, Index Fungorum 211: 2. 2015.
– Boletus torosus var. *xanthus* Cetto, I Funghi dal Vero, Vol. 4: 467. 1983. (nom. inval., Art. 39.1, Madrid)
– Boletus torosus var. *xanthus* Cetto, Enzyklopädie der Pilze, Band 1: Leistlinge, Korallen, Porlinge, Röhrlinge, Kremplinge: 575. 1987. (nom. inval., Art. 39.1, Madrid)
Boletus pachypus Fries 1815 s. Secretan 1833 (non Fr.), Mycographie Suisse, Vol. 3: 24. 1833 et auct. p. p. (Misapplied name)

##### Holotype.

Not indicated. No original material is known.

##### Neotype.

Pilát and Dermek, Hríbovité Huby (*Boletaceae* – *Gomphidiaceae*): Tav. 65d–f. 1974. Designated by Bertolini and Simonini (Rivista di Micologia 56(2): 120. 2013).

##### Epitype.

Italy • Caldaro, Castelvecchio (BZ), under *Fagus sylvatica*, 29 Aug 1999, G. Simonini, G. Bissaro, G. Ferrarese, Herbarium of the Civic Museum of Natural History of Venice, MCVE18453 (IF904107), designated by Simonini et al. (Rivista di Micologia 68(1): 33. 2025).

##### Conservation note.

**Figure 2.**
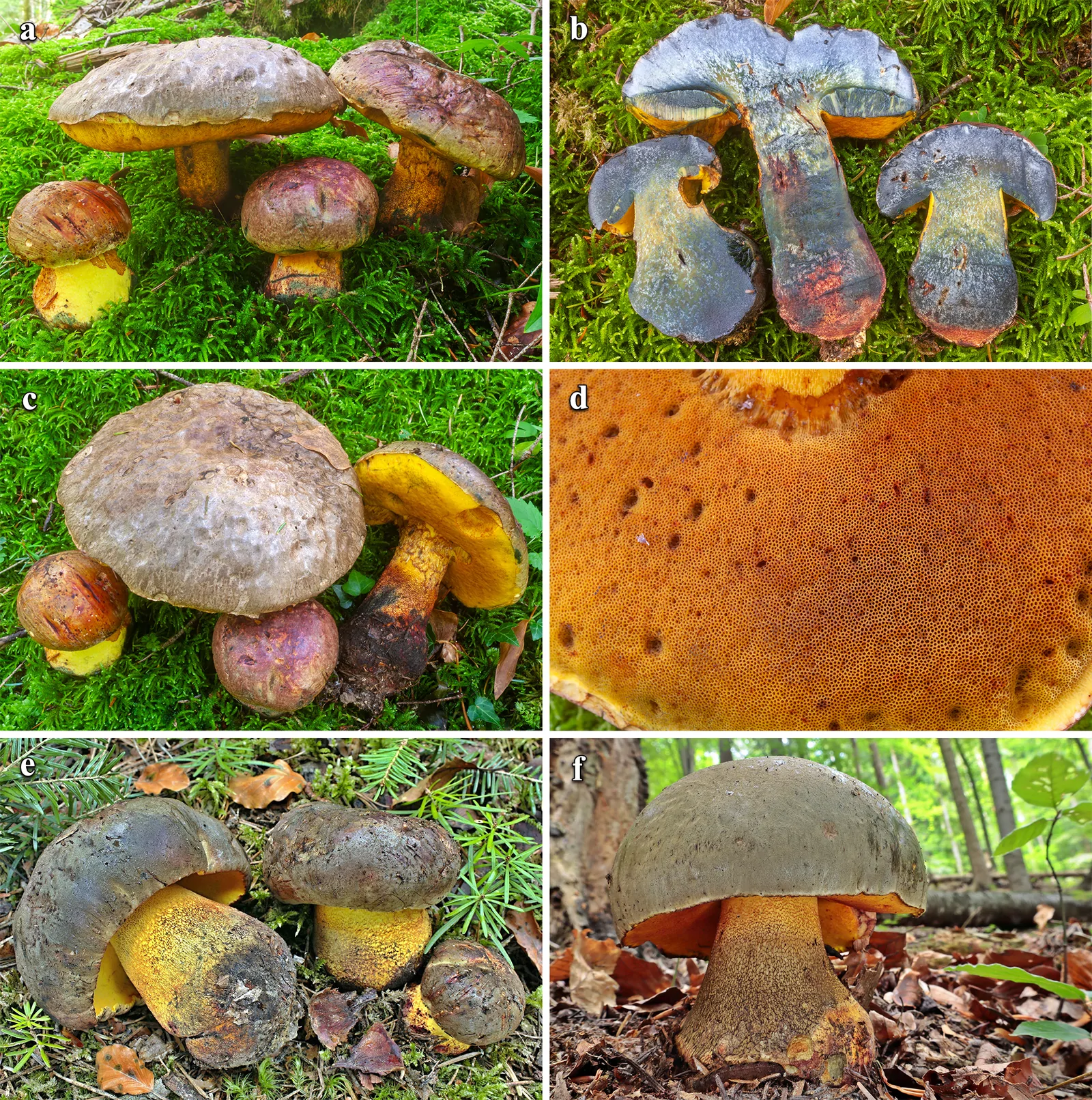
*Imperator torosus*, basidiomes in habitat. **a–d**. CNF 10/249, CROATIA, Primorje-Gorski Kotar County, Parg near Čabar (specimen intended for proposal as the conserved type); **e**. MMB35/2018, CZECHIA, Zlín region, Nový Hrozenkov; **f**. CZECHIA, Zlín region, Horní Lideč (specimen with orange-red pores). Photos: N. Ferenčak (**a–d**), M. Mikšík (**e–f**).

**Figure 3.**
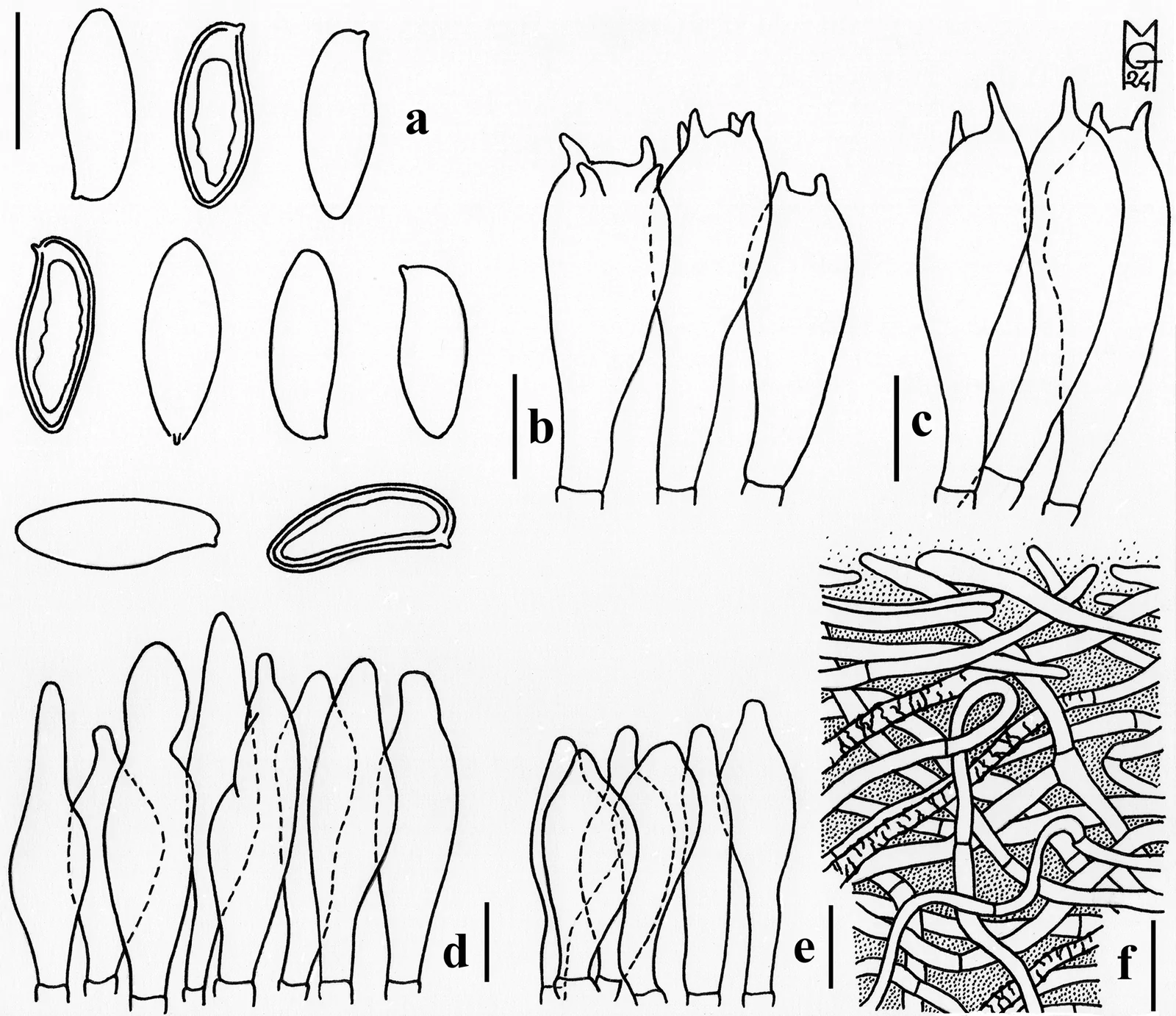
Microscopic features of *Imperator torosus* (CNF 10/249, specimen intended for proposal as the conserved type). **a**. Basidiospores; **b**. Basidia; **c**. Caulobasidia; **d**. Caulocystidia; **e**. Cheilocystidia; **f**. Pileipellis. Scale bars: 10 µm (**a–e**); 20 µm (**f**). Drawings by M. Gelardi.

CNF 10/249, documented photographically in Fig. 2a–d and microscopically in Fig. 3, is the specimen we intend to propose as the conserved type of *Boletus torosus* in a separate nomenclatural proposal. Sequence data were generated from CNF 10/249 for ITS, LSU, *tef*1, and *rpb*2 (Suppl. material 1).

##### Secretan’s (1833: 24) description (part of the protologue of *B. torosus* Fr.).

*23*. B. bulbeux *à poudre rose*. B. pachypus.

Fries Obs. Myc. 1, p. 118. Syst. Myc. 1, p. 390 (excl. syn.).

Chapeau. *Basane livide, taché de noirâtre dans les parties qui ont été touchées; il tourne ensuite au jaunâtre et se tessèle par compartiments brunâtres; il est d’abord mollasse; puis dur et redevient à la fin assez mou. Premièrement hémisphérique, il s’évase ensuite; le centre un peu déprimé; dans la vieillesse les bords se relèvent, même assez haut. La surface est raboteuse. Diam. allant à 7 ½ p. Chair d’abord jaune soufre, puis jaunâtre, verdissant et ensuite bleuissant à la cassure; épaisse de 15 à 18 l. Sous les tubes elle est jaune*.

Pores. *La couleur de jaunâtre dans l’enfance devient d’un beau jaune d’œuf; puis elle passe à un rouge faux, cramoisi clair à œil cendré. Cette teinte se répand comme une poussière sur la partie du pédicule qui est au-dessous (*Voy. l’observation*). A la fin, les pores prennent un rouge assez vif près du pédicule, se nuançant à l’orangé sur les bords du chapeau. Ils sont d’abord fermés, presque invisibles; à la fin ils ne présentent qu’une petite ouverture ronde, au milieu de l’extrémité large et aplatie du tube; ces sommités des tubes sont confluentes, irrégulières et presque labyrinthes. La longueur des tubes atteint 10 l.; ils sont à l’intérieur d’abord jaunes, puis verts*.

P. *Il ne forme qu’une grosse bulbe pointue au pied; sa hauteur est de 3 p., et son épaisseur de 2 ¼ à 3 p. Tant que le chapeau recouvre le pédicule comme une calotte, ce qui est sous les bords est jaune; la partie qui touche les bords est alors rouge pourpre. Le chapeau s’étant détaché, le sommet du pédicule est d’un jaune jonquille un peu brouillé de rouge; une zone pourpre, quelquefois assez vif, occupe la portion la plus épaisse de la bulbe; le bas se nuance à l’olivâtre. Un réseau brun à mailles serrées est apparent surtout dans le haut. Le pied est fixé en terre par une racine courte, courbée et munie de radicules nombreuses. La chair comme au chapeau, ne prend que tout au bas une légère teinte rouge. L’odeur est très-pénétrante. Ces plantes compactes, pesantes, croissent liées par le pied, sous les hêtres touffus, en lieu humide; vers la fin d’août. (*Sauvabelin; coteau oriental.)

*Obs. Cette poudre rouge, qui colore le dessous du bolet, est un cryptogame parasite appelé* mycogone rosea. Link in Berl. Mag. 1809, p. 18. Nees Syst. p. 44. Fries Syst. Orb. Veget. 1, p. 179. Pers. Myc. Eur. 1, p. 26.

##### Fries (in Fries and Hök 1835: 10), original diagnosis.

*† 24*. B. torosus*Fr., pileo pulvìnato scabro fuscescente, tactu nigro-maculato, stipite bulboso reticulato subradicato superne luteo purpurascente, tubulis liberis minutis luteis, dein rufescentibus. B. pachypus*, Secret. *excl. syn. In fagetis Helv. A priori certe differt; sequentibus magis affinis*. Pileus demum lutescens. Caro luteo-sulphurea fracta virescens, demumque coerulescens. Odor fortis.

##### Description.

***Ontogenetic development*** gymnocarpic. ***Basidiomes*** large, robust, dense and particularly heavy. ***Pileus*** 10.0–18.5 (22.0) cm broad, at first hemispherical then persistently convex and finally broadly pulvinate-flattened, sometimes slightly depressed at center, regularly to hardly unevenly shaped by shallow depressions all over the surface, decidedly fleshy, very firm and compact at the beginning but progressively softer with age; margin even to faintly wavy-lobed, initially bent towards the stipe or less often slightly involute, then curved downwards and finally completely plane, not or only a little extending beyond the tubes; cuticle not or only hardly peelable from pileus context and tending to tear apart together with the context underneath; surface matt, dry but slightly greasy to viscid with moist weather, usually not cracked but sometimes very slightly areolate from an earlier developmental stage in dry weather, revealing the white (2B1) to yellow (2B6) colour of the context between cracks; finely subtomentose-pruinose at first, more or less smooth and glabrous at maturity; pileus relief variable, sometimes remaining smooth throughout development, but sometimes becoming (or remaining) distinctly uneven and roughened, often bullate to undulate and from slightly rugulose to rugose (in places recalling the pileus surface of *I. rhodopurpureus*), whereas in other specimens such roughness may be entirely absent; variable in colour among collections and individuals, and also over time, but with a typical grey-cast leather-brown baseline that is leaden-grey (5F5) to leaden-brown (5E5) and may appear faintly olivaceous (4E4) to dark olivaceous (4E8); in young basidiomes the yellow ground (2B6) may remain visible in places, especially toward the margin or where the pruina is thin or absent, and this yellow underlayer can intermittently re-emerge through the darker palette. Contact-induced oxidation, which in nature can be triggered by ordinary mechanical contact and abrasion during emergence through litter, leaves and small twigs, by animal activity, or by the impact of precipitation, and which represents a late visible phase of the oxidative sequence that follows intense indigo-blue bruising (20F8), may yield scattered reddish (8D8) to reddish-brown (8E8) spots or streaks, typically irregular and sporadic in distribution and extent. In extremely rare cases, predominantly yellow (xanthoid/depigmented) individuals occur, with the pileus usually remaining pale yellow (4A6) to yellow-ochraceous (4C6) and only locally developing scattered reddish (8D8) to reddish-brown (8E8) spots or streaks, mostly at maturity; instantly and intensely bluing (20F8) on handling, touching or when injured, soon turning almost black (19F8); subcuticular layer yellowish (2B6) then whitish (2B1) with age. ***Tubes*** at first extremely thin in cross section then increasingly broader with age and shorter than the thickness of the pileus context (up to 2.2 cm long), adnate to adnexed but soon depressed around the stipe apex, bright yellow (3B8) at first, then yellowish-olive (3C8) and finally olive green (3D8), immediately turning dark blue (20F8) when cut and later fading to copper orange (7C6) or copper red (7D6); easily to moderately hardly detachable from the pileal context above. ***Pores*** initially concave then forming a flat surface, later convex-ascending, at first very small, then gradually wider with age (up to 1 mm in diam.), simple, roundish, becoming barely angular and very slightly elongated at maturity; in most cases concolourous with the tubes, yellow (3B8) from the beginning and usually remaining so, occasionally becoming orange (7A6) to red (8A6), particularly in aged basidiomes and in humid weather, shading from yellow-orange (5A4) to orange (5A6) towards the pileus margin; quickly turning dark blue (20F8) to very dark blue (19F8) or nearly black (18F8) on bruising or when injured and later fading to copper orange (7C6), copper red (7D6) or rusty brown (7E7). ***Stipe*** (6.1) 7.5–12.2 (12.7) × 3.2–6.7 cm, shorter than or as long as the pileus diameter at maturity (in primordia sometimes broader than the pileus diameter), central to slightly off-center, solid, firm, dry, straight or faintly curved, at first ventricose-ovoid to ventricose-fusiform, later cylindrical, equal but slightly swollen towards the base to decidedly clavate, ending with a short taproot at the very base; ground colour bright yellow (3B8) to golden yellow (4C8) throughout and sometimes with reddish purple (10E7) zones at the base, brownish lengthwise (7D4) in aged specimens; stipe surface showing a fine to moderately pronounced concolourous reticulum initially restricted to apex, later covering the upper half or the upper three quarters of the stipe and in aged specimens extending all the way down to the base, with small, roundish to barely angular and regularly arranged, isodiametric meshes, more elongated towards the base, concolourous with the ground then whitish (8C1) with age but becoming dark reddish purple (10F6) in wet weather; rapidly turning dark blue (20F8) to almost black (18F8) when pressed and later fading to copper orange (7C6) or copper red (7D6); ***basal mycelium*** and ***rhizomorphs*** whitish beige (4C3) to cream (3C1). ***Context*** firm and tough when young, later soft textured and eventually flabby in the pileus, a little more fibrous in the stipe; evenly yellow (3C4) to yellowish white (3C2), whitish (3B1) in old specimens but usually reddish purple (10F6) towards the stipe base, in old specimens the reddish purple colour can spread all the way up to the pileus; instantly and violently turning dark blue (20F8) when exposed to air (excluding the reddish-brownish (8E8) basal part), fading to gray (18E3) in ten minutes and after a few hours becoming dark copper red (7D6); subhymenophoral layer yellow (3C4) to whitish (2B1); in exsiccate brown (6D8), dull reddish brown (8E8) to dark brown (7F7). ***Odour*** in young specimens agreeable, vaguely fruity although little pronounced but soon becoming unpleasant, of expired medicines, pharmaceutical lab or iodoform-like. ***Taste*** mild, slightly sour. ***Spore print*** raw umber (5F8).

***Macrochemical spot-test reactions***: 5% and 20% KOH: staining yellow-orange or orange to vinaceous red on pileus, bright orange on context and stipe, orange to vinaceous red on hymenophore; Melzer: nil or staining dark greenish blue on context, dark blue on hymenophore, nil or light to dark green on pileus, nil on stipe; Baral: nil or staining dark greenish on context, dark blue on hymenophore, nil or light to dark green on pileus, nil on stipe; Lugol: nil or staining dark greenish blue on context, dark blue on hymenophore, nil or light to dark green on pileus, nil on stipe. Guaiac: immediately dark blue on pileus and stipe, dark blue on hymenophore, nil on context; Aniline: staining light orange or nil on pileus and context, nil on hymenophore, nil to light yellow on stipe; Phenol: nil on pileus and context, dark blue-green on hymenophore, light blue on stipe in 30 sec; FeSO_4_: nil or gray-green to dark green in 20 sec on pileus, immediately olivaceous green to dark blue-green on hymenophore and stipe, nil to gray-green or yellow-green then sordid olivaceous green on context (the cyanescence of context disappears); 25% NH_3_: light orange, orange to vinaceous red without extended halo on pileus, orange-red to reddish brown or dark brown on hymenophore, light orange (with extended halo) on context, light orange to reddish brown or dark brown on stipe.

***Basidiospores*** [420/17/10] (10.5) 13.8 ± 1.0 (17) × (4.5) 5.6 ± 0.3 (7.5) μm, Q = (1.68) 1.79–3.02 (3.07), Qm = 2.48 ± 0.21, V = 227 ± 40 μm^3^, fairly uniform in dimension, inequilateral, ellipsoid to ellipsoid-fusiform or rarely allantoid in side view, ellipsoid to ellipsoid-fusiform in face view, smooth, apex rounded, with a short apiculus and with a shallow suprahilar depression or applanation and with an adaxial swelling, moderately thick-walled (0.3–0.7 μm), golden yellow coloured in water and 5% KOH, having one or two large oil droplets when mature, rarely pluri-guttulate, inamyloid, acyanophilic and with a very faint metachromatic reaction. ***Basidia*** 26–35 × 10–14 μm (n = 12), shortly cylindrical-clavate to clavate, moderately thick-walled (0.5–1.0 μm), predominantly 4-spored but also 1- or 2-spored, usually bearing relatively short sterigmata (2–5 μm), pale yellowish and containing straw-yellow oil guttules in water and 5% KOH, bright yellow (inamyloid) in Melzer’s, without basal clamps; basidioles shortly cylindrical-clavate to clavate, similar in size to basidia. ***Cheilocystidia*** (18) 24–33 (43) × 5–12 μm (n = 15), very common, moderately slender, projecting straight to sometimes flexuous and protruding from the hymenial palisade, irregularly cylindrical or cylindrical-fusiform to fusiform or sublageniform, rarely ventricose-fusiform, short mucronate to subclavate, with rounded to subacute tip, sometimes with a secondary septum or multiseptate, smooth, moderately thick-walled (0.5–1.0 μm), pale yellow in water and 5% KOH, bright yellow (inamyloid) in Melzer’s, without epiparietal encrustations. ***Pleurocystidia*** 25–38 (54) × 5–10 μm (n = 6), very rare and dispersed, shape, size, colour and chemical reactions like cheilocystidia or slightly longer, often with a secondary septum or multiseptate. ***Pseudocystidia*** not observed. ***Pileipellis*** an ixocutis to less frequently an ixotrichoderm tending to ixocutis, consisting of loosely interwoven to subparallel, elongated, filamentous and sinuous, frequently branched hyphae embedded in gelatinous matter; terminal elements 27–176 × (2) 3–8 μm, long and slender, cylindrical, apex rounded-obtuse or sometimes pointed, moderately thick-walled (up to 1 μm), hyaline to more often straw yellow or golden yellow in water and 5% KOH, inamyloid in Melzer’s, smooth to less frequently ornamented by a subtle granular or zebra-like, dark brown to blackish epiparietal encrustation which tends to dissolve in KOH; subterminal elements similar in shape, size and colour to terminal elements. ***Stipitipellis*** a layer of strongly intermingled and irregularly arranged, frequently branching, smooth-walled hyphae, 2–10 μm wide, hyaline to yellowish in water and 5% KOH; the stipe apex is covered by a well-developed caulohymenial layer consisting of sterile caulobasidioles, scattered, predominantly 1- or 2-spored but also 4-spored, fertile caulobasidia, 29–39 (41) × 10–15 μm, sterigmata 2–5 μm long (n = 13) and abundant projecting ***caulocystidia*** similar in shape and colour to hymenial cystidia but usually slightly wider, 28–53 × 8–16 μm (n = 20), having a wall up to 1 μm thick. ***Lateral stipe stratum*** under the caulohymenium present although obscurely differentiated from the stipe trama and somewhat thin, of the “boletoid type”, at the stipe apex a 10–30 μm thick layer consisting of divergent, inclined and running towards the external surface, loosely intermingled and branched hyphae remaining separate and embedded in a gelatinous substance. ***Stipe trama*** composed of irregularly and densely arranged, strongly interwoven, filamentous, smooth, inamyloid hyphae, 2–8 μm broad. ***Hymenophoral trama*** bilateral divergent of the “*Boletus*-type”, with slightly divergent, moderately recurved-arcuate and loosely arranged hyphae, lateral stratum hyphae diverging at an angle of 30° to the mediostratum but tending to become subparallel with the mediostratum in aged specimens, hyphae frequently branched, shortly to moderately distantly septate and generally not restricted at septa, gelatinous (lateral strata hyphae in transversal section not touching each other, (3) 4–10 (12) μm apart, 2–12 μm broad), hyaline to pale yellowish in water and 5% KOH, inamyloid in Melzer’s; lateral strata (10) 15–50 (60) μm thick, mediostratum (10) 20–50 (60) µm thick, axially arranged, consisting of a tightly adpressed, non-gelatinous bundle of hyphae, 2–9 µm broad, shortly septate; in Congo Red the mediostratum is darker than the lateral strata. ***Thromboplerous hyphae*** very common in all tissues, thick-walled, rarely septate, melanized, with a dense golden yellow homogeneous content in 5% KOH. ***Clamp connections*** absent in all tissues.

##### Edibility.

Certainly poisonous (gastrointestinal syndrome) if eaten raw. It is considered by some authors edible and choice after prolonged cooking (Kühner and Romagnesi 1953; Piane 1966; Raris 1970a, 1970b; Marchand 1973), but several reports of gastroenteritis have been documented even after adequate heat treatment (Merlet 1997; Fourré 1998a, 1998b, 2006). *Imperator torosus* has also been suspected to provoke coprinic syndrome in association with alcohol consumption (Kiwitt and Laatsch 1994; Flammer and Horak 2003; Flammer 2008), but confirmed cases are not documented in the cited literature and the toxicological significance of the reported coprine remains uncertain (Sitta et al. 2021). Anyway, it is recommended not to consume this species, especially because evaluations on edibility/toxicity are severely affected by the mixed taxonomic nature of *I. torosus* s. l. records in the cited literature, which add further confusion to the already existing uncertainty. In this regard, the aforementioned reports of poisoning by Merlet (1997) and Fourré (1998a, 1998b, 2006) are most likely to be referred to as *I. occultus* (see discussion below).

##### Ecology and phenology.

Solitary to gregarious, growing on calcareous soil (soil pH 6.5), preferably in continental or subalpine environments, among litter under coniferous and deciduous trees, reported to date with *Abies* spp. (*A. alba*), *Picea abies* (*Pinaceae*), *Fagus sylvatica* (*Fagaceae*) and perhaps *Corylus avellana*, at elevations from 378 to 1399 m (this study), extending up to 1653 m at Gerede, Hacı Veli High Plateau (40.817990°N, 32.184118°E; Gürzoğlu et al. 2024, based on the GPS coordinates provided in the paper). Occurring from late June to October. Mikšík et al. (2025) documented a locality of *I. torosus* in Thaur (Austria) from a mountain meadow where only *P. abies* and *C. avellana* were present nearby. Based on repeated observations by various authors (Rainer Wald, Jiří Polčák pers. comm., Michal Mikšík pers. obs.), it is worth noting that other rare bolete species, namely *Butyriboletus roseogriseus* (Šutara, M. Graca, M. Kolařík, Janda & Kříž) Vizzini & Gelardi and *Rubroboletus rubrosanguineus* (Walty ex Cheype) Kuan Zhao & Zhu L. Yang (Schreiner 2014; Šutara et al. 2014), very often grow sympatrically alongside *I. torosus*.

##### Known distribution.

(the following list is based only on reports we assume to be referred to *I. torosus* s. str.) Austria (Sperdin 1984; Peintner et al. 1999; Noordeloos 2010; Klofac 2007, 2011; Dämon and Krisai-Greilhuber 2017; Neukom 2018; Noordeloos et al. 2018; Klofac and Krisai-Greilhuber 2020; Dämon et al. 2023; Friebes 2025; Mikšík et al. 2025), Bosnia and Herzegovina (Omerović and Jukić 2011; Jukić et al. 2019), Bulgaria (Assyov and Denchev 2004, 2010; Gyosheva et al. 2006; Denchev and Assyov 2010; GP – this study), Croatia (Tkalčec and Mešić 2003; this study, GP), Czechia (Pilát and Dermek 1974; Hlaváček 1983; Šutara et al. 2014; Mikšík 2017; Polčák 2019; Zíbarová et al. 2024; Mikšík et al. 2025; GP – this study), France (Quélet 1886, 1888; Barbier 1904; Gilbert 1936; Metron 1963; Piane 1966; Lannoy and Estadès 2001; Estadès and Deïana 2004; Estadès and Lannoy 2004; Chéreau et al. 2012; Reumaux and Carteret 2013; Page et al. 2021; Mancari 2026; GP – this study), Germany (Migula 1912; Krieglsteiner 1984a, 1984b, 2000; Zehfuß et al. 2000; Röger 2004, 2007, 2009; Schreiner 2008; Karasch and Hahn 2009; Krieglsteiner 2010; Reichert and Reil 2012; Noordeloos et al. 2018; Winkler and Keller 2023; Wald 2024; Mikšík et al. 2025; GP – Binder and Hibbett 2006, Wu et al. 2022 and this study), Greece (Konstantinidis 2009 (as “*Boletus fechtneri*”), 2025), Hungary (Babos 1989; Albert 2011; GP – this study), Italy (Cetto 1970; Raris 1970a; Alessio 1985; Cazzoli 1991; Foiera et al. 1993; Simonini 1998; Alpago Novello 2006; Padovan 2009; Giolo 2013; Papetti et al. 2013; Ferrarese 2019; Siniscalco et al. 2019b; Gasparini 2024; GP – this study), Liechtenstein (Prongué et al. 2004), Luxembourg (Tholl 1985), Montenegro (Hadžić 1995; Kasom and Miličković 2010; Kasom and Karadelev 2011; Kasom 2013), Romania (Anghelescu and Costa 2023; GP – this study), Russia (Vaasma et al. 1986; Zamotajlov et al. 2012; Sarkina 2020a, 2020b; Sirotyuk and Shadge 2023), Slovakia (Dermek and Pilát 1974; Kautmanová 2008; Mikšík 2015, 2017; Kunca 2016; Mikšík et al. 2025), Slovenia (Piltaver 1997, 2009; Jurc et al. 2004; Logar 2012; Mikšík et al. 2025; GP – this study), Spain (Vázquez Pardo et al. 2004), Switzerland (Secretan 1833; Knapp 1935; Kern 1946; Walty 1944, 1947, 1957, 1965; Hotz 1963; Burkhard 1971; Senn-Irlet et al. 1997, 2007; Waldvogel et al. 2001; Hagara 2015; GP – this study), and Türkiye (Gürzoğlu et al. 2024). Based on The Global Fungal Red List Initiative, it also occurs in Poland (Kiyashko et al. 2024; Mikšík et al. 2025); however, to the best of our knowledge and aside from reports in mycological forums, there are no confirming scientific publications on the occurrence of *I. torosus* in this country. Apparently widespread throughout Europe but rare and localised. Northern, eastern and southern distribution limits are yet to be established.

##### Examined material.

Bulgaria • Pazardzhik province: ca 2 km NE of Yundola village, Rodopi Mts, approx. 42°05'00.5"N, 23°53'38.5"E, ca. 1399 m, numerous specimens, under *A. alba*, *P. abies* and *F. sylvatica*, 07 Jul 2024, K. Milushev, SOMF 30991; • ibid., 09 Jul 2024, B. Assyov & I. Penchev, SOMF 30963 & 30964 (GP); Croatia • Primorje-Gorski Kotar County: Parg near Čabar, 45°35'10.1"N, 14°37'14.8"E, 832 m, 27 Jul 2023, four specimens in all developmental stages, in a mixed forest under *F. sylvatica*, *A. alba*, *P. abies*, *Corylus avellana* and *Acer pseudoplatanus*, with *Vaccinium myrtillus* and other shrubs and plants, D. L. Zbašnik, V. Ferenčak & N. Ferenčak, CNF 10/249, duplicate MG1017 (GP, specimen intended for proposal as the conserved type); • ibid. (ca. 30 m distant), 10 Aug 2023, one middle-aged and one mature specimen, N. Ferenčak, CNF 10/324; • Skrad, 45°24'36.9"N, 14°54'50.2"E, 774 m, 15 Aug 2021, one mature specimen, in a mixed forest under *F. sylvatica*, *A. alba*, *P. abies* and *C. avellana*, N. Ferenčak, CNF 10/79 (GP); • ibid. (ca. 20 m distant), 10 Aug 2023, two mature specimens, N. Ferenčak, CNF 10/321; • ibid., one young and one middle-aged specimens, 10 Aug 2024, N. Ferenčak, CNF 10/323 (GP); Czechia • Zlín region: Nový Hrozenkov, Beskydy Mountains, ca. 49°21'06.3"N, 18°11'22.5"E, ca. 700 m, under *A. alba*, *F. sylvatica* and *C. avellana*, 10 Aug 2018, M. Mikšík, MMB35/2018; • ibid., Nový Hrozenkov, Beskydy Mountains, ca. 49°21'06.3"N, 18°11'22.5"E, ca. 700 m, a single young specimen, under *A. alba*, *F. sylvatica* and *C. avellana*, 9 Aug 2025, M. Mikšík, PRM965778 (GP); France • Auvergne-Rhône-Alpes: Savoie, unknown locality, 26 Jun 2017, M. Broussal, CEFE Montpellier n° FR2017509 (GP); Germany • Bavaria: Garmisch-Partenkirchen district, Krün OT Gerold, Buckelwiesen am Plattele, 47°29'29"N, 11°11'59"E, 900 m, under *P. abies* on calcareous soil, 07 Sep 2007, J. Schreiner, GS-47; • ibid., Buckelwiesen am Geißschädel, ca. 47°29'17.02"N, 11°14'50.51"E, 930 m, two young and one middle-aged specimens, under *P. abies* and *F. sylvatica*, 13 Sep 2007, J. Schreiner, JS 05/07 (GP); • Rhineland-Palatinate: Vulkaneifel, Gerolstein, Gees, ca. 50°12'54.0"N, 6°41'07.5"E, 455 m, under *P. abies* and *F. sylvatica* on calcareous soil, 27 Jul 2009, R. Wald, JS 01/09; • ibid., 50°12'54.0"N, 6°41'07.5"E, under *F. sylvatica* and *P. abies*, 27 Aug 2023, K. Wehr, KW-2023-08-27-1 (GP); • ibid., 50°12'57.0"N, 6°41'07.7"E, 445 m, under *F. sylvatica*, 27 Aug 2023, R. Wald and K. Wehr, KW-2023-08-27-2 (GP); • Vulkaneifel, Mürlenbach, Remmelbachtal, 50°09'20"N, 6°38'42"E, 445 m, under *F. sylvatica*, 27 Aug 2023, R. Wald and K. Wehr, KW-2023-08-27-3 (GP); Hungary • Heves County: Mátra Mts, Parádóhuta, Lake Pisztrángos-tó, under *F. sylvatica*, 29 Aug 2010, L. Albert, AL 10/144 (GP); Italy • Veneto: Belluno, Tai di Cadore, Fontana del Sass, 46°24'54.2"N, 12°20'48.7"E, 940 m, a single middle-aged specimen, in a mixed forest dominated by *P. abies* and *A. alba* with the presence of *F. sylvatica* on calcareous soil, 01 Jul 2022, L. Lorenzon, MG958 (GP); • Belluno, Danta di Cadore, along the margin of the peaty-moss of Val di Ciampo, 46°33'59.7"N, 12°29'45.6"E, 1381 m, one middle-aged and one mature specimen, in a mixed forest dominated by *P. abies* and *A. alba* with the sporadic presence of *F. sylvatica* on calcareous soil, 19 Jul 2024, L. Lorenzon, MG1020 (GP); • Belluno, Pieve di Cadore, Col Vacher, 46°24'45.6"N, 12°20'59.9"E, 1020 m, two mature specimens, in a mixed forest dominated by *P. abies* and *A. alba* with sporadic presence of *F. sylvatica* on calcareous soil, 14 Jul 2017, P. Raminelli, A. Boscolo and R.J. Ferrari, MG1033 (FRJ009-2017; GP); Romania • Suceava, Mănăstirea Humorului, 47°36'16.7"N, 25°49'01.4"E, 700 m, three specimens, under *F. sylvatica*, *P. abies* and *A. alba*, 22 Jul 2025, P. Nimigean, PN-2025-07-22 (GP); Slovenia • Črnomelj admin. unit: Stari trg ob Kolpi, 45°30'55.6"N, 15°04'41.4"E, 378 m, two young and one middle-aged specimens, in a mixed forest under *F. sylvatica* and *A. alba*, 03 Jul 2024, E. Zupan, CNF 10/320 (GP); Switzerland • Bern canton: Oberlangenegg, Limpach, 46°47'56.6"N, 7°45'25.0"E, 1081 m, three specimens, under *F. sylvatica*, 23 Sep 2022, S. Scheurer, ZT Myc 0094431 (GP); • Glarus canton: Glarus Nord, Näfels, Obersee, 47°5'26.8"N, 9°0'15.4"E, 1121 m, in a mixed forest under *F. sylvatica*, *A. alba* and *P. abies*, 18 Jul 2023, D. Schlegel, ZT Myc 0094438 (GP); • Neuchâtel canton: Les Cottards, 47°4'2.1"N, 6°44'49"E, 1070 m, habitat unknown, 10 Sep 2024, C.-H. Pochon, ZT Myc 0094439 (GP); • Schwyz canton: Schuebelbach, 47°08'32.3"N, 8°54'14.8"E, 722 m, two specimens, 19 Sep 2022, B. Ziltener, ZT Myc 0094434 (GP); • St. Gallen canton: St. Gallen, 47°23'59.4"N, 9°20'24.1"E, 620 m, six specimens, in a shadowy and humid forest near the confluence of two rivers on calcareous soil, under *A. alba* and *F. sylvatica*, 02 Aug 2015, M. Cartabia, MC20150802.2 (GP); • Zurich canton: Oberrieden, 47°16'23.4"N, 8°33'18.1"E, 539 m, a single specimen, under *F. sylvatica*, *Quercus* sp. and *Betula pendula*, 30 Sep 2020, J. Brännhage, ZT Myc 0094432 (GP); • Winterthur-Eschenberg, Hinterwald, 47°28'03.9"N, 8°45'01.6"E, 551 m, a single specimen, in a mixed forest under *F. sylvatica*, *A. alba*, *Quercus* sp. and *Pinus sylvestris*, 21 Jul 2019, R. De Marchi, ZT Myc 0094435 (GP).

#### Imperator occultus

Taxon classificationFungiBoletalesBoletaceae

Ferenčak, Požnjak, Gelardi, Guinb., Treindl, A. Gross, Saar, Cartabia, L. Albert, Mikšík, Zupan, Vizzini, Bellanger, Dima, Biketova, Spinaci & Fabrizi sp. nov.

AB90B0E9-0A4A-5F34-8B7B-DB1D25916EE7

Index Fungorum: IF906196

Figs 4, 5, 6, 8e, f

? – Boletus torosus var. *daniaudii* Fourré, Bulletin de la Société botanique du Centre-Ouest 33: 320. 2003 (“2002”) (nom. inval., Art. 39.1)
? – Boletus torosus var. *daniaudii* Fourré, Bulletin de la Société botanique du Centre-Ouest 34: 425. 2003 (nom. inval., Art. 39.1)
? – Boletus torosus var. *daniaudii* Fourré, Bulletin de la Société botanique du Centre-Ouest 35: 401. 2004 (nom. inval., Art. 39.1)

##### Etymology.

The epithet refers to occultism (Latin: *occultus* – hidden, secret, dissimulated in shadow), evoking the “cryptic” species merged into *I. torosus* till now.

##### Diagnosis.

Compared to the closely related *I. torosus*, the newly described species exhibits a whitish-cream then yellow-ochraceous to pale brown pileus, pores sometimes red to red-orange already in young specimens, larger pores at maturity (up to 2 mm in diameter), less prominent, more elongated and more regularly arranged reticulum on the stipe, shorter and slightly wider basidiospores with a different Q ratio and the occurrence in more thermophilic habitats at relatively low altitudes where it may associate with a broader range of host trees than *I. torosus*.

**Figure 4.**
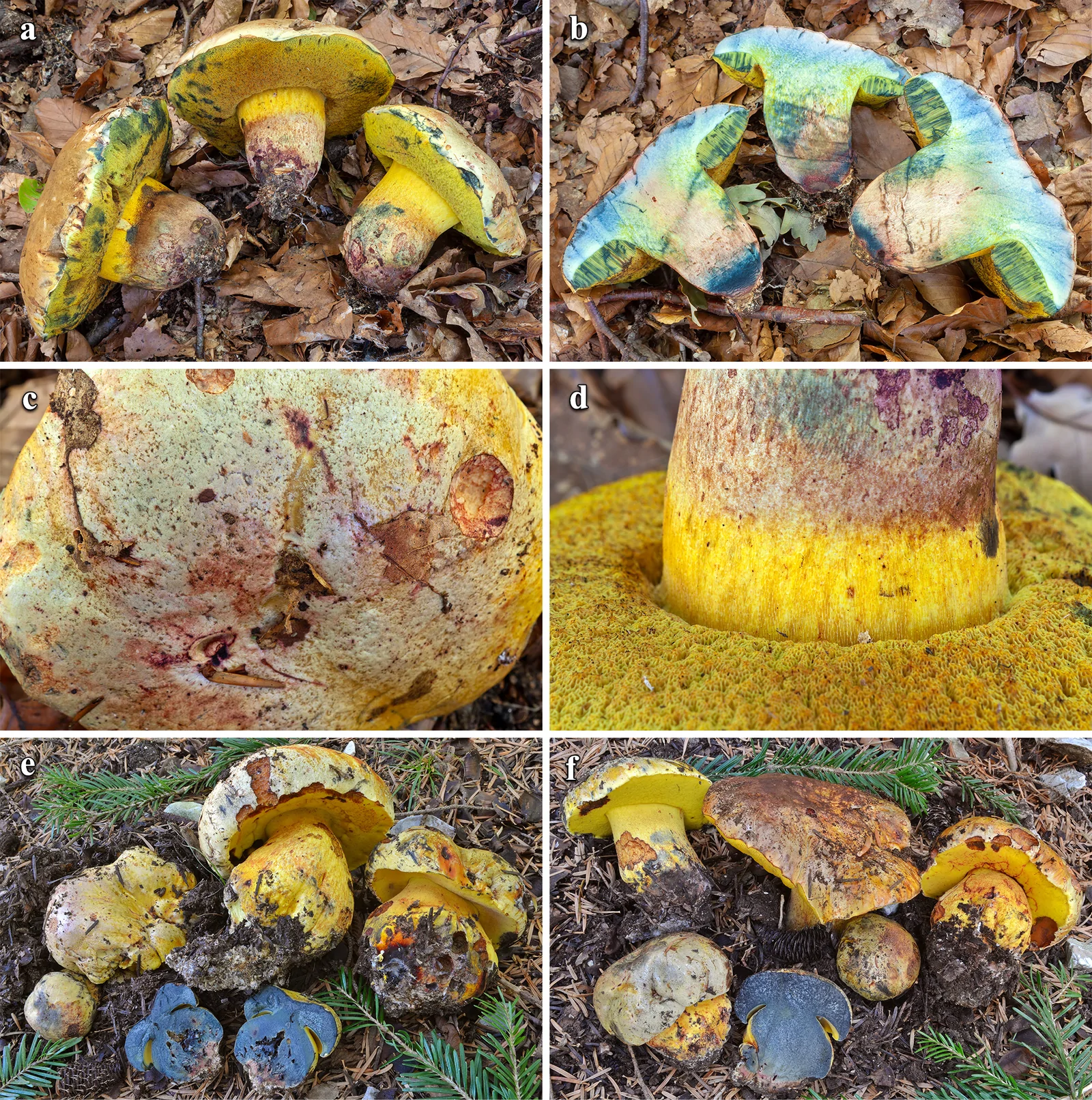
*Imperator occultus*, yellow-pored basidiomes in habitat. **a–d**. CNF 10/34, CROATIA, Varaždin County, near the village Podrute (holotype); **e**. MG1027; **f**. MG1026, ITALY, Marche, Fabriano (AN), Gola Rossa e di Frasassi Regional Natural Park, Mount Maltempo. Photos by N. Ferenčak (**a–d**) and M. Gelardi (**e, f**).

##### Holotype.

Croatia • Varaždin County: near the village of Podrute, in the vicinity of the town Novi Marof, 46°08'16.0"N, 16°15'52.3"E, 295 m, three specimens growing under *Quercus cerris*, *Q. petraea* and *Fagus sylvatica*, 7 Sep 2020, I. Valjak and N. Ferenčak, CNF 10/34 (MG1018, isotype); GenBank: ITS-LSU – PZ973662, *tef*1 – PZ973364, *rpb*2 – PZ973366.

##### Description.

***Ontogenetic development*** gymnocarpic. ***Basidiomes*** large, robust, dense and very heavy. ***Pileus*** (8.9) 9.7–21.0 cm broad, at first hemispherical then persistently convex and finally broadly flattened, often slightly depressed at center, regularly to hardly unevenly shaped by shallow depressions, decidedly fleshy, firm at the beginning but gradually softer with age; margin initially involute but straightening out until it is slightly curved downwards or completely plane to rarely uplifted especially in mature specimens, wavy-lobed from the early to middle stage of development and finally becoming even, cuticle not or only slightly extending beyond the tubes, cuticle not or only hardly separable from the pileus and tending to tear apart together with the context underneath; surface matt, dry but slightly greasy with moist weather, subtomentose-pruinose but soon smooth and glabrous, not or only occasionally rugulose-granulose in places in young specimens, not cracked but in case of severe drought rarely with shallow fine cracks around the center; variable in colour, evenly pale yellow (4A5) or yellow (4A6), bright yellow (4A7) or golden yellow (4B8) to warm yellow-ochraceous (4C8) at the very beginning but typically covered with a pruina ranging from whitish-creamy (4C1), very pale beige-grayish (4C2) or pale gray-olivaceous (4D3) to light grayish (4D2) (the background yellow colour is more intense towards the margin and in places where the pruina is absent), becoming copper orange (5C5) to pale copper brown (5D5) with scattered reddish-purple (10C8–10E8) zones that sometimes can spread and cover most of the surface with age; aged specimens have dominant ochre-brown (5D6) to copper brown (5D7) tints but with yellowish brown (5B4) to less frequently yellow (4B5) areas retained in some places; instantly turning blue (20F5) to dark blue (20F8) on handling, especially in young specimens and after a few hours fading to copper orange (7C6) or copper red (7D6) to sordid brownish-olive (5D3); nearly unchangeable in older specimens with dry weather; subcuticular layer white (2B1) to yellowish (2B6). ***Tubes*** at first extremely short then increasingly longer with age but shorter than the thickness of the pileus context (1.1–2.3 cm long), adnate to adnexed but soon depressed around the stipe apex, golden yellow (4C8) when young, greenish yellow (3C8) to olive-green (3D8) with age, immediately turning dark blue (20F8) when cut and later fading to copper orange (7C6) or copper red (7D6); easily to moderately hardly detachable from the pileal context above. ***Pores*** initially concave then forming a flat surface or only slightly convex-ascending, at first very small, then gradually wider with age (up to 2 mm in diam.), simple, roundish, becoming barely angular and very slightly elongated at maturity, bright yellow (4A7) or golden yellow (4B8) to sometimes orange (7B8) or reddish (9D7) from the early stage of development, in all colour variants (yellow, orange and red) pores are usually concolourous with the reticulum on the stipe; when pores yellow sometimes developing dispersed orange-red (7C6) to rusty brown (7D6) stains or irregular patches at the orifice; turning dark blue (20F8) to very dark blue (19F8) or nearly black (18F8) quickly on bruising or when injured, later fading to copper orange (7C6), copper red (7D6) or rusty brown (7E7). ***Stipe*** (6.3) 6.6–10.0 (12.2) × (2.9) 3.5–6.7 (7.1) cm; dry, stout, usually shorter than or as long as the pileus diameter, central to slightly off-center, solid, firm, dry, straight or faintly curved, at first ventricose-ovoid to ventricose-fusiform, then cylindrical, equal to sometimes tapering or swollen towards the base to decidedly clavate, occasionally bulbose, very rarely restricted in the middle part, not to moderately rooting; ground colour bright yellow (3B8) to golden yellow (4C8) throughout in young specimens then fading beige (4C3) except the apical part which remains of a bright yellow (3B8), sometimes in the lower part with reddish-purple (10F6) shades which, depending on age and weather conditions, tend to spread up to the middle part; in aged specimens watery pale brown (5C4); reticulum present, not prominent but well developed on the upper half or upper three quarters of the stipe, although in rare cases it can cover the stipe lengthwise down to the very base; meshes small to medium-sized, roundish to barely angular and regularly arranged, with a tendency, especially toward the stipe base and in older basidiomes, to become slightly elongated into ovoid meshes that may become progressively more open and partly evanescent, while remaining concolourous with the ground and only moderately raised; in all cases instantly cyanescent (20F8) when pressed then reddening (7C6) a long time after cutting; ***basal mycelium*** and ***rhizomorphs*** whitish beige (4C3) to cream (3C1); mycelium tightly attached to the stipe. ***Context*** very firm and tough when young, later soft textured and eventually flabby in the pileus (up to 3.6 cm thick in the central zone, up to 2.1 cm thick halfway to margin and gradually becoming thinner towards the edge), a little more fibrous in the stipe; evenly yellow (3C4) to yellowish white (3C2), whitish (3B1) in old specimens but usually with reddish purple (10F6) areas in the stipe base which tend to spread all over the stipe and sometimes also in the pileus in aged specimens, brownish (7E5) at the extreme base, instantly and violently turning Prussian blue (20F8) to dark blue (19F8) when exposed to air, more intensely in young and moist specimens, then becoming grayish (18E3) and after some hours reddish purple (10F6) to dark copper red (7D6); the cyanescence is less intense in aged specimens and generally localised in the pileus and lower half of the stipe, while the middle part does not oxidate or nearly so and remains yellowish white (3C2); beige pinkish (10C3), pinkish (10D4) to copper orange (7C6) in places eaten by slugs in the pileus and yellow-orange (6A4), copper orange (7C6) to purplish (10D4) in those of the stipe; context pinkish (11B5) to reddish purple (11E5) where eroded by maggots; subhymenophoral layer yellow (3C4); in exsiccate brown (6D8) to reddish brown (8E8) or dark brown (7F7) throughout. ***Odour*** initially pleasant but weak, mushroomy to fruity immediately after cutting but becoming unpleasant with age, of expired medicines, pharmaceutical lab or iodoform-like, a few minutes after cutting recalling the smell of dried porcini mushrooms. ***Taste*** mild, slightly sour or acidic. ***Spore print*** yellowish brown (5E8). ***Macrochemical spot-test reactions***: 20% and 30% KOH: staining light orange to orange (on grayish-olivaceous tint) to vinaceous red (on copper red tint) on pileus, bright orange on context and stipe but sordid brownish at base, orange to vinaceous red on hymenophore; Melzer: staining blue on context and hymenophore, light blue to blue on stipe, nil to light blue on pileus; Baral: staining blue on context, light blue to dark blue on hymenophore, nil to blue on pileus, nil on stipe; Lugol: staining blue to dark blue on context, light blue to blue on hymenophore, nil to blue on pileus, nil on stipe. Guaiac: staining light blue to blue (green) on context, light blue to dark blue on hymenophore, nil to dark-greenish blue on pileus, blue on stipe; Aniline: staining light orange or nil on context, nil on pileus and hymenophore, stipe surface not tested; Phenol: staining blue on pileus (brighter in moist areas, no reaction to light grey in 5 min, dark red in 15 min on drying), greenish blue on context (but unclear due to context oxidation) but red purple in 5 min, slowly blue on hymenophore, blue on stipe but red-purple-brown in 5–10 min; FeSO_4_: pale olivaceous to greenish blue or no reaction on pileus, nil to yellow-green then sordid olivaceous green on context (the cyanescence of context disappears), nil or sordid olive green to olive brown on hymenophore and stipe; 25% NH_3_: dirty yellowish (on grayish-olivaceous tint), light orange, orange to vinaceous red without extended halo (on copper red tint) on pileus, orange reddish or reddish brown to brown or rusty brown (1 min) on hymenophore, yellowish ochraceous to yellowish orange or light orange (with extended halo) or nil on context (the cyanescence of context disappears), orange reddish to reddish brown on stipe.

**Figure 5.**
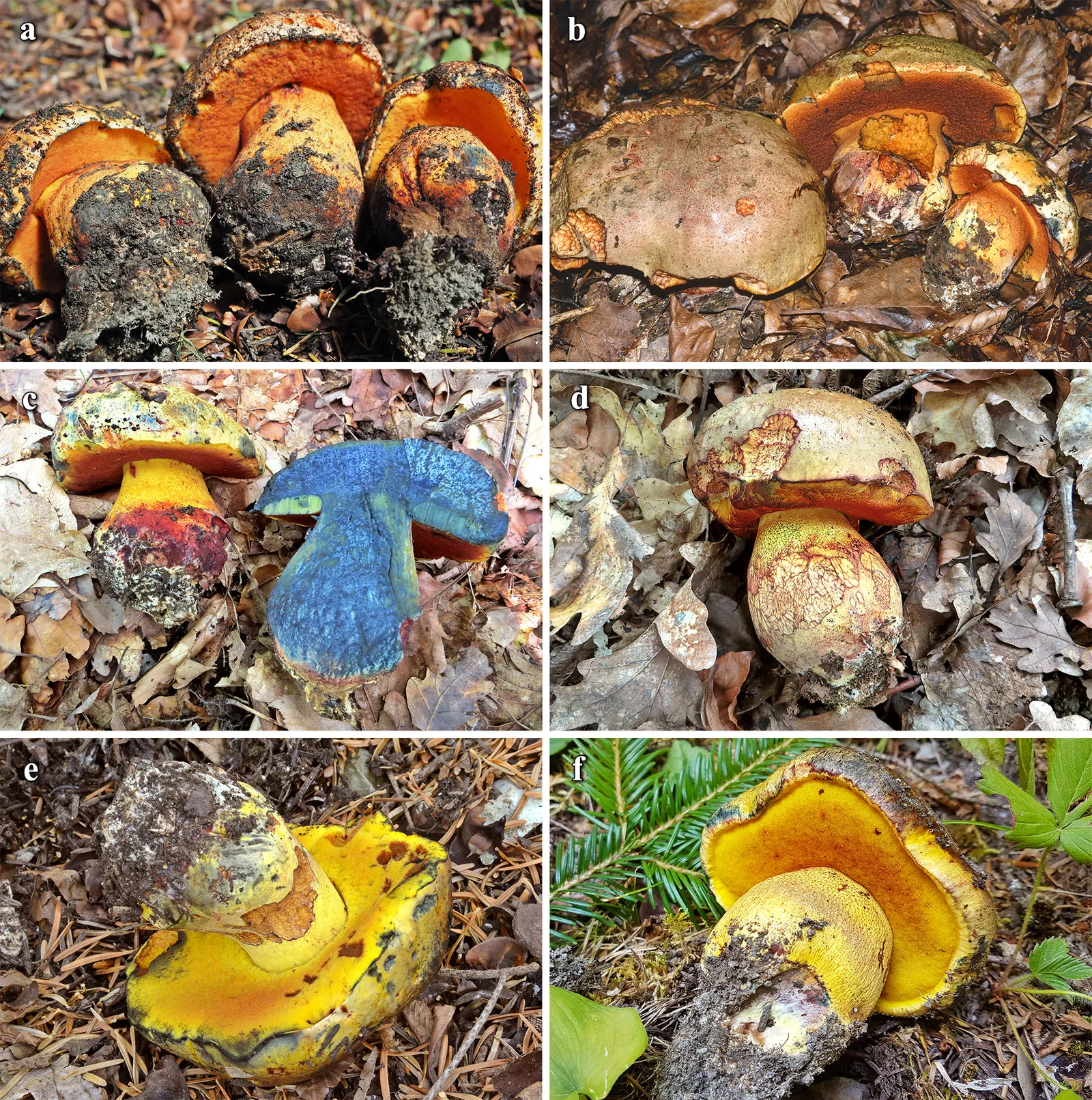
*Imperator occultus*, orange to red-pored basidiomes in habitat. **a**. JG2108011, FRANCE, Provence-Alpes-Côte d’Azur, Hautes-Alpes, Crots, Forêt domaniale de Boscodon-Bragousse, Clos-Joubert; **b**. GS-2018-12, GERMANY, Baden-Württemberg, Bollschweil; **c**. K-M001450423, CROATIA, City of Zagreb, between Planina Donja and Kašina; **d**. K-M001450420, CROATIA, City of Zagreb, between Planina Donja and Kašina; **e**. MG1028, ITALY, Marche, Fabriano (AN), Gola Rossa e di Frasassi Regional Natural Park, Mount Maltempo; **f**. JG2207251, FRANCE, Provence-Alpes-Côte d’Azur, Hautes-Alpes, Crots, Forêt domaniale de Boscodon-Bragousse, Clos-Joubert. Photos by J. Guinberteau (**a, f**), G. Saar (**b**), D. Šikić (**c, d**) and M. Gelardi (**e**).

**Figure 6.**
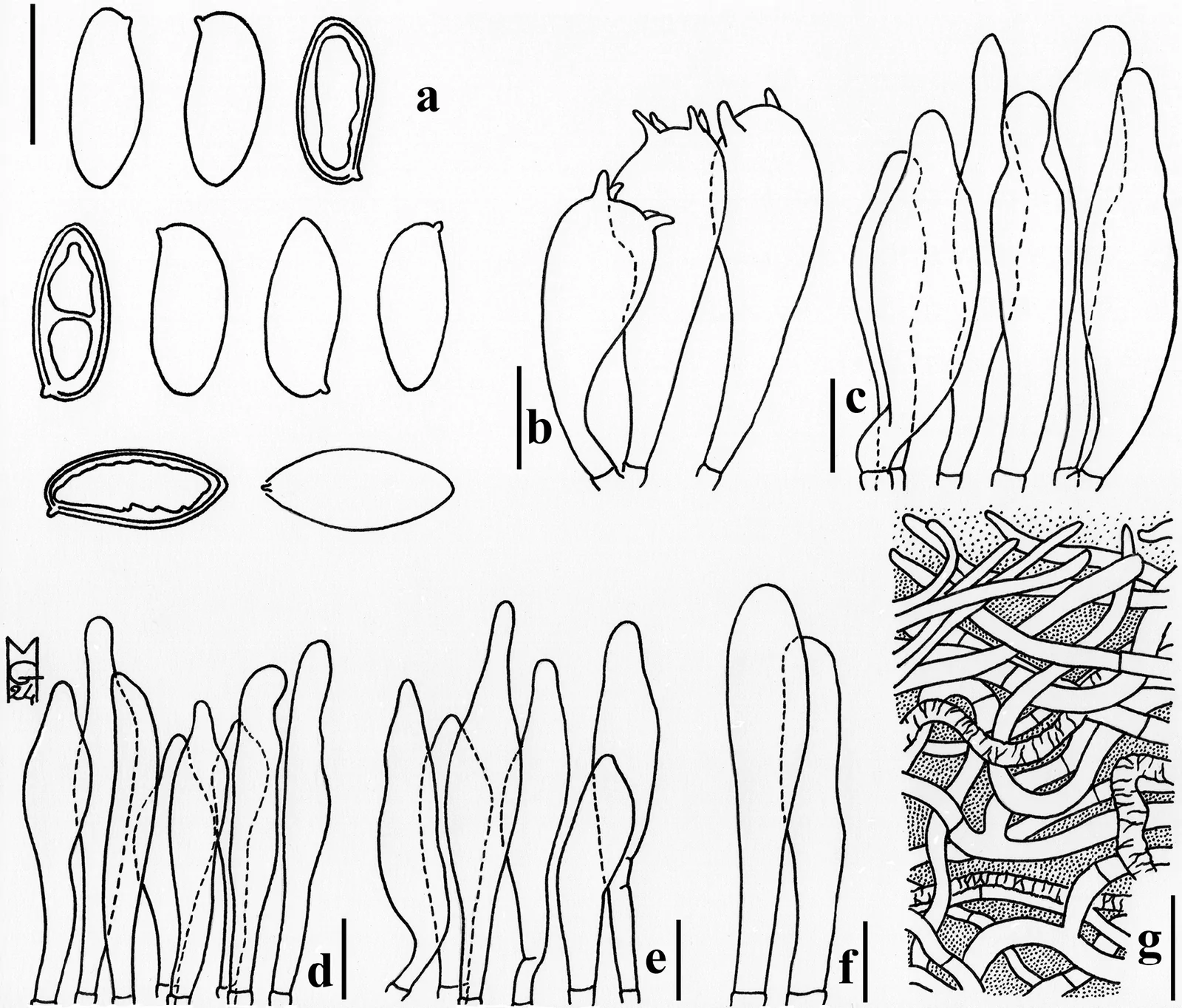
Microscopic features of *Imperator occultus* (CNF 10/34, holotype); **a**. Basidiospores; **b**. Basidia; **c**. Caulocystidia; **d**. Cheilocystidia; **e**. Pleurocystidia; **f**. Pseudocystidia; **g**. Pileipellis. Scale bars: 10 µm (**a–f**); 20 µm (**g**). Drawings by M. Gelardi.

***Basidiospores*** [630/20/14] (9.7) 13.5 ± 1.0 (16.0) × (4.9) 5.9 ± 0.3 (6.9) μm, Q = (1.82) 1.86–2.67 (2.70), Qm = 2.29 ± 0.15, V = 247 ± 39 μm^3^ (a single anomalous spore measured 16.7 × 7.5 µm!), fairly uniform in dimension, inequilateral, ellipsoid to ellipsoid-fusiform in both side and face view, smooth, apex rounded, with a short apiculus and with a suprahilar applanation or more rarely a shallow depression and with an adaxial swelling, moderately thin-walled (0.3–0.5 μm), golden yellow coloured in water and 5% KOH, having one or two large oil droplets when mature, rarely pluri-guttulate, inamyloid, cyanophilic and with a very faint metachromatic reaction. ***Basidia*** (23) 27–45 × 9–15 μm (n = 45), cylindrical-clavate to clavate or broadly clavate, moderately thick-walled (0.3–1.0 μm), predominantly 4-spored but also 1- or 2-spored, usually bearing short sterigmata (2–4 μm, exceptionally up to 6–7 μm long), hyaline to pale yellowish and containing straw-yellow oil guttules in water and 5% KOH, bright yellow (inamyloid) in Melzer’s, without basal clamps; basidioles subcylindrical or cylindrical-clavate to faintly clavate, similar in size to basidia. ***Cheilocystidia*** (23) 26–48 × 4–10 μm (n = 34), very common, moderately slender, projecting straight to sometimes flexuous and protruding from the hymenial palisade, irregularly cylindrical or cylindrical-fusiform to fusiform, rarely shortly mucronate, sublageniform to subclavate or sphaeropedunculate, with rounded to subacute tip, smooth, moderately thick-walled (0.3–1.0 μm), hyaline to pale yellowish in water and 5% KOH, bright yellow (inamyloid) in Melzer’s, without epiparietal encrustations. ***Pleurocystidia*** (20) 26–58 (69) × 6–11 μm (n = 21), uncommon and dispersed, shape, colour and chemical reactions like but slightly longer than cheilocystidia. ***Pseudocystidia*** not uncommon although scattered, intermixed with ordinary hymenial cystidia but originating deeper in the trama, emerging from the lateral strata or even close to the mediostratum; irregularly cylindrical to subclavate and with a uniform bright honey yellow plasmatic pigment in water and 5% KOH, inamyloid in Melzer and with an orthochromatic reaction, usually slightly longer than hymenial cystidia, (35) 39–55 (63) × 6–11 μm (n = 8). ***Pileipellis*** an ixotrichoderm soon tending to ixocutis consisting of loosely interwoven to subparallel, elongated, filamentous and sinuous, frequently branched hyphae embedded in gelatinous matter; terminal elements (29) 50–127 (150) × (2) 3–7 μm, long and slender, cylindrical, apex rounded-obtuse, enlarged or sometimes pointed, thin-walled (up to 0.3 μm), hyaline to pale yellowish in water and 5% KOH, inamyloid in Melzer’s, smooth to less frequently ornamented by a subtle granular or zebra-like, dark brown to blackish epiparietal encrustation which tends to dissolve in KOH; subterminal elements similar in shape, size and colour to terminal elements. ***Stipitipellis*** a layer of slender, parallel to subparallel and longitudinally running, smooth-walled, adpressed hyphae, 2–10 μm wide, hyaline to very pale yellowish in water and 5% KOH; the stipe apex is covered by a well-developed caulohymenial layer consisting of sterile caulobasidioles, very frequent, predominantly 1- or 2-spored, fertile caulobasidia, 28–49 (51) × (7) 9–16 μm, sterigmata 2–3 μm long (n = 13) and abundant projecting cylindrical-fusiform to fusiform or sublageniform to sphaeropedunculate ***caulocystidia*** similar in size and colour to hymenial cystidia, (26) 28–49 (51) × 6–13 μm (n = 22), having a wall up to 0.5 μm thick. ***Lateral stipe stratum*** under the caulohymenium present and well differentiated from the stipe trama, of the “boletoid type”, at the stipe apex a (25) 30–50 (60) μm thick layer consisting of divergent, inclined and running towards the external surface, loosely intermingled and branched hyphae remaining separate and embedded in a gelatinous substance. ***Stipe trama*** composed of irregularly and densely arranged, strongly interwoven, filamentous, smooth, inamyloid hyphae, 3-16 μm broad. ***Hymenophoral trama*** bilateral divergent of the “*Boletus*-type”, with slightly to strongly divergent, recurved-arcuate and loosely arranged hyphae, lateral stratum hyphae diverging at an angle of 45°–60° to the mediostratum, very frequently branched, distantly septate and generally not restricted at septa, gelatinous (lateral strata hyphae in transversal section not touching each other, (3) 4–8 (9) μm apart, 2–8 μm broad), hyaline to very pale yellowish in water and 5% KOH, inamyloid in Melzer’s; lateral strata (10) 20–50 (60) μm thick, mediostratum (10) 20–50 (60) µm thick, axially arranged, consisting of a tightly adpressed, non-gelatinous bundle of hyphae, 2–8 µm broad, distantly septate; in Congo Red the mediostratum is darker than the lateral strata. ***Thromboplerous hyphae*** very scattered but more common in the pileipellis and in the stipe base trama, thick-walled, rarely septate, melanized, with a dense golden yellow homogeneous content in 5% KOH. ***Clamp connections*** absent in all tissues.

##### Edibility.

Unknown. Not recommended for consumption (see notes above under *I. torosus*).

##### Ecology and phenology.

Solitary to gregarious, growing on calcareous soil (soil pH 6.0–6.5) among litter, mainly under broadleaved trees at low altitudes, preferably with members of the family *Fagaceae*, namely *Quercus* (*Q. cerris*, *Q. ilex*, *Q. petraea*, *Q. pubescens*, *Q. robur*, etc.) and *F. sylvatica*, but in southern Europe occurring also in pedemontane and montane areas in pure stands of natural and planted conifers of the *Pinaceae*, such as *Abies* (*A. alba* and *A. cephalonica*) up to 1300 m a.s.l. Probably also under *C. avellana* (*Betulaceae*). Occurring in summer (July to September).

##### Known distribution.

Croatia (GP – this study), France (GP – this study), Germany (GP – this study), Italy (GP – this study), Slovenia (GP – this study), Spain (GP – this study) and Switzerland (GP – this study). Apparently widespread throughout the mainland Mediterranean basin but rare and localized. Northern, eastern and southern distribution limits are yet to be established.

##### Examined material.

Croatia • Varaždin County: near the village of Podrute, in the vicinity of the town of Novi Marof, 46°08'16.0"N, 16°15'52.3"E, 295 m, three specimens, under *Q. cerris*, *Q. petraea* and *F. sylvatica*, 07 Sep 2020, I. Valjak and N. Ferenčak, CNF 10/34 (holotype; GP), MG1018 (isotype); • ibid., two specimens, 22 Aug 2021, I. Valjak and N. Ferenčak, CNF 10/131 (GP); • Klenovnik, 46°16'00.9"N, 16°02'55.6"E, 315 m, one specimen, under *F. sylvatica*, *Q. petraea*, *Carpinus betulus* and *Tilia* sp., 10 Sep 2020, Z. Lončar and N. Ferenčak, CNF 10/132 (GP); • City of Zagreb: between Planina Donja and Kašina, 45°55'33.6"N, 16°06'16.4"E, 310 m, in mixed forest of *F. sylvatica* and *Q. petraea*, a single young specimen, 17 Jul 2018, D. Šikić, K-M001450423 (AB B18-290; GP); • ibid., in a mixed forest under *F. sylvatica* with the presence of *Q. petraea*, a single young specimen, 26 Jul 2018, D. Šikić, K-M001450422 (AB B18-291; GP); • ibid., in a mixed forest under *F. sylvatica* with the presence of *Q. petraea*, two specimens, 26 Jul 2018, D. Šikić, K-M001450420 (AB B18-292; GP); • ibid., one specimen, under *F. sylvatica*, *Q. petraea*, *Q. pubescens* and *P. sylvestris*, 18 Aug 2023, N. Ferenčak, CNF 10/247 (GP); • ibid. (up to 30 m distant), one specimen, 16 Jul 2023, N. Ferenčak, CNF 10/264; • ibid., one specimen, 14 Jul 2024, N. Ferenčak, CNF 10/319 (GP); • Zagreb County: Staničići Žumberački, 45°44'00.9"N, 15°30'31.9"E, 476 m, two specimens, under *F. sylvatica*, *Q. pubescens*, *Q. petraea*, *Prunus avium* and *A. pseudoplatanus*, 11 Aug 2024, D. Balaško and N. Ferenčak, CNF 10/325 (GP); • ibid., one specimen, under *F. sylvatica*, *Q. petraea*, *Q. pubescens* and *A. pseudoplatanus*, 01 Aug 2025, N. Ferenčak, CNF 10/335; • Željezno Žumberačko, 45°44'35.9"N, 15°27'55.0"E, 475 m, two specimens, under *F. sylvatica* and *A. pseudoplatanus*, 11 Aug 2024, N. Ferenčak and D. Mihalec, CNF 10/326 (GP); France • Ile-de-France: Essonne, Bouville, 15 Aug 2023, under *Quercus* sp. on calcareous soil, R. Chalange, FR2024497 (PAM23081501; GP); • Provence-Alpes-Côte d’Azur: Hautes-Alpes, Crots, Forêt domaniale de Boscodon-Bragousse, Clos-Joubert, 44°29'17.11"N, 6°27'56.40"E, 1309 m, 16 Aug 2015, four young specimens, under *A. alba*, J. Guinberteau, JG1508161; • ibid., 07 Sep 2017, two mature specimens, under *A. alba*, J. Guinberteau, JG1709071; • ibid., 13 Aug 2018, three specimens, under *A. alba*, J. Guinberteau, JG1808131; • ibid., 16 Aug 2018, three specimens, under *A. alba*, J. Guinberteau, JG1808161; • ibid., 21 Aug 2018, five specimens, under *A. alba*, J. Guinberteau, JG1808211; • ibid., 01 Aug 2021, three young and three middle-aged specimens, under *A. alba*, J. Guinberteau, JG2108011 (GP); • ibid., 25 Jul 2022, a single young specimen, under *A. alba*, J. Guinberteau, JG2207251; • ibid., 19 Aug 2022, five middle-aged and four old specimens, under *A. alba*, J. Guinberteau, JG2208191; • ibid., 04 Sep 2023, three old specimens, under *A. alba*, J. Guinberteau, JG2309041; • ibid., 03 Aug 2025, three specimens, under *A. alba*, J. Guinberteau, JG2508031; Germany • Baden-Württemberg: Ebringen, 47°57'25.7"N, 7°47'44.5"E, 460 m, 20 Aug 2006, one young and one middle-aged specimen, in a mixed forest under *F. sylvatica*, G. Saar, GS-2018-13 (GP); • Bollschweil, 47°55'58.5"N, 7°46'31.1"E, 401 m, 27 Aug 2002, two young and four middle-aged specimens, in a mixed forest under old *Q. petraea* and young *F. sylvatica*, G. Saar, GS-2018-12 (GP); • ibid., 47°55'58.3"N, 7°46'30.1"E, 350 m, 19 Sep 2025, a single young specimen, in a mixed forest under *Abies* sp. and *Quercus* sp., GS-25-15 (GP); • Huttingen, 47°40'10.4"N, 7°32'02.3"E, 310 m, two young specimens, in a mixed forest under *Fagus* sp. and *Quercus* sp., 19 Sep 2025, GS-25-16 (GP); Italy • Emilia-Romagna: Piacenza, Farini, 44°43'36.4"N, 9°31'17.3"E, 1050 m, six specimens in all developmental stages, in a mixed woodland under *A. alba*, *F. sylvatica*, *P. abies* and *P. sylvestris*, 18 Aug 2009, E. Pini, PRM965782 (GP); • Parma, Varano de’ Melegari, next to Scarampi, 44°42'48.4"N, 9°58'15.7"E, one young and two middle-aged specimens, under *Q. pubescens* with *Rosaceae* spp., 18 Sep 2025, A. Barbieri, PRM965781 (GP); • Lombardy, Varese, Cascina Tagliata, 45°51'09.3"N, 8°48'33.2"E, 534 m, three mature specimens, under *Q. pubescens*, *A. campestris* and *Tilia* sp., 19 Sep 2023, M. Cartabia, MC20230919.1 (GP); • Marche: Ancona, Fabriano, Gola Rossa e di Frasassi Regional Natural Park, Mount Maltempo, 43°20'47.5"N, 13°01'48.4"E, 1021 m, several specimens in all developmental stages, along a trackside and inside an artificial plantation of *A. cephalonica* with sporadic presence of *F. sylvatica* on calcareous soil, 07 Sep 2024, G. Spinaci, M. Gelardi, F. Costanzo and O. Gelardi, MG1026 (GP); • ibid., 100 m away from the previous collection, several specimens in all developmental stages, inside an artificial plantation of *A. cephalonica* with sporadic presence of *F. sylvatica* on calcareous soil, 07 Sep 2024, G. Spinaci, M. Gelardi, F. Costanzo and O. Gelardi, MG1027 (GP); • ibid., a single middle-aged specimen with orange pores, inside an artificial plantation of *A. cephalonica* with sporadic presence of *F. sylvatica* on calcareous soil, 07 Sep 2024, G. Spinaci, M. Gelardi, F. Costanzo and O. Gelardi, MG1028 (GP); • Trentino-Alto Adige: Trento, Ravina di Trento, unknown site, ca. 300 m, two middle-aged specimens, in hilly environment in a mixed broadleaved woodland under *Q. pubescens*, *C. betulus* and *Corylus avellana*, 27 Jul 2009, P. Ceccon, MG216; • Bolzano, Sella, Termeno along Strada del Vino between Castelvecchio and Caldaro, 46°21'20.1"N, 11°14'13.2"E, 490 m, five specimens, in a pure stand of *F. sylvatica* on calcareous soil, 14 Aug 2016, C. Rossi, CR-14-08-2016 (GP); • ibid., one young and two middle-aged specimens, with *F. sylvatica* on calcareous soil, 18 Jul 2017, N. Oppicelli, A. Fellin, R.J. Ferrari and T. Cester, MG1034-1 (FRJ010-2017; GP) and MG1034-2 (GP); • ibid., three middle-aged specimens, with *F. sylvatica* on calcareous soil, 14 Sep 2018, 46°21'18.4"N, 11°14'11.8"E, 500 m, R.J. Ferrari, MG1035 (FRJ106-2018; GP); • Trento, Volano, Volano Buse, 45°54'24.6"N, 11°04'13.6"E, 553 m, two middle-aged and two mature specimens, under *Q. pubescens* and *C. betulus*, 18 Sep 2023, A. Valdagni, PRM965780 (GP); Slovenia • Kočevje admin. unit: near the village of Podlesje, 45°30'10.8"N, 14°59'31.8"E, 538 m, one mature specimen, under *F. sylvatica*, *Q. cerris*, *Q. petraea*, *C. betulus* and *Fraxinus* sp., 13 Sept 2024, R. Šuster and E. Zupan, LJF8893 (GP); Spain • Catalonia: Girona, Font-Rubí, next to Col de la Boixeda, 42°19'41.3"N, 2°25'46.9"E, 1105 m, two middle-aged and one mature specimen, under *F. sylvatica* and *A. alba*, 11 Sep 2022, L. Sanchez, PRM965779 (GP); Switzerland • Basel-Land canton: Bubendorf, 47°25'47.2"N, 7°43'56.4"E, 451 m, three specimens, in a mixed forest under *F. sylvatica*, *A. alba* and *P. abies*, 18 Sep 2023, A. Treindl and J. Haverbeck, ZT Myc 0094430 (GP); • ibid., 47°25'47.2"N, 7°43'56.4"E, 451 m, in a mixed forest under *F. sylvatica*, *A. alba* and *P. abies*, 9 Sep 2022, J. Haverbeck, ZT Myc 0094437 (GP); • Bubendorf, Furlenbodenweg, Landschachen, 47°27'34.1"N, 7°45'01.0"E, 410 m, under *F. sylvatica* on calcareous soil, 1 Sep 2018, leg. M. Danz, J. Schreiner and H. Schreiner, det. J. Haverbeck, JS 02/18 (GP); • Niederdorf, Gemeindematt, Eichbüchel, 47°25'48.6"N, 7°43'53.5"E, 430 m, under *A. alba*, *P. abies*, *P. menziesii*, 01 Sep 2018, leg. M. Danz, J. Schreiner and H. Schreiner, det. J. Haverbeck, JS 03/18 (GP).

##### Additional examined material.

*Imperator rhodopurpureus* f. *rhodopurpureus*; Croatia • City of Zagreb: Planina Donja, 45°55'34.0"N, 16°06'17.0"E, 312 m, one mature specimen, under *F. sylvatica*, *Q. petraea*, *Q. pubescens* and *P. sylvestris*, 03 Aug 2023, N. Ferenčak, CNF 10/322 (GP).

*I. rhodopurpureus* f. xanthopurpureus; • Varaždin County: near the village of Gačice, in the vicinity of the town of Ivanec, 46°14'27.9"N, 16°12'09.9"E, 226 m, seven specimens in all developmental stages, in a mixed broadleaved woodland under *F. sylvatica*, *Q. petraea*, *Q. cerris* and *C. betulus*, 11 Aug 2019, N. Ferenčak, CNF 10/53 (GP); • ibid., three mature specimens, 21 Aug 2020, N. Ferenčak, CNF 10/135 (GP); • ibid., one specimen, 7 Aug 2023, N. Ferenčak, CNF 10/287; • Gačice, 46°14'03.6"N, 16°12'54.9"E, 254 m, one mature specimen, under *F. sylvatica* and *Q. petraea*, 29 Aug 2019, N. Ferenčak, CNF 10/12 (GP); • Osečka, in the vicinity of the town of Ivanec, 46°13'03.4"N, 16°14'05.5"E, 212 m, one mature specimen, in a mixed broadleaved woodland under *F. sylvatica*, *Q. petraea*, *Q. cerris* and *C. betulus*, 30 Jun 2024, N. Ferenčak, CNF 10/331; France • Bourgogne-Franche-Comté: Côte-d’Or, Tarsul, under *Quercus* sp. and *C. betulus* on calcareous soil, 29 Aug 2019, P. Cercley, FR2020646 (PAM19090002, as “*Boletus xanthocyaneus*”) (GP); • ibid., 25 Aug 2023, P. Cercley, FR2024494 (as “*Boletus xanthocyaneus*”) (GP); • Ile-de-France: Seine-et-Marne, Bombon, three specimens, under *Quercus* sp. and *C. betulus* on clay-calcareous soil, 6 Sep 2023, R. Chalange, FR2024495 (as “*Boletus xanthopurpureus*”) (GP); • ibid., Fontainebleau, gorges de Franchard, 48°24'34.8"N, 2°38'24.1"E, several specimens, under *Quercus* sp., Oct 2022, P. Le Pogam-Alluard, FR2024473 (GP); Italy • Friuli-Venezia Giulia: Udine, Carlino, Bosco Sacile (relict of the ancient Padana forest), 45°47'37.7"N, 13°11'5.8"E, 5 m, one young and one middle-aged specimen, under *Q. robur* and *C. betulus* with the presence of *Fraxinus angustifolia* and *Populus tremula* on clayey-calcareous soil, 15 Sep 2020, A. Boemo, MG1038 (ALV40448, sample 1) (GP); • ibid., six young to middle-aged specimens, under *Q. robur* and *C. betulus* with the presence of *F. angustifolia* and *P. tremula* on clayey-calcareous soil, 11 Aug 2021, A. Boemo, MG1039 (GP); Slovenia • Koper admin. unit: Gračišče/Butari, 45°29'10.3"N, 13°51'51.5"E, 315 m, two middle-aged specimens, under *Q. pubescens*, *F. sylvatica*, *P. sylvestris* and *C. orientalis*, 09 Sep 2025, E. Zupan, LJF9368 (GP).

*Imperator luteocupreus*; Switzerland • Ticino canton: Lugano, Cadro, Posmò, 46°02'47.8"N, 8°59'48.6"E, 699 m, two specimens, under *F. sylvatica*, 26 Sep 2020, R. De Marchi, ZT Myc 0094436 (GP).

*Cyanoboletus poikilochromus*; Croatia • Šibenik-Knin County: Brištani, 43°51'56.3"N, 15°58'42.4"E, 58 m, two mature specimens, under *Q. pubescens*, *Q. ilex* and *Carpinus orientalis*, 24 Oct 2023, N. Ferenčak, CNF 10/301 (GP).

## Discussion

### Early recognition of two different taxa

The plurality within “torosus-like” collections was already signposted in the pre-molecular era field literature as an ecologically structured bifurcation. Estadès and Deïana (2004) documented two different populations of *B. torosus*, one restricted to the uplands of the montane areas of south-eastern France and western Switzerland (Alps, Jura) in association with *F. sylvatica* and conifers (*Abies*, *Picea*) on calcareous soil, the other occurring in the lowland of western and northern France in association with various broadleaved trees with a preference for *Quercus* species. In this regard, they clearly stated in the footnotes: “The lowland collections of *B. torosus*, associated with *Quercus* (or *Quercus*-*Fagus*), in western France (Deux-Sèvres, Charente-Maritime), as well as in the Paris region (Essonne), have a slightly different appearance and colour. These collections, rather localised and sometimes abundant, appear paler in colour, with slightly less livid grey, grey, and red on the pileus with age, with less orange-yellow pores and stipe, the stipe is on average less stout and finally the spores are very slightly shorter. These collections are undoubtedly much more difficult to identify than those in our regions” (“Les récoltes planitiaires de *B. torosus*, liées aux *Quercus* (ou *Quercus*-*Fagus*), dans l’ouest de la France (Deux-Sèvres, Charente-Maritime), ainsi que dans la région parisienne (Essonne), ont un faciès et des couleurs un peu différentes; ces récoltes, assez localisées et parfois abondantes, semblent plus claires, avec un peu moins de gris livide, de gris et de rouge sur le chapeau avec l’âge, avec des pores et un stipe moins jaune orangé, le stipe est en moyenne moins trapu et enfin les spores sont très légèrement plus courtes, récoltes qui sont sans doute beaucoup plus difficiles à déterminer que dans nos régions”; Estadès and Deïana 2004: 26), and also: “The spores of lowland collections under *Quercus*, which we have examined, are slightly smaller on average” (“Les spores des récoltes de plaine sous *Quercus*, que nous avons examinées, sont, en moyenne, un peu plus petites”; Estadès and Deïana 2004: 23). Interestingly, G. Redeuilh also seemed to believe in the existence of two different taxa, as emerged during various conversations with G. Fourré: “He based his findings particularly on the specimens he collected in Chizé in 1987, noting significant differences with the descriptions of those from Jura… During our last private conversations on this subject, at the SMF session held in Morbihan in 2002, G. Redeuilh suggested that the type species *torosus* was probably the form of Jura, and that we had a different form in our own calcareous deciduous woodlands on mount Argenson, which might be considered an undescribed variety” (“Il se basait notamment sur les récoltes qu’il avait faites à Chizé en 1987, en relevant des différences sensibles avec les descriptions provenant du Jura… Lors de nos dernières conversations à ce sujet, en privé, à l’occasion de la session de la SMF tenue dans le Morbihan en 2002, Guy Redeuilh pensait que le type de l’espèce *torosus* était sans doute la forme du Jura, et que nous avions chez nous, dans les bois feuillus sur calcaire du massif d’Argenson, une forme différente qui, peut-être, pourrait être considérée comme une variété non décrite”; Fourré 2006: 282). The same Fourré (1992, 2003a, 2003b, 2004, 2006) reported on the occurrence in France of two different aspects of *B. torosus*, one of them being characterized by a deeply rooting stipe, a pileus soon brownish red and devoid of greyish or greenish tinges, duller yellow pores and growth under planted conifers (*A. alba*). He wrote: “This confirms that *Boletus torosus* occurs with two forms that share many similarities (notably their exceptional density; both are “heavy like a stone”), but also constant discrepancies and a very different ecology” (“Il se confirme ainsi que le *Boletus torosus* existe sous deux aspects qui ont beaucoup de points communs (notamment l’exceptionnelle densité, ils sont l’un et l’autre “lourds comme une pierre”), mais aussi des divergences constantes, et une écologie bien différente”; Fourré 2003a: 321), and also described the pale form growing under deciduous trees: “Also very abundant in some years, the [*B.*] *torosus* we find under deciduous trees (mainly oaks and hornbeams) is a massive, often versiform, very dense bolete (much heavier, at the same volume, than other boletes). Its pileus is nearly always of a somewhat pale, dirty gray, the stipe base is beetroot red, pores are bright yellow, intensely and immediately turn blue like the context at the slightest touch. When young, the cuticle is pale yellow, but this shade is so fleeting that it is very rarely observed; it readily turns dirty gray, and eventually may become irregularly reddish with age” (“Très abondant également certaines années, le torosus que nous rencontrons sous feuillus (chênes et charmes principalement) est un bolet massif, souvent difforme, très dense (beaucoup plus lourd, à volume égal, que les autres bolets), son chapeau est presque toujours d’un gris sale assez clair, la base du pied est rouge betterave, les pores jaune vif, bleuissant intensément et immédiatement, comme la chair, au moindre contact. A l’état jeune la cuticule est jaune clair mais cette nuance est si éphémère qu’on ne l’observe que très rarement, elle passe tout de suite au gris sale, et tout à fait à la fin, dans la vétusté, elle peut devenir rougeâtre, irrégulièrement”; Fourré 2004: 401). Fourré argued that: “Our dark-capped collections of “*torosus*” found under conifers would be […] the closest in appearance to the species known and described from the Dauphiné-Savoie and Jura. Therefore, the other form with a pale pileus growing under deciduous trees, by far the most abundant in our central-western region, should perhaps be named as a variety distinct from the type” (“Nos récoltes du “*torosus*” à habit sombre, sous résineux, seraient […] les plus proches d’aspect de l’espèce connue et décrite en Dauphiné-Savoie et Jura. C’est donc l’autre forme, de loin la plus abondante dans notre Centre-Ouest, celle à chapeau clair poussant sous feuillus, qui serait éventuellement à nommer comme variété différente du type”; Fourré 2004: 402). Fourré discussed the provisional name *B. torosus* var. *daniaudii* in connection with these two forms (Fourré 2003a, 2003b, 2004), but the published accounts do not consistently associate it with the same form. In the 2003b publication, he proposed this name for the dark-capped form growing under conifers (Fourré 2003b: 425). However, the same Fourré was farsighted enough when he claimed: “…the ‘variety *daniaudii*’ is likely to remain confidential for a long time and limited to the archives of the author of these lines!” (“la «variété *daniaudii*» risque de rester longtemps confidentielle et limitée aux propres fiches du signataire de ces lignes!”; Fourré 2003b: 425). An additional indication that such a split of *B. torosus* in two different species was perceived as taxonomically meaningful is the informal circulation of the label “*Boletus viscidoviridis*” in the north-eastern Italian Alps to separate the dark-coloured *B. torosus* from its more thermophilic, pale-coloured variant (F. Bellù, pers. comm.). Up to the present study, however, this cryptic species has remained elusive.

### “*torosus*” vs “occultus”: which is the true *Imperator torosus*?

Under the ICNafp (Art. 9.9), an epitype is an interpretative type that provides clarifying information supporting the established type of a name (Turland et al. 2025, Art. 9.9); the consequences of taxonomic disagreement between an epitype and the type it supports are addressed by Art. 9.20. The epitype designated by Simonini et al. (2025) should therefore be assessed in relation to the neotype designated by Bertolini and Simonini (2013), as they did not identify any original material, neither specimens nor illustrations. We find that the 2025 epitypification goes beyond providing interpretative detail: it departs from, and in effect reverses the delimitation that underpinned the neotypification. Rather than reinforcing the neotype-anchored concept, it broadens and shifts the name’s application in a way that is inconsistent with that framework.

In their precursor treatment, Simonini and Bertolini (2012: 35) explicitly separated an “entità a pori rossi” (entity with red pores, treated as *B. rhodopurpureus*) from an “entità a pori gialli” (entity with yellow pores, treated as *B. torosus*). This dichotomy aligns with the protologue’s signal of yellow pores as the baseline condition and reddening as a later maturational development (Secretan 1833; Fries and Hök 1835). The subsequent neotypification anchored *B. torosus* to that yellow-pored, “basane livide” phenotype via the Pilát and Dermek plate (Pilát and Dermek 1974; Bertolini and Simonini 2013: 119). In doing so, Bertolini and Simonini (2013: 119) explicitly framed *I. torosus* as a taxon conforming to the established Swiss tradition, and they treated the Pilát and Dermek iconography as the only plate they considered compatible with Secretan’s chromonym (Bertolini and Simonini 2013: 119). This plate also sits within the Swiss iconographic lineage running from Walty through later reproductions (Walty 1927a, 1927b, 1927c; Walty, Schweizer Pilztafeln II (Walty 1944, 1947, 1957, 1965); Singer 1967; Pilát and Dermek 1974). This Walty-based concept has remained operative in modern iconographic literature for almost a century. Faithful depictions appear in European Boletes 1: Supplement 1 (Mikšík and Polčák 2024) and in the recent monograph European Boletes 1 (Mikšík et al. 2025), which otherwise adopt the traditional interpretation while noting that some photographs labeled *Imperator cf. torosus* actually depict a different species (*I. occultus* sp. nov.; as *Imperator* sp. in Mikšík and Polčák 2024: 61).

**Figure 7.**
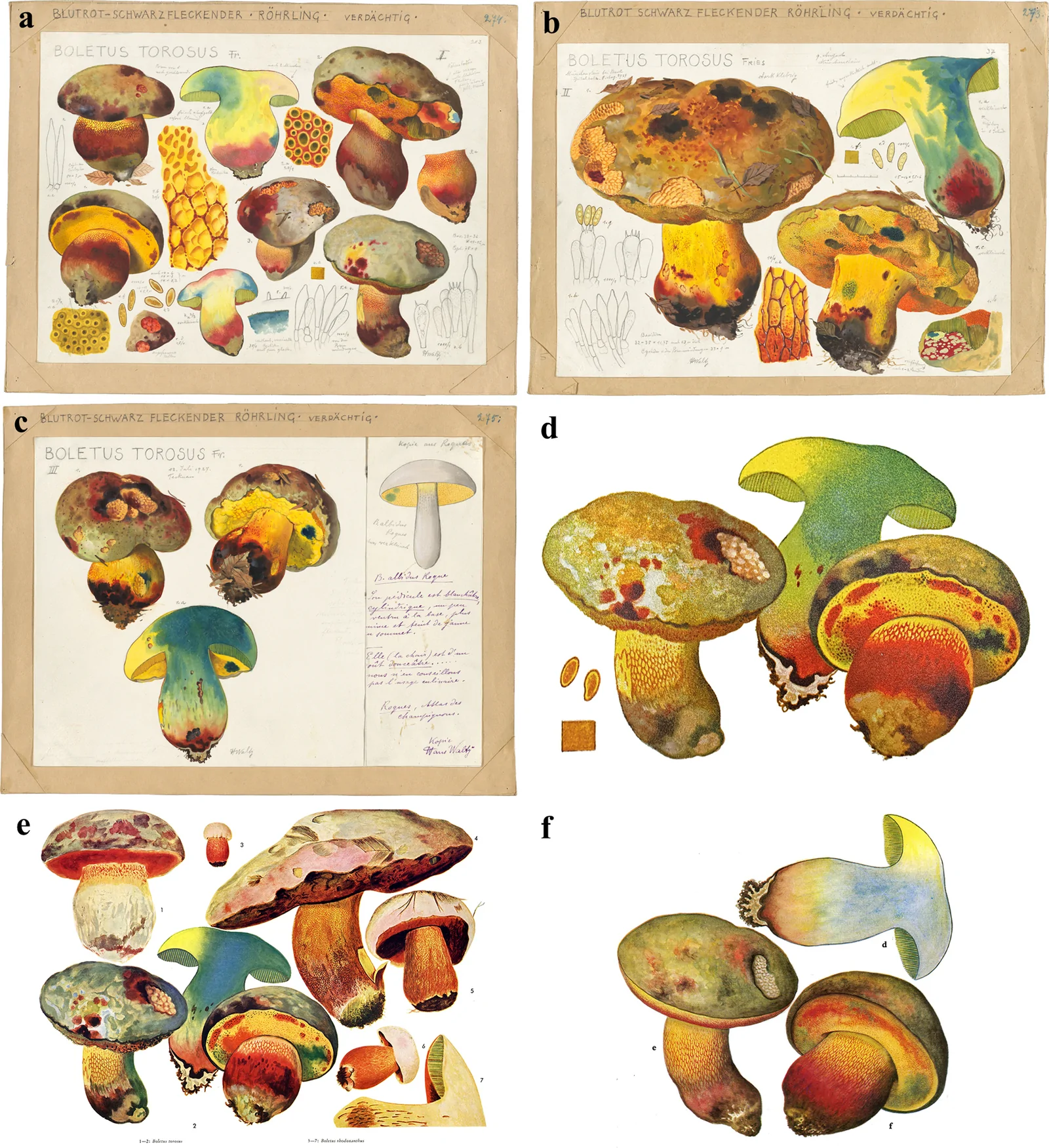
*Imperator torosus*, watercolours and illustrations. **a**. Walty H., 1915 (ca.) –1945 (ca.), Blutrot-schwarzfleckender Röhrling, verdächtig. Pilztafelwerk Band A, 274 (GS-WALTY-A-1-274; A-1-274). Swiss National Library, Prints & Drawings; **b**. Walty H., 8–9 Aug 1927, Blutrot schwarz fleckender Röhrling, verdächtig. Pilztafelwerk Band A, 273 (GS-WALTY-A-1-273; A-1-273). Swiss National Library, Prints & Drawings; **c**. Walty H., 12 Jul 1927, Blutrot-schwarz fleckender Röhrling, verdächtig. Pilztafelwerk Band A, 275 (GS-WALTY-A-1-275; A-1-275). Swiss National Library, Prints & Drawings; **d**. Walty H., 1965 (4^th^ edn), Schweizer Pilztafeln für den praktischen Pilzsammler, II. Band. Verband Schweizerischer Vereine für Pilzkunde, Zürich; **e**. Singer R., 1967, Die Röhrlinge. Teil II. Die Boletoideae und Strobilomycetaceae. Die Pilze Mitteleuropas Band 6. Verlag Julius Klinkhardt, Bad Heilbrunn; **f**. Pilát A. and Dermek A., 1974, Hríbovité huby. Československé hríbovité a sliziakovité huby (*Boletaceae* – *Gomphidiaceae*). Veda, Bratislava (neotype).

**Figure 8.**
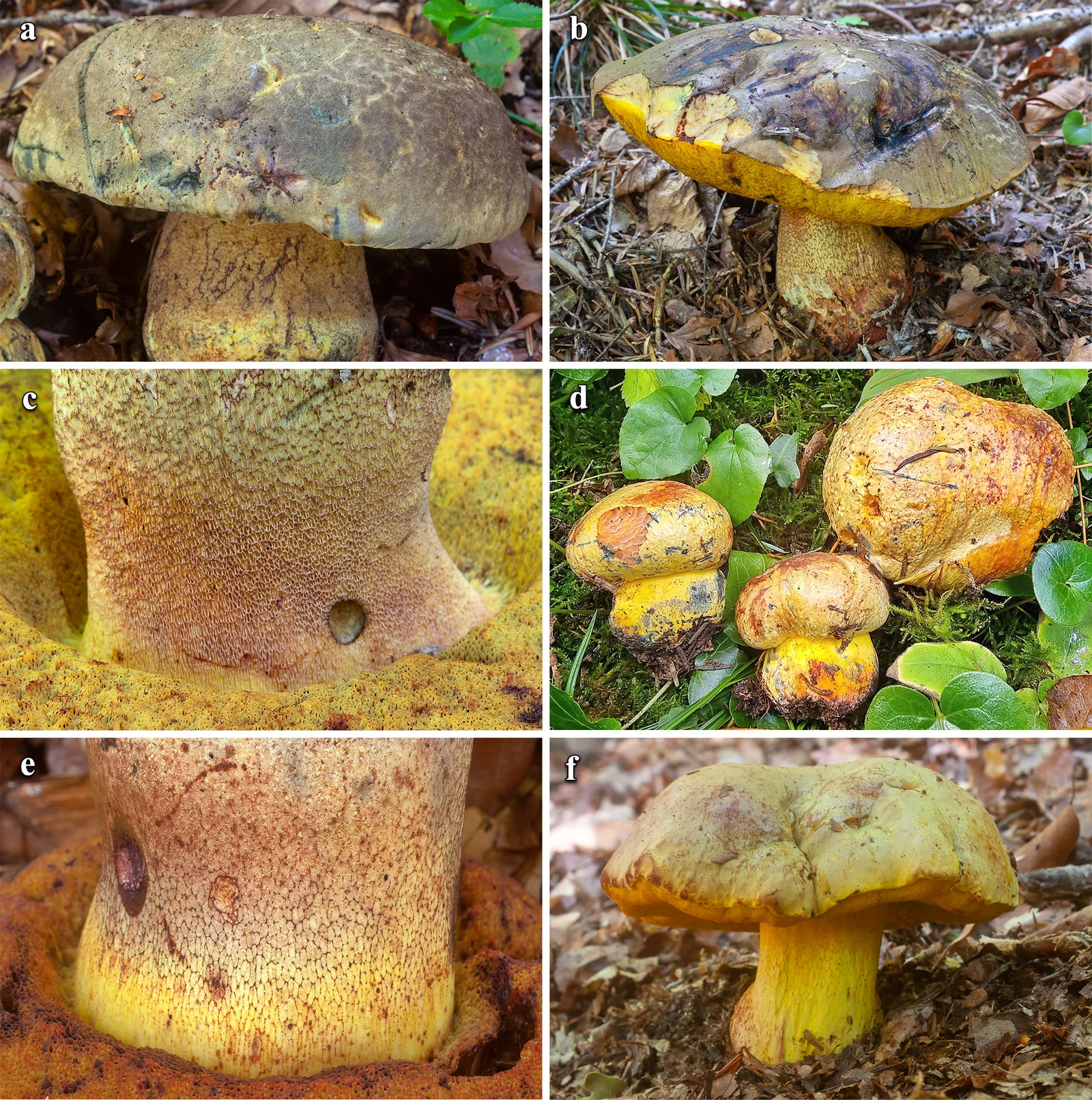
*Imperator torosus*. **a**. CNF 10/323, CROATIA, Primorje-Gorski Kotar County, Skrad (pileal tessellation); **b**. CNF 10/79, CROATIA, Primorje-Gorski Kotar County, Skrad (typical “basane livide” pileus colour; pileal tessellation locally developed); **c**. CNF 10/79, CROATIA, Primorje-Gorski Kotar County, Skrad (stipe reticulum with small, dense but raised, interwoven meshes); **d**. CNF 10/320, SLOVENIA, Črnomelj admin. unit, Stari trg ob Kolpi (“xanthoid form”); *Imperator occultus*. **e**. CNF 10/335, CROATIA, Zagreb County, Staničići Žumberački (stipe reticulum with faint and large meshes); **f**. CNF 10/319, CROATIA, City of Zagreb, between Planina Donja and Kašina (pileus with a warm yellow basal ground colour, covered by a fine whitish-creamy pruina; a stipe reticulum with faint and large meshes is also visible). Photos by N. Ferenčak (**a–c, e, f)** and E. Zupan (**d**).

**Figure 9.**
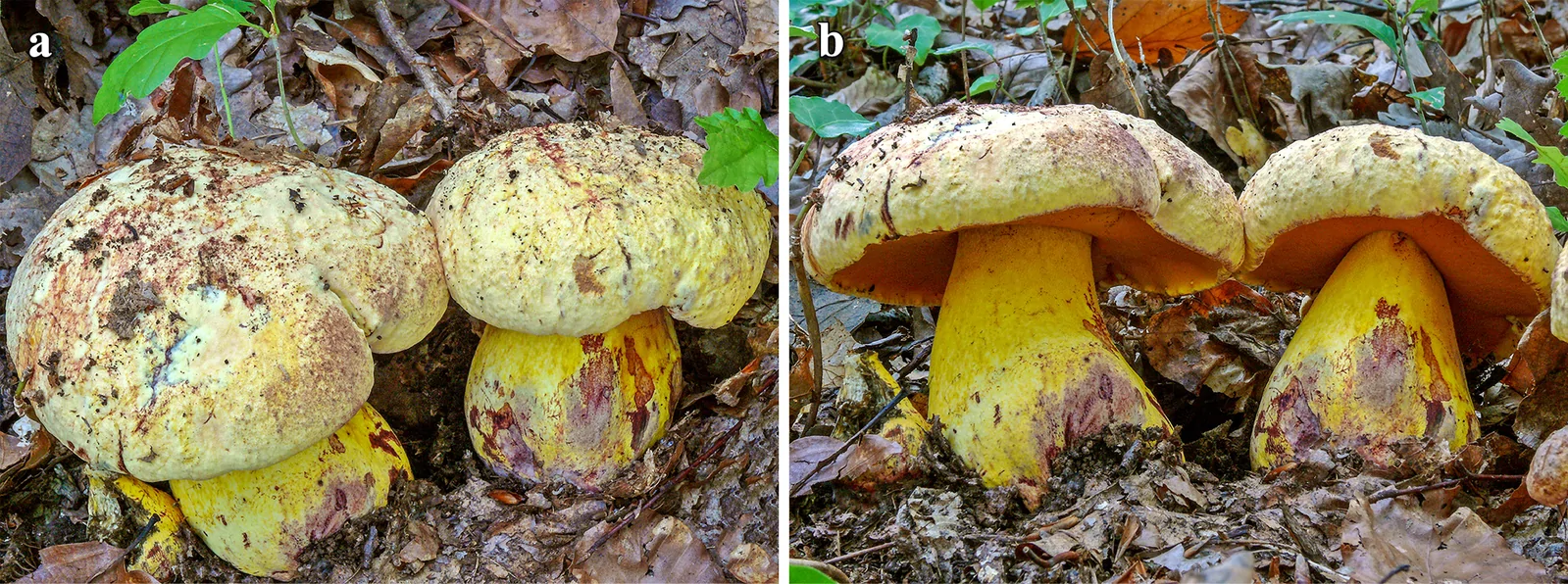
*Imperator rhodopurpureus* f. xanthopurpureus, basidiomes in habitat. **a, b**. CNF 10/53, CROATIA, Varaždin County, near the village of Gačice. Photos by N. Ferenčak (**a, b**).

Simonini et al. (2025) based their epitypification on the collection MCVE18453 from a thermophilous site in Castelvecchio (Adige Valley), documented in multiple photographs (figs 3–6). Their epitype treatment also explicitly incorporates additional illustrated conspecific collections (figs 7–11), including ‘more yellow’ pileus expressions, which they treat as belonging to the same species and use to support a predominantly xanthoid concept (Simonini et al. 2025: 34). Although they acknowledge that at least two distinct species level lineages exist within this complex (Simonini et al. 2025: 29), they nevertheless extend the application of *B. torosus* toward a predominantly xanthoid (“yellow-end”/yellow-based) concept. In fact, Simonini et al. (2025: 29, 34) explicitly state that the “more yellow” pileus expressions shown to belong to the same species may be treated as synonyms of *I. torosus*, and they likewise treat the xanthoid “*xanthocyaneus*” form as a synonym of *B. torosus*. This approach is the opposite of the 2013 stabilization logic: Bertolini and Simonini (2013: 119) associated xanthoid “*xanthocyaneus*” material with the *I. rhodopurpureus* complex rather than with *I. torosus*. Consequently, the 2025 epitypification alters rather than clarifies the name’s application. Instead of reinforcing the neotype-anchored concept, the epitype shifts it into a competing morpho-ecological frame, contrary to the intended subordinate role of an epitype once a name has been stabilized by an accepted type and an established interpretative tradition (Turland et al. 2025).

By assigning “more yellow” expressions to *B. torosus* and treating the xanthoid “xanthocyaneus” entity as a synonym without publishing independently verifiable molecular support for that synonymy (Simonini et al. 2025: 34), the epitype concept becomes broad enough to encompass yellow-dominant morphotypes arising in more than one evolutionary lineage within the complex. This non-specificity of a yellow-biased frame is also consistent with earlier field syntheses that explicitly distinguished a montane *Fagus*/conifer-associated cohort from lowland *Quercus*-associated material under “*Boletus torosus*”, underscoring that “more yellow” appearance is not, by itself, lineage-diagnostic in this complex (Estadès and Deïana 2004: 23, 26; Fourré 2003a: 321; Fourré 2006: 282). At minimum, this predominantly xanthoid macromorphological envelope overlaps three distinct taxa: (1) *I. torosus* s. Secretan/Fries, in which yellow-dominant basidiomes can occur as rare internal variants (this study; CNF 10/320), (2) *I. occultus* sp. nov., a naturally semi-xanthoid species (this study), and (3) xanthoid form of *I. rhodopurpureus* (= *I. rhodopurpureus* f. xanthopurpureus), historically linked with “*xanthocyaneus*”-like material (Bertolini and Simonini 2013: 119). Notably, within *I. torosus* sensu Secretan/Fries as here delimited, an almost uniformly yellow (xanthoid) pileus has so far been observed only once in our material, in a Slovenian collection from Stari Trg ob Kolpi for which sequence data were generated and the material preserved as a vouchered, fungarium-deposited specimen in the Croatian National Fungarium (CNF 10/320). This is interpreted as an exceptional chromatic anomaly rather than a colour form characteristic of the species. This demonstrates that a predominantly xanthoid phenotype alone cannot establish the precise lineage identity of the epitype or the conspecificity of the additional illustrated collections. Moreover, xanthoid expressions historically associated with “*xanthocyaneus*”-like material have also been treated within the *I. rhodopurpureus* complex (Bertolini and Simonini 2013: 119). Without sequence data generated from the epitype specimen itself and publicly verifiable accessions, a predominantly xanthoid macromorphological frame is therefore intrinsically non-diagnostic at the typification level, and the published evidence does not permit independent verification of whether the designated epitype (MCVE18453) and the associated illustrated collections treated as conspecific all belong to a single lineage, or which lineage or lineages they represent (Simonini et al. 2025: 29; Turland et al. 2025, Art. 9.9).

This displacement is also evident in the way Simonini et al. (2025) motivate the epitypification. In the abstract, they explicitly frame the move as a corrective response to problems in the neotype-related iconography, writing “Given the existing ambiguities in the plate representing the neotype of *Boletus torosus* Fr.” (“Stanti le ambiguità esistenti sulla tavola rappresentante il neotypus di *Boletus torosus* Fr.”; Simonini et al. 2025: 29). In the introduction, they sharpen this claim by stating that the Pilát and Dermek (1974) neotype-related plate (ultimately derived/reproduced from Walty via the Schweizer Pilztafeln für den praktischen Pilzsammler series, vol. II) is “now to be considered not sufficiently representative” (“ora da ritenere non sufficientemente rappresentativa”), and they explicitly ground this in Art. 9.9 by quoting that a type “cannot be critically identified for purposes of the precise application of the name to a taxon” (Simonini et al. 2025: 30; Turland et al. 2025, Art. 9.9). In practical terms, this frames the neotype-related plate as insufficiently exhaustive for the precise application of the name. That rationale directly conflicts with the immediately preceding neotype-era position. In their precursor synthesis, Simonini and Bertolini (2012: 32) described Pilát and Dermek’s plate as “a good copy of Walty’s plate” (“buona copia della tavola di Walty”) and classified it with the code [***P], within a key defined as “Iconography indicated as the best representation of the species concept of the taxon” (“Iconografia segnalata come migliore rappresentazione del concetto specifico del taxon”; Simonini and Bertolini 2012: 32). This represents a substantial change in interpretation between the treatment by Simonini and Bertolini (2012) and the epitypification by Simonini et al. (2025): Simonini and Bertolini (2012: 32) described the Pilát and Dermek plate (Tav. 65 d-f) as “a good copy of Walty’s plate” (“buona copia della tavola di Walty”) and coded it [***P], whereas Simonini et al. (2025: 30) stated that the same neotype plate was not sufficiently representative for the precise application of the name. In the 2013 neotypification paper, Bertolini and Simonini stated that the colouration of the pileus cuticle of the bolete they proposed as *B. torosus* was “the only one that, in our view, can correspond to ‘basane livide’” (“la sola che, a nostro avviso, può corrispondere al «basane livide»”; Bertolini and Simonini 2013: 119) and designated the Pilát and Dermek plate as the neotype, thereby formally anchoring the application of the name to that iconographic type (Bertolini and Simonini 2013: 119–120). In other words, the 2025 epitype is not deployed as a subordinate interpretative aid to an already stabilised type concept. Rather than taking the neotype-anchored concept as given and adding narrowly targeted information to improve its critical identifiability, the epitypification is justified by the claim that the neotype-related plate is itself no longer sufficiently representative for precise application. On that premise, the epitype serves not as an auxiliary reference attached to the neotype, but as a corrective tool that effectively reopens the settled interpretative frame and resets the diagnostic baseline under the same name (Simonini and Bertolini 2012: 32; Bertolini and Simonini 2013: 119; Simonini et al. 2025: 29–30; Turland et al. 2025, Art. 9.9).

A further structural issue is the absence of publicly verifiable molecular anchoring for the epitype itself. Simonini et al. (2025) repeatedly invoke “ITS-based molecular investigations” (“investigazioni molecolari basate su ITS”), explicitly attributing this support to work carried out independently by M. Bianchi in 2019 and G. Ambrosio in 2022 (unpublished data) (Simonini et al. 2025: 29). However, the epitype treatment provides no sequence data for the epitype individual, nor any accessioned sequence references that would allow independent verification of which collections were sequenced and how they relate to the designated epitype (MCVE18453; figs 3–6), or to the additional illustrated collections invoked to delimit the species’ phenotypic range (figs 7–11) (Simonini et al. 2025). Accordingly, the unpublished molecular evidence cited by Simonini et al. (2025) in the context of their conclusion that the neotype plate is insufficiently representative is not independently auditable from that paper, because no accessioned sequences or specimen-to-sequence linkages are provided for the epitype or the associated illustrated material. In a complex explicitly acknowledged to contain multiple entities, an epitype that is not itself sequence-anchored is permissive rather than conservative in its typifying role (Simonini et al. 2025; Turland et al. 2025).

Epitypification in a multi-entity complex ideally strengthens the name’s application by adding independently verifiable diagnostic information beyond macromorphology, thereby tightening the link between the name and a reproducible set of characters. In the 2025 treatment, however, the authors explicitly state that the study is “deliberately limited to morphological characters” (“volutamente limitato ai caratteri morfologici”; Simonini et al. 2025: 30) and that future work will “necessarily” (“necessariamente”) involve “microscopic and molecular characters” (“caratteri microscopici e molecolari”; Simonini et al. 2025: 31), i.e., these axes are deferred rather than documented for the epitype individual. As a result, the epitype concept remains predominantly photographic and macromorphological. Even where the paper asserts conspecificity for additional illustrated collections “by microscopic and also by molecular means” (“per via microscopica e anche per via molecolare”; Simonini et al. 2025: 36), it does not provide an epitype-individual microscopic character set (e.g. basidiospore metrics, cystidia, pileipellis, etc.), nor does it present any traceable molecular outputs (e.g. accessioned sequences, sequence listings, phylogenetic trees) that would allow independent assessment of which illustrated specimens were examined and how they relate to the epitype. In a complex acknowledged to contain multiple entities, this combination reduces rather than increases the epitype’s discriminative and stabilising value at exactly the level where Art. 9.9 expects improved critical identifiability and precision of application (Simonini et al. 2025; Turland et al. 2025, Art. 9.9).

From a morphological perspective, the shift introduced by the epitype is most evident in terms of pileus colouration. This can be examined at three interlocking levels: (a) the protologue terminology, (b) the iconographic tradition that sustained prevailing usage in Swiss and central-European literature, and (c) the concrete phenotype documented in the epitype figure itself. Secretan’s decisive colour term for this species was “basane livide” (Secretan 1833). Fries himself explicitly noted he had “not seen” *B. torosus* (Fries 1838: “non vidi”), so his description (“fuscescente”, becoming brownish) was not based on direct observation and is inherently vague, lacking the chromatic precision of Secretan’s “basane livide” usage. In Secretan’s usage, “basane” functions as a baseline colour term and “livide” as a secondary modifier. Taken together, basane livide indicates a vegetable-tanned leather brown (basane) with a livid, lead-coloured bluish-grey cast tending towards black (Littré: “plombé tirant sur le noir”) (Dictionnaire de l’Académie françoise (1798, 5^th^ edn); Dictionnaire de l’Académie française (1992–2024) (9^th^ edn); TLFi/CNRTL; OQLF; Littré 1863–1873; Société Mycologique des Montagnes Neuchâteloises [SMMN] 2003: Glossaire (Septembre 2003)). In practical terms, it refers to a muted, grey-tinged leather-brown hue, not a warm buff-ochre or uniformly yellow-dominant palette. In the same work (Secretan 1833), Secretan’s usage confirms basane as a baseline chromatic term: he referred to *B. alutaceus* (i.e. *Leccinellum pseudoscabrum*) as “basane bolete with a rough stipe” (“Bolet basane à pied rude”) and described it as “yellowish grey” (“gris jaunâtre”). He also described *B. radicans* as “light basane, slightly greenish” (“basane clair, un peu verdâtre”), showing that he deliberately uses basane as a baseline chromatic term and then modulates it with a second qualifier when needed. Accordingly, basane livide cannot be read as a “light” and predominantly yellowish, warm-toned basane concept of the kind depicted in the 2025 epitype figure, which shows no stable grey or leaden cast comparable to the neotype tradition. When Secretan intended to indicate a lighter basane shade, he explicitly marked it as such (e.g. “basane clair”), and when he described a basane-labelled taxon as yellowish grey he stated this directly (e.g. “Bolet basane à pied rude”, “gris jaunâtre”). This semantic distinction is not mere “antiquarian” pedantry; it was the key interpretative link used by Bertolini and Simonini when they chose Pilát and Dermek’s 1974 colour plate as the only iconography they regarded as compatible with Secretan’s basane livide chromonym (Bertolini and Simonini 2013: 119). In short, “basane livide” defines a grey-cast leather-brown pileus concept, and it underpins the neotype interpretation.

It should be emphasised that Walty’s original 1927 watercolours were executed with a high degree of precision and attention to detail, faithfully reflecting the modern concept of *I. torosus* and exhibiting clear macromorphological congruence with our own documented material. Crucially, the neotype plate adopted in 2013 (Pilát and Dermek 1974) stands at the downstream endpoint of an iconographic continuity. Walty’s original 1927 watercolours (Walty 1927a, 1927b, 1927c; A-273, A-274 and A-275) comprise three original paintings of *B. torosus*, preserved in the Hans Walty archive (Swiss National Library, Prints & Drawings Dept.). Published reproductions derived from these originals entered the Swiss literature through the “Schweizer Pilztafeln für den praktischen Pilzsammler” series issued by the Verband Schweizerischer Vereine für Pilzkunde, first published as vol. II in 1944 and later reissued. The *B. torosus* plate occurs in vol. II of that series, and the printed reproduction is recognisably Walty-derived but necessarily simplifies some fine details relative to the preserved originals, which remain the more precise reference. These illustrations subsequently circulated through later editions of the “Schweizer Pilztafeln” series and through reproduction by later authors (e.g., Singer 1967) and were subsequently incorporated by Pilát and Dermek (1974). Notably, well before the formal 2013 neotypification, Estadès and Deïana (2004) noted that Walty’s plate (Walty 1947, pl. 66) could be proposed as a neotype in the absence of any other eligible representation or material. Although intended as a proposal rather than a formal designation, this is important because the same passage also notes that Walty’s herbarium could not be traced in the major Swiss institutions and briefly traces the subsequent iconographic reception of Walty’s plate by Singer (1967), Pilát and Dermek (1974), and Dermek and Pilát (1988), i.e. the same downstream iconographic sources that underpinned the neotype-related tradition ultimately formalised by the 2013 neotype designation. This chain of illustrations documents a community concept of *B. torosus* that predates modern typification.

Against that background, the published epitype figure (epitype specimen MCVE18453) presents a characteristically warm pileus palette. The youngest basidiomes are pale warm buff (straw-cream to very light ochraceous), while the fully expanded specimen becomes mahogany-russet (bay-brown), with no evident lead-grey to livid-olivaceous ground colour comparable to the neotype tradition (Simonini et al. 2025). This xanthoid departure is not confined to the epitype specimen. The published photographs of the additional illustrated collections likewise consistently show pale warm buff to straw-cream or light-ochraceous pilei, and none exhibit a stable lead-grey or grey-brown, livid-cast (“basane livide”) pileus baseline comparable to the neotype iconography (Simonini et al. 2025).

The mismatch is sharpened by an internal inconsistency in the 2025 treatment itself. The published description reports grey-olivaceous to “greenish” pileus tones, stating that the pileus may become “even pale green” (“fino anche a verde pallido”) and that mature basidiomes may assume “grey-green-ochraceous to brown-olivaceous tones” (“tinte grigio-verde-ocracee, bruno-olivastre”). Yet the same text immediately qualifies this by noting that “It has repeatedly been observed that the greenish colouration of the pileus occurs or is particularly accentuated after collection, or already on the ground in old and/or desiccated specimens” (“Si è ripetutamente osservato che la colorazione verdastra del cappello si manifesta o si enfatizza particolarmente dopo la raccolta, oppure già sul terreno in esemplari vetusti e/o disidratati”). The authors contrast this statement with “more yellow chromatic expressions” (“espressioni cromatiche più gialle”), which show no tendency to assume green tones (Simonini et al. 2025: 34). The published epitype images (figs 3–6) and the associated pictures (figs 7–11), however, predominantly document intact basidiomes with yellowish to warm-coloured pilei lacking any stable grey-olivaceous or green ground tint. Where “greenish” hues are perceptible in the photographs, they are confined to localised patches associated with handling or bruising, where blue-black oxidation over a yellow base is visually evident. Because of the published images, such localised post-collection discolouration cannot be treated as a stable, diagnostic pileus ground colour (Simonini et al. 2025: 34). Consistent with this, the same pattern is observed in the naturally xanthoid species level lineage in the complex that we treat here as *I. occultus*. In *I. occultus*, the pileus has a warm yellow ground colour from an early stage, initially masked by a fine whitish to pale greyish pruina. Subsequent warm ochraceous to copper-brown discolouration is most plausibly associated with bruising or desiccation, although ageing and deterioration may also contribute. These changes and the pale surface pruina should be distinguished from the characteristic leaden-grey to grey-cast leather-brown pileus baseline of *I. torosus* s. str., which has not been observed in the *I. occultus* collections examined here.

Notably, Secretan (1833) also reported a characteristic transition of the pileus surface: it “turns yellowish and becomes tessellated into brownish compartments” (“il tourne ensuite au jaunâtre et se tessèle par compartiments brunâtres”), with the yellow ground visible between the cracks. In one of our sequenced Croatian *I. torosus* collections from Skrad (Gorski kotar, Croatia; CNF 10/323), the pileus surface shows the same combination, expressed as a brownish, cracked (tessellated) pruina through which the yellow underlying pileus tissue became clearly visible between the fissures. This matches the protologue, has not been observed in any examined *I. occultus* collections, and is not documented in the published epitype figure or in the photographs of the additional illustrated collections in Simonini et al. (2025).

Another protologue-linked surface character supports the same conclusion. In Secretan’s protologue, the pileus surface is explicitly described as roughened (“La surface est raboteuse”) (Secretan 1833). By contrast, Simonini et al. (2025: 34) describe the epitype’s pileus cuticle as “cuticle initially finely velvety, then glabrous…minutely corrugated” (“Cuticola inizialmente finemente vellutata, poi glabra…minutamente corrugata”). Consistently, our neotype-aligned material likewise shows a conspicuously roughened pileus surface in line with Secretan’s formulation.

The same directional shift is evident in stipe ornamentation. Secretan noted a densely arranged, brown reticulum especially in the upper part (“Un réseau brun à mailles serrées…surtout dans le haut”) (Secretan 1833), whereas the 2025 epitype concept is described as having “a very fine reticulation, in relief” (“un reticolo molto fine, in rilievo”) (Simonini et al. 2025: 36). In line with the neotype tradition as formalised in 2013, our neotype-aligned material likewise shows a distinct, prominently raised apical reticulum with dense meshes, most evident in the upper third of the stipe, where the network may appear irregularly interwoven and locally anastomosing (Pilát and Dermek 1974; Bertolini and Simonini 2013; this study). By contrast, in *I. occultus* the stipe reticulum is less pronounced than in *I. torosus*. The reticulum tends to broaden and elongate toward the stipe base, often becoming evanescent so that it can be almost indistinct in older specimens. At the apex, the reticulum remains more regularly arranged and far less prominently raised, lacking the fine, intricately interwoven, locally overlapping meshes characteristic of the true *I. torosus*.

The pore-colour trajectory provides a further point of comparison. The protologue and early Friesian diagnosis describe an ontogenetic sequence in which pores are yellow in youth and may later become red. Fries summarised this as “tubes yellow, then reddening” (“tubulis … luteis, dein rufescentibus”; Fries and Hök 1835). Secretan likewise framed the shift as maturational, beginning with “yellowish in infancy…beautiful egg-yolk yellow” (“jaunâtre dans l’enfance…beau jaune d’œuf”) and ending with pores that become “red … shading to orange” (“rouge…se nuançant à l’orangé”; Secretan 1833). Bertolini and Simonini (2013) treated this late-reddening trajectory as a core interpretative anchor for nomenclatural stability, essentially defining *I. torosus* as a bolete with yellow pores that only tardively turn red, and contrasting it with *I. rhodopurpureus* and *I. luteocupreus*, whose pores are essentially red and only transiently yellow at the earliest stage of development (Bertolini and Simonini 2013). The same concept had already been made explicit in 2012, separating a “red-pored” entity assigned to *I. rhodopurpureus* from a “yellow-pored” entity assigned to *I. torosus* within the complex (Simonini and Bertolini 2012: 35). The neotype iconography adopted in 2013 is wholly coherent with an interpretation in which yellow pores are the baseline condition and any reddening is optional and develops only later (Pilát and Dermek 1974; Bertolini and Simonini 2013). A recent first record from Türkiye provides modern photographic support for possible pore reddening and for the neotype-linked “basane livide” pileus framing. In fig. 1, Gürzoğlu et al. (2024: 23) illustrate basidiomes spanning through different developmental stages and including pilei with a distinctly livid to olivaceous-brown ground. Across the same figure set, some basidiomes show reddish pores (see fig. 1, p. 23, lower-right image). The marginal pore zone may retain a more yellow tone, yet shows incipient red suffusion, while the inner pore field shows a clear reddish cast. This supports the protologue-linked framing in which pores are typically yellow and may occasionally redden, particularly in aged basidiomes, rather than being constitutively red from early stages.

By contrast, in Simonini et al. (2025) the pore-ontogeny character is rather permissive. They emphasised that a natural but rather tardy reddening of the pores is “absolutely singular” (“assolutamente singolare”) and “a peculiarity that cannot be underestimated” (“una singolarità che non può essere sottovalutata”) for *I. torosus*, yet they simultaneously acknowledged that the character is “inconstant” (“incostante”). In the epitype description, the dissepiments are said to remain golden-yellow for a long time and only facultatively become dark red-orange from a medium stage of maturity onward. Crucially, however, they stated that this character “has not been observed directly” (“non è stato osservato direttamente”) in the epitype collection (MCVE18453), with only an incipient reddening appearing after some hours in “traumatized zones” (“zone traumatizzate”). To support this character, Simonini et al. (2025) refer to other collections illustrated in the paper, in which pore reddening is more evident and which are treated as conspecific “on a molecular basis” (“su base molecolare”). However, although the paper reports that molecular work was undertaken, it provides no sequence data generated from the epitype specimen itself, publicly accessible accessioned sequence data, specimen-to-sequence linkages, sequence listings, phylogenetic trees, or other traceable molecular outputs for either the epitype or the additional collections presented as conspecific. They further concluded that the appearance of red in the pores may be facultative, occurring from mid-maturity or only at extreme maturity. The published photographs of the additional illustrated material show pronounced pore reddening already in basidiomes that are evidently not at extreme maturity (figs 9, 10), while another figure documents only slight, partial reddening at extreme maturity (fig. 11). The absence of natural pore reddening in the epitype is not, by itself, inconsistent with *I. torosus*, in which pores typically remain yellow. The trauma-associated reddening reported after collection should nevertheless be distinguished from natural colour changes with age. In a species complex explicitly deemed to contain multiple entities, an epitype treatment relying on additional collections without a publicly traceable molecular link to the epitype specimen itself cannot be regarded as interpretatively conservative. It is structurally permissive, increasing rather than reducing the risk that distinct entities are being conflated under a single name, contrary to the intended clarificatory role of an epitype under Art. 9.9.

From an ecological perspective, the published claim of equivalence between Sauvabelin and Castelvecchio is not supported by the documented habitat contexts. Secretan’s original habitat remark, “under dense beeches, in a moist place” (“sous les hêtres touffus, en lieu humide”), directly identifies a moist, dense-beech microsite at Sauvabelin (Secretan 1833: 25). The later locality descriptions and official sampled-point data provide no documentation of a natural thermophilous forest assemblage at the published focal point; they do not replace Secretan’s historical observation (Galland 1987; Clot and Delarze 2009; ILEX 2025; État de Vaud n.d. [Phytosociologie forestière vaudoise]). At Castelvecchio, *Fagus* is likewise prevalent, as Simonini et al. (2025: 32) explicitly reported, together with *Quercus*, mainly *Q. pubescens*. The official Bu2 assignment and surrounding forest-type mosaic place that locality in a warm/submontane South-Alpine beech setting with a substantial thermophilous component (Autonomous Province of Bolzano–South Tyrol 2010: 56 [Waldtypisierung Südtirol, Band 2], 2011 [ForestTypes]; Autonomous Province of Bolzano–South Tyrol n.d. [Bu2 type sheet]). This contrast concerns the documented ecological setting: the presence of beech at both localities does not establish their equivalence.

The associated bolete records independently support this comparison. Simonini et al. (2025: 32) described the Castelvecchio collecting area as rich in thermophilous boletes and listed six accompanying species, including the thermophilous *R. lupinus*. Of these six listed species, only *R. satanas* is documented in the official SwissFungi records explicitly naming Sauvabelin. The other five are not documented in the locality-specific records examined (Gross et al. 2024). The Sauvabelin *R. satanas* record is modern (2013) and therefore does not infer the fungal assemblage of 1833. Its presence is compatible with the warmer contemporary climatic context but cannot be projected back onto the cooler historical conditions considered here (MeteoSwiss [1991–2020 gridded normals]; Imfeld et al. 2023). Thus, the modern SwissFungi record set does not reproduce the thermophilous assemblage reported for Castelvecchio, and the single shared species does not substantiate the proposed ecological “twinning”.

Climatologically, the “edaphic/climatic twin” narrative proposed by Simonini et al. (2025) is not supported by the climatic contexts relevant to the historical account and the epitype. Simonini et al. (2025: 33) inferred comparable precipitation from Climate-Data.org values for Lausanne and Bolzano. Our comparison instead uses published reconstructions at the Sauvabelin focal point and official Monticolo observations close to the elevation of the epitype locality, with equally long reference windows (Table 3).

Across these windows, Monticolo’s August mean temperature and mean daily maximum exceed the reconstructed Sauvabelin values by approximately 4.5 °C and 7.1 °C, respectively. Annual mean temperature and mean daily maximum were also higher at Monticolo, by approximately 3.1 °C and 5.5 °C, respectively. The thermal contrast therefore extends beyond a single unusually cool August and is also evident in the annual means. Annual precipitation is also lower at Monticolo, although the August precipitation totals are comparatively similar. Together with Secretan’s description of a moist microsite under dense beech trees, the marked thermal contrast contradicts the historical Sauvabelin setting as a climatic equivalent of the epitype setting.

The climatic evidence thus reinforces the habitat and assemblage differences discussed above: the environmental context of the modern epitype cannot be equated to that of the historical Swiss account. The 37-year comparison concerns these two contexts, encompassing both their geographic and temporal differences.

The soil evidence does not support treating the two locality frames as pedological equivalents. Simonini et al. (2025: 33) stated that “the nature of the soil at Sauvabelin and Castelvecchio is distinctly calcareous” (“la natura del suolo di Sauvabelin e Castelvecchio risulta spiccatamente calcarea”). Available independent sources do not confirm that shared local characterization. For the Lausanne frame, the WSL LWF plot at Bois-du-Benenté provides a measured regional reference approximately 5.9 km distant from the Sauvabelin focal point. Its investigated profile is a deep, weakly pseudogleyed Dystric Cambisol developed in Würm moraine within the regional Molasse setting, with pronounced stoniness mainly at greater depth (Walthert et al. 2003). For Castelvecchio, the official regional manual states that Bolzano quartz porphyry reaches the Altenburg terraces (Autonomous Province of Bolzano–South Tyrol 2010: 169–171), while the official ForestTypes polygon at the published epitype coordinate is Bu2, whose forest-type description sheet describes medium-to-deep, base-poor, partly podzolised brown soils on silicate substrates with moderate to very high skeletal content. Regional proximity to carbonate formations therefore does not establish calcareous soil at the epitype point. SoilGrids median predictions partly overlap in pH (5.6–6.2 at Sauvabelin; 5.8–6.7 at Castelvecchio), whereas coarse-fragment content shows a clearer structural contrast: 5–9 vol.% versus 19–31 vol.%, respectively (Suppl. material 2: table SS2; Poggio et al. 2021). The latter agrees directionally with the skeletal Bu2 soil description. LUCAS/JRC topsoil physical-property products provide broader European context for coarse fragments and Available Water Capacity, reported for the fine-earth fraction, but no site-specific AWC value is inferred (Ballabio et al. 2016; European Commission, Joint Research Centre 2026). Taken together, the regional evidence and standardized point estimates do not support pedological “twinning” of Sauvabelin and Castelvecchio; they document partially overlapping pH predictions within contrasting regional substrate and soil-structure contexts.

There is also an independent, geographically external warning sign against the interpretative value of the 2025 epitype, provided by kilometre-scale near-sympatry in southeastern Slovenia. Two distinct entities currently treated within the “torosus complex”, *I. occultus* and *I. torosus* in the neotype/protologue sense, have been documented in this study fruiting within the same broader landscape of the Dinaric carbonate-karst belt (Vrščaj et al. 2017; BGR/IAH/KIT/UNESCO 2017 [WOKAM karst aquifer map]; Hartmann and Moosdorf 2012 [GLiM global lithological map]; Geological Survey of Slovenia GeoZS portal, GeoZS GIS/Map portal). Specifically, *I. occultus* (LJF 8893) was collected near Podlesje (Kočevje admin. unit; 45°30'10.8"N, 14°59'31.8"E), whereas *I. torosus* (CNF 10/320) was collected near Stari Trg ob Kolpi (45°30'55.6"N, 15°04'41.4"E). The two points are separated by only ~6.8 km in great-circle distance. Importantly, these are not merely “morphotypes”: both vouchers represent molecularly confirmed, species level lineages based on newly generated sequence data. According to field observations, *I. occultus* at Podlesje (LJF 8893) occurred under *Fagus sylvatica*, *Quercus cerris*, *Q. petraea*, *Carpinus betulus* and *Fraxinus* sp. in a microsite with a thermophilous vegetation component, whereas *I. torosus* s. str. at Stari Trg ob Kolpi (CNF 10/320) occurred in a mesic mixed forest under *Fagus sylvatica* and *Abies alba*. These geographically close records therefore document contrasting microsites, not syntopy or habitat equivalence. They do not overturn the ecological contrast between Secretan’s moist beech microsite and the warm/submontane setting reported for the epitype locality. More broadly, our sequence-based dataset shows that all three relevant *Imperator* taxa, *I. torosus* sensu Secretan/Fries, *I. occultus* and the xanthoid *I. rhodopurpureus*, occur across temperate Europe, in multiple countries and across a broad range of latitudes, elevations and forest types (this study). Against that pan-European backdrop, the 2025 epitype concept is geographically narrow: the epitype and all associated collections used to support it are drawn exclusively from northern Italy, and the coordinates provided show them clustered within approximately 240 km of the epitype locality and within approximately 330 km of one another (Simonini et al. 2025: 39). It is therefore methodologically problematic to generalise from this geographically narrow Italian subset and to use it as the primary basis for redefining the name *Boletus torosus*, which was originally described from Swiss material at Sauvabelin (Secretan 1833). Consequently, anchoring the name “torosus” to a “thermophilous” locality via epitypification cannot be deemed as an interpretative clarification by appeal to ecology. Instead, it increases the risk of lineage substitution, particularly in a complex where distinct lineages can occur in close geographical proximity while occupying dissimilar microsites. In precisely such circumstances, an epitype must be maximally conservative with respect to the historically stabilised neotype and protologue concept and, ideally, directly sequence-anchored on the epitype individual in order to align with the stabilising intent of Art. 9.9 (Turland et al. 2025).

All the above discrepancies, morphological, ecological, climatic, and edaphic, would already be serious even if the epitype framework were independently verifiable and unambiguously diagnostic. However, Simonini et al. (2025) acknowledged that the “torosus complex” includes “at least two molecularly distinct species” (“almeno due specie molecolarmente differenti”) (Simonini et al. 2025: 29), while also stating that their contribution is “deliberately limited to morphological characters” (“volutamente limitato ai caratteri morfologici”; Simonini et al. 2025: 30). Although they referred to “investigazioni molecolari basate su ITS” (“ITS-based molecular investigations”) attributed to third parties (Simonini et al. 2025: 29), the epitype treatment itself provides no epitype-individual sequence data or accessioned molecular outputs that would allow independent verification of lineage identity. This combination, explicitly recognised plurality within the complex and a typification-level framework constrained to macromorphology without traceable molecular anchoring, is exactly the scenario in which an epitype can structurally represent a lineage different from the historically stabilised name concept.

Under the ICNafp, an epitype is meant to enable “critical identification” and thereby secure precise application of a name when the existing type “cannot be critically identified for purposes of the precise application of the name to a taxon” (Turland et al. 2025, Art. 9.9, as quoted and invoked by Simonini et al. 2025: 30). Yet the 2013 neotypification was explicitly designed to resolve the interpretative problem by selecting a single iconographic anchor judged compatible with Secretan’s “basane livide” concept, namely the colour plate in Pilát and Dermek (1974, Tav. 65 d-f), treated as an improved, more faithful rendering of the Walty tradition (Pilát and Dermek 1974; Walty 1944, 1947, 1957, 1965; Singer 1967; Bertolini and Simonini 2013: 119). Unfortunately, the 2025 epitype introduces a competing interpretative anchor that departs along the pileus-colour axis (a predominantly xanthoid to warm-toned pileus palette); it is ecologically thermophilous, and climatically and edaphically shifted relative to the protologue frame, while lacking the molecular safeguards that would be uniquely warranted precisely because multiple entities are acknowledged. In this setting, epitypification does not stabilize; it increases the probability of lineage substitution, thereby weakening nomenclatural stability rather than strengthening it (Simonini et al. 2025: 29–30; Turland et al. 2025).

Finally, the net effect of the 2025 epitype selection is not merely that it “differs” from the neotype; it becomes incompatible with the established interpretative bridge that connects Fries’s untypified name to modern taxonomy. Fries (in Fries and Hök 1835) did not cite a preserved type, so continuity through protologue semantics and prevailing iconography has been unusually important for stable application (Fries and Hök 1835; Walty 1944, 1947, 1957, 1965; Singer 1967; Pilát and Dermek 1974; Bertolini and Simonini 2013). In that context, Simonini et al. (2025) motivated epitypification by re-framing the neotype-related plate as “now to be considered not sufficiently representative” (“ora da ritenere non sufficientemente rappresentativa”) and they explicitly invoked Art. 9.9, while simultaneously acknowledging that “at least two molecularly different species” (“almeno due specie molecolarmente differenti”) may fit the neotype iconography and referring to “investigazioni molecolari basate su ITS” (“ITS-based molecular investigations”) as background support (Simonini et al. 2025: 29–30). Yet the same paper stated that “This work is deliberately limited to morphological characters” (“Questo lavoro è volutamente limitato ai caratteri morfologici”), and it provided neither any microscopic characterization of the epitype (e.g., spore measurements or other diagnostic micromorphology) nor epitype-individual sequence data, nor any accessioned, traceable molecular outputs that would allow independent verification of how the illustrated collections relate to the designated epitype, MCVE18453 (Simonini et al. 2025: 30). At the same time, the claimed climatic comparability is not supported by the comparison of published Sauvabelin reconstructions for 1811–1847 with official Monticolo station data for 1977–2013, which shows markedly different August and annual thermal contexts and lower annual precipitation at the epitype proxy (Table 3; Imfeld et al. 2022, 2023; Imfeld and Brönnimann 2025, 2026; Provincia Autonoma di Bolzano [Meteo Alto Adige] n.d.). Alongside this, the asserted pedological equivalence is not supported by the regional soil-profile evidence, the official siliceous Bu2 description and the standardised SoilGrids median predictions (Walthert et al. 2003; Autonomous Province of Bolzano–South Tyrol n.d. [Bu2 type sheet]; Poggio et al. 2021). Coupled with the omission of epitype-level molecular anchoring in a multi-entity complex, the epitype does not “interpret” the neotype and protologue concept so much as compete with it. This outcome is contrary to the purpose of epitypification under Art. 9.9, and it is the central reason why the neotype-epitype discrepancy here is not a secondary taxonomic disagreement but rather a typification-level problem that threatens prevailing usage and interpretative stability.

The neotype illustration of *Boletus torosus* (Fig. 7f) depicts an extant species represented by documented modern collections in this study, including CNF 10/249 (Figs 2a–d, 3). Their correspondence with the neotype rests on the combination of morphological characters and the historical iconographic continuity discussed above, while the sequenced collections are recovered within the well-supported *Imperator torosus* s. str. clade (Fig. 1). The morphological comparison presented above shows that the epitype MCVE18453 does not conform to this character combination and represents a different species from that depicted by the neotype.

Confirmation that MCVE18453 belongs to *Imperator occultus* would establish that the selected epitype represents a species independently delimited in this study and distinct from *I. torosus* s. str., to which the neotype is referable on the morphological and historical evidence presented above. The characteristic yellow pileus ground colour and subsequent warm ochraceous to copper-brown discolouration of *I. occultus* would then be understood within the diagnostic character combination of that distinct species. Such a result would corroborate the taxonomic mismatch identified here: the selected epitype would represent a different species, rather than a colour variant of the species depicted by the neotype, and would therefore be taxonomically unsuitable to provide interpretative support for that neotype.

In the context of the distinct species recognised here, accepting this discordant epitype as decisive for name application would redirect *I. torosus* from the species represented by the neotype to another lineage. Such an application could displace the name applicable to that other species without resolving the nomenclature of the species represented by the neotype. Epitypification does not replace the neotype, which remains the nomenclatural type of *Boletus torosus* (Art. 8.1). Consequently, accepting the epitype-based application would not, by itself, render the species represented by the neotype unnamed. A new species name published for a circumscription that, at the time of publication, definitely included this neotype would be nomenclaturally superfluous and illegitimate under Art. 52.1–52.2, even if a different specimen were designated as its holotype. The conflict therefore cannot be resolved simply by accepting the epitype-based application and describing the neotype-containing species under a new name. Establishing precisely which other lineage the epitype belongs to would not restore its agreement with the neotype, nor does demonstrating their taxonomic disagreement by itself authorize disregarding the designated epitype. The discrepancy must therefore be addressed as a conflict within the typification of the name.

When an epitype and the type it purports to support are shown to differ taxonomically, and when the ordinary recourses for excluding or superseding a type do not readily apply (e.g. ICNafp Art. 9.18 for superseding neotypes; Art. 9.19 for types in serious conflict with the protologue), the Code provides a specific remedy. The name may be proposed for conservation with a conserved type (ICNafp Arts 9.20 and 14.9), unquestionably fixed by a route that leaves no room for further speculation. In the present case, the neotype-based application established in 2013 remains the closest available typification-level approximation to the protologue and the long-standing iconographic tradition, whereas the 2025 epitype demonstrably departs from that neotype-anchored framework and therefore cannot function as a subordinate clarificatory element under Art. 9.9. Conservation of the name with a new, independently verifiable conserved type (supported by documented morphology, microscopy, and accessioned sequences) would supersede the current typification and provide a single, reproducible reference point for the name (ICNafp Art. 14.9). Moreover, once the binding type is replaced by conservation, any epitype linked to the superseded typification has no standing with respect to the conserved type (ICNafp Art. 9.20, Note 11).

In practical terms, if conservation is approved under ICNafp Art. 14.9, the name *Boletus torosus* would be fixed to a conserved type representing the historically stabilised Fries/Secretan-neotype concept rather than the 2025 epitype concept, thereby superseding the 2013 neotype and, consequently, leaving the 2025 epitype without standing with respect to the replacement type (ICNafp Art. 9.20, Note 11). CNF 10/249, which we intend to designate as the conserved type in a separate nomenclatural proposal, is a fully documented fungarium specimen supported by photography, microscopy, and accessioned ITS, LSU, *tef*1, and *rpb*2 sequences. Together, CNF 10/249 and the additional sequenced collections recovered in the same lineage demonstrate that the same molecularly delimited species expresses the full diagnostic frame transmitted by the protologue and the established iconographic lineage: (i) the “basane livide” pileus baseline, leather-brown modulated by a livid/lead-grey cast (with its livid/lead-grey component already inherent in Secretan’s chromonym and reiterated in Fries 1838), as stabilised through the Walty-Singer-Pilát & Dermek depiction line formalised by the 2013 neotype, (ii) the protologue-linked pore ontogeny of pores that typically remain yellow but may occasionally develop reddish hues, particularly in aged basidiomes, (iii) Secretan’s emphasis on a “raboteux” pileus surface, including the protologue-described tessellation into brownish compartments with a yellow ground visible between fissures (documented in our Croatian *I. torosus* material in this study, not observed in any examined *I. occultus* collections, and not documented in the published epitype figure or associated illustrated material in Simonini et al. 2025), and (iv) a clearly expressed apical reticulum with dense meshes (“un réseau brun à mailles serrées…surtout dans le haut”), within the ecological framework of the protologue-defined humid beech-forest station at Sauvabelin (“sous les hêtres touffus, en lieu humide”), thereby maintaining prevailing usage in a manner consistent with the protologue and the neotype iconography (Secretan 1833; Fries and Hök 1835; Fries 1838; Walty 1944, 1947, 1957, 1965; Pilát and Dermek 1974; Bertolini and Simonini 2013; Simonini et al. 2025; this study). An isolated instance of earlier reddening is illustrated by a single Czech specimen (Fig. 2f).

### Reciprocal delimitation of *Imperator occultus* and *I. torosus*

*Imperator occultus* is a recognizable species based on the following set of morphological and ecological features: 1) large-sized, heavy basidiomes, 2) even pileus surface which is initially bright yellow to golden yellow but soon covered by a beige-greyish-greenish pruina over a warm yellow-ochraceous and finally copper orange to pale copper brown, 3) persistently golden yellow to sometimes orange or red pores since the early stage of development, 4) yellow stipe ornamented by a well-developed reticulum with isodiametric meshes, 5) strongly cyanescent tissues upon bruising or cutting, 6) pleasant, vaguely fruity odour turning into a disagreeable, medicine-like smell with age, 7) mild taste, 8) yellowish brown spore print, 9) smooth, ellipsoid–fusiform basidiospores (9.7) 13.5 ± 1.0 (16.0) × (4.9) 5.9 ± 0.3 (6.9) μm, Qm = 2.29, 10) occasional presence of hymenial pseudocystidia with a bright honey yellow content, 11) ixotrichoderm to ixocutis pileipellis, 12) occurrence preferably in warm broadleaved woodlands in hilly environments, mainly under *Quercus* spp. and *F. sylvatica* but also under conifers (*Abies* spp.) at higher elevations (in Southern Europe), on calcareous soil.

*Imperator torosus* and *I. occultus* are lookalike species with a surprisingly similar outward appearance, and extra care is required for rigorous character delimitation. *Imperator occultus* differs from *I. torosus* macroscopically by a typically whitish-cream then yellow-ochraceous to pale brown pileus. In *I. torosus*, the baseline pileus palette is usually a grey-cast leather-brown, i.e. leaden-grey to leaden-brown, sometimes with faintly to dark olivaceous tinges, with yellow generally remaining a subordinate underlying tone that may locally re-emerge where the surface pruina is thin, cracked or absent. In *I. occultus*, the light greenish hue is almost certainly the resulting mixture of grey and yellow tints which produces an impression of “false” green. That said, the pileus colour in *I. torosus* and *I. occultus* is so different that even accepting the presence of “phantom” pale greenish hues in *I. occultus* it would not determine a risk of colour overlapping between these two species. *Imperator occultus* further differs by pores that can already show distinct red to red-orange hues in young specimens and typically retain clearly expressed red tones through maturity (in *I. torosus*, pores typically remain yellow). Occasionally, particularly in aged basidiomes and in humid weather, reddish hues may develop at varying intensities, ranging from orange-red to red and shading to yellow-orange or orange towards the pileus margin. In addition, a careful examination of pore size reveals that in *I. torosus* the pores, even at extreme maturity, do not exceed 1 mm in diameter, whereas in *I. occultus* the pores at maturity are at least twice as large, reaching approximately 2 mm in diameter. *Imperator occultus* also exhibits a reticulum on the stipe that is less prominent, more elongated and more regularly arranged. In contrast, in *I. torosus*, the stipe reticulum is more strongly developed and conspicuously raised, with denser and sometimes irregularly anastomosing meshes that are especially evident in the upper part, in agreement with Secretan’s protologue (“Un réseau brun à mailles serrées est apparent surtout dans le haut”).

Microscopically, *I. occultus* is set apart from *I. torosus* by the shorter (on average 13.5 μm long in *I. occultus* vs. 13.8 μm long in *I. torosus*) and slightly wider (on average 5.9 μm broad in *I. occultus* vs. 5.6 μm broad in *I. torosus*) basidiospores with a different Q ratio (Qm 2.3 in *I. occultus* vs. 2.5 in *I. torosus*) and the occasional presence of mostly cylindrical to subclavate pseudocystidia with a bright yellow intracellular pigment (this feature must be checked on a larger number of collections to better weight its taxonomic relevance), which have never been observed in *I. torosus*. Ecologically, *I. occultus* occurs in thermophilic habitats at relatively low altitudes (lowland to hilly environments often with the presence of *Quercus* spp. and *F. sylvatica*), although in southern Europe it can also grow in pedemontane or montane environments up to 1300 m a.s.l., in pure stands of *Pinaceae* (*Abies* spp.), including conifer plantations, whereas *I. torosus* prefers cooler and more continental habitats at higher altitudes (often with the presence of *A. alba*, *P. abies* and *F. sylvatica*). Apparently, *I. occultus* may associate with a broader range of host trees than *I. torosus*.

All these differences may appear of little significance, but they are stable and robustly supported by molecular phylogenetic inference, thus making the separation of *I. torosus* and *I. occultus* satisfactory from the morphological, ecological and genetic standpoints. ITS-LSU phylogenetic analysis clearly indicates that *I. torosus* and *I. occultus* form two independent terminal clades which are recovered by strong statistical support (PP = 1.00, BS = 99%).

Soil pH was assessed by one of us (NF) at various Croatian localities where *I. torosus* and *I. occultus* co-occur. In all cases, the pH ranged between 6.0 and 6.5, indicating a soil reaction close to neutral. Therefore, soil pH cannot be used as a reliable ecological feature to discriminate between these two taxa. Furthermore, neither *I. torosus* nor *I. occultus* appear to be specialised as far as their associated host trees are concerned, since they can establish ectomycorrhizal (EcM) relationships with a large array of woody plants.

Despite our limited knowledge of its geographic range, the available molecularly confirmed records indicate that *I. occultus* is a thermophilic species occurring in southern, central and western Europe, with a preference for calcareous soils. In addition, it seems to be associated preferably with hardwood hosts, especially deciduous oaks (*Quercus* spp.) and *F. sylvatica* but also with other frondose trees and more rarely with conifers. However, as reported above it appears evident that in some intergrading areas the geographic range of *I. torosus* and that of *I. occultus* are complementary to some extent and do not seem to be exclusively confined to any specific geographic region. Future collections from different sites across southern Mediterranean countries and temperate Europe will be necessary to assess EcM host specificity and to extend the distribution range of *I. occultus*. Equally, additional investigation on a larger scale will be required to delimit the exact edaphic requirements, associated vegetation and spatial distribution of *I. torosus* s. str. in Europe and abroad.

We suspect that the image of *Boletus appendiculatus* Schaeff. offered in Brillouet (1987), Brillouet et al. (1989) and the pictures of *B. torosus* published by Galli (1987, 1996, 1998, 2007, 2013, 2023) might indeed represent specimens of the newly introduced *I. occultus*, but regrettably we are unable to ascertain its identity due to the absence of voucher collections for comparison. Similarly, *B. junquilleus* (Quél.) Boud. in Angarano (1988) might be *I. occultus*. In addition, we have good reasons to believe that *B. torosus* s. Muñoz (2005) corresponds to *I. occultus*, but even in this case we cannot confirm it with certainty. The inclusion of a sequence in our dataset derived from an Italian collection kindly provided by J.A. Muñoz which nests within the *I. occultus* clade would reinforce this hypothesis. Again, the red-pored specimens of *B. torosus* reported by Dermek (1979) and by Sarkina (2020b) from Slovakia and Crimea, respectively, might well be *I. occultus*, as well as *I. occultus* could be the predominantly yellow-coloured specimens named as *I. torosus* by Wuilbaut (2011), the specimens named *B. lupinus* Fr. by Konstantinidis (2006) and the basidiomes of *B. xanthocyaneus* drawn by Roth (2002). *Boletus torosus* as reported by Karadelev et al. (2007, 2018) from Macedonia might as well represent *I. occultus*. Many other accessions, especially in southern European literature, listed under the epithet “*torosus*” or “*B. torosus* var. *daniaudii*” are presumed to be referred to *I. occultus* (see Table 1: C for a full list of references), but lacking supporting evidence we presently refrain from assigning these records with certainty to our novel species. *Imperator* sp. in Mikšík and Polčák (2024) and *Imperator cf. torosus* as reported in Mikšík et al. (2025) obviously refer to *I. occultus*.

### Critical evaluation of *Boletus xanthocyaneus* and the taxonomic status of *Imperator rhodopurpureus* f. xanthopurpureus

The existence of a novel autonomous species in the *I. torosus* species complex raises the question of the correct taxonomic position of the yellow-coloured *I. xanthocyaneus* (Ramain ex Romagnesi) Klofac. A careful evaluation of the original description of *B. purpureus* subsp. *xanthocyaneus* Ramain (Ramain 1948) allows us to fix some relevant morphological features related to the pileal surface, which was reported to be “bumpy…and granulose-rugose, not smooth, nor truly velvety…somewhat spotted in places with cherry-red-purple…” (“…± bosselé…et granuleux-ruguleux, non lisse, ni vraiment velouté…± tacheté par places de rouge-cerise-purpurin…”), and to pore colour at maturity: “gradually becoming (at maturity) vermilion-purple, especially around the stipe” (“…devenant peu à peu (dans l’âge mûr) vermillon-purpurin, surtout autour du pied”), thus matching the modern concept of a xanthoid form of *I. rhodopurpureus*, namely *I. rhodopurpureus* f. xanthopurpureus (Smotl.) Mikšík. In addition, the persistence of the yellow tint on the pileus surface over the entire life cycle in *B. purpureus* subsp. *xanthocyaneus* as well as the occurrence of hymenial cystidia with a yellow content would point towards a further, apparently undisputed identity of this taxon with *I. rhodopurpureus* f. xanthopurpureus, as pointed out by Muñoz (2005). Ramain's (1948) name *B. purpureus* subsp. *xanthocyaneus* was not validly published because no Latin description or diagnosis was provided (Art. 39.1). In addition, he did not provide any iconography for this taxon.

The interpretation of *B. purpureus* subsp. *xanthocyaneus* by Romagnesi (1976) is somewhat confused and the French author eventually raised this taxon to species rank, validating it by providing a short Latin diagnosis and a holotype voucher specimen located in the forest of Orry-la-Ville (Île-de-France) (preserved in the fungarium of the laboratoire de cryptogamie du Muséum National d’Histoire Naturelle de Paris, MNHN), far away from the original collecting site cited by Ramain, and again the iconography was missing, although Romagnesi indicated a colour plate in Leclair and Essette (1969) under the name *B. torosus* as a representative illustration of *B. xanthocyaneus* (Romagnesi 1976). Such a plate, however, is most likely a mixture between *B. xanthocyaneus* and the true *I. torosus*. As for *B. rhodopurpureus*, which was introduced (along with its chromatic forms) from the former Czechoslovakia more than twenty years earlier (Smotlacha 1952), Romagnesi believed it to be nothing more than a chromatic variant of his “*B. purpureus*” growing in wet weather conditions (Romagnesi 1976). However, *B. purpureus* s. Romagnesi as circumscribed in his dichotomous key (Romagnesi 1976) is almost certainly a collective species with an initial “gris brunnâtre ou café au lait” tint of the pileal surface, which is much reminiscent of a young stage of *Rubroboletus legaliae* (Pilát & Dermek) Della Magg. & Trassin. or *R. rubrosanguineus*, whereas the colours becoming “dark brown…or dark purple-red” (“brun bistre…ou rouge pourpre sombre”) in aged specimens appear to be closer, at least in part, to those observed in *I. rhodopurpureus*. On the other hand, *Boletus torosus* var. *gallicus* Romagnesi is described as a red-pored bolete with a pale yellow pileus, staining dark blue at the slightest touch and later becoming reddish (Romagnesi 1976), which corresponds fairly well to the modern concept of *I. luteocupreus*, although Redeuilh (1992a) was inclined to consider this taxon a simple chromatic variant falling into the infraspecific variability of *I. rhodopurpureus*, and thus recombined it as a variety of the latter species (Redeuilh 1992b).

In 1983, the Italian mycologist B. Cetto offered a picture of “*B. purpureus* var. *xanthocyaneus*” which is evidently best referred to as *I. luteocupreus* due to the evenly dark garnet red pores (Cetto 1983). Brillouet (1987), Brillouet et al. (1989) and Brillouet and Brillouet (1993) were the first authors after Romagnesi to consider *B. xanthocyaneus* as a species standing on its own and were also the first to provide colour photographs of this taxon. They stressed the persisting yellow colour of the uneven pileus surface without grey or green hues but frequently with reddish spots scattered here and there at maturity, the stipe generally devoid of red tints or, if present, confined to the basal part, and the occurrence on siliceous-acidic soil as the most important morphological and ecological features for distinguishing *B. xanthocyaneus* from *B. torosus* (Brillouet and Brillouet 1993). Simonini (1995, 1996) emphasized the fruity odour of *B. xanthocyaneus* as an additional difference to separate it from *B. torosus* but argued, similarly to Cazzoli (1991), that *B. xanthocyaneus* was an alleged chromatic variant of *B. rhodopurpureus*.

Alessio (1998) published a bizarre colour plate of *B. xanthocyaneus* made by E. Rebaudengo with various specimens in different developmental stages all displaying a large, prominent basal bulb, which is abruptly emarginate and separated from the upper part of the stipe by a ring-like zone. The author concluded, in concordance with G. Simonini, that such a feature might represent an occasional and aberrant phenomenon owing to exceptional growth conditions or to other unknown environmental factors. The related description of *B. xanthocyaneus* (Alessio 1998) is also somewhat ambiguous. In the same year Simonini (1998) finally interpreted *B. xanthocyaneus* as nothing more than a simple xanthoid form of *B. rhodopurpureus*. On the contrary, Hills (1997), Lucchini (1997), Bobinet and Dupuy (1998) and Lannoy and Estadès (2001) treated *B. xanthocyaneus* as an independent species.

Estadès and Deïana (2004) proposed an extremely detailed comparison between *B. xanthocyaneus* and *B. torosus*, the former species having been found with several basidiomes at the original site of Chablais (topotype specimens based on the invalid description provided by Ramain!). They strengthened the differences between the two taxa as already stressed by Redeuilh (1992a, 1992b), Brillouet and Brillouet (1993) and Simonini (1995, 1998) by underlining for *B. xanthocyaneus* a lower specific weight (0.6 kg/dm^3^ for *B. xanthocyaneus* vs. 0.7–0.8 kg/dm^3^ for *B. torosus*), slightly smaller basidiospores [(11) 11.5–14.5 (15) × (4.5) 5–5.5 (6) μm, on average 12.6 × 5.3 μm, Qm = 2.4 in *B. xanthocyaneus* based on the re-examination of the holotype material vs. (12) 13–15.5 (17.5) × 5–6.5 (7) μm, on average 14.2 × 5.7 μm, Qm = 2.5 in *B. torosus*], and a growth in lowland or hilly environments under various frondose trees on clayey-siliceous, acidic soil for *B. xanthocyaneus* (preference for pedemontane to montane environments under *F. sylvatica* frequently mixed with *A. alba* and *P. abies* or exceptionally in pure stands of *Abies alba* on basic-calcareous soil, up to an altitude of 1300 m for *B. torosus*) (Estadès and Deïana 2004). They also agreed with previous authors in retaining *B. xanthocyaneus* in close proximity to *B. rhodopurpureus* but considered the identity of *B. xanthocyaneus* and *B. rhodopurpureus* var. xanthopurpureus as doubtful: “the synonymy of *B. rhodopurpureus* f. xanthopurpureus with *B. xanthocyaneus* seems to be difficult to admit for the time being, because this latter is very stable in the warm sites where we find it” (“La synonymie de *B. rhodopurpureus* f. xanthopurpureus avec *B. xanthocyaneus*, paraît difficile à admettre pour l’instant, puisque ce dernier est très stable dans nos stations chaudes”) (Estadès and Deïana 2004: 20). Such an interpretation of *B. torosus* and *B. xanthocyaneus* as separate and autonomous taxa was later followed by several authors (Estadès and Lannoy 2004; Watling and Hills 2005; Evans et al. 2006; Calzada Domínguez 2007; Courtecuisse and Duhem 2007; Hills 2007; Klofac 2007, 2011; Moingeon and Moingeon 2005, 2007; Gelardi 2008a; Boccardo et al. 2008; Kibby 2011, 2017; Wuilbaut 2011; Ainsworth et al. 2013; Reumaux and Carteret 2013; Šikić 2013; Vasquez 2014; Klofac and Krisai-Greilhuber 2020) but the real taxonomic placement of *B. xanthocyaneus*, especially with respect to depigmented phenotypes of *I. rhodopurpureus*, has long remained obscure (Simonini and Bertolini 2012).

Most recently, the scientific community reached a consensus on the identity of *B. xanthocyaneus* as a simple xanthoid form of *I. rhodopurpureus*, with the tendency to consider the former contaxic with *I. rhodopurpureus* f. xanthopurpureus (Muñoz 2005; Galli 2007, 2013; Mikšík et al. 2025), as firstly surmised by Redeuilh (1992b), especially assuming this is the morphotype to which the protological concept of *B. xanthocyaneus* fits best. The basidiospore width of a recently yielded, molecularly confirmed collection of *I. rhodopurpureus* (CNF 10/322) from Croatia (on average 12.0 × 5.2 μm, Qm = 2.3) appears to be overlapping with that of the holotype of *B. xanthocyaneus* as reported above, further reinforcing their conspecificity. For an updated list of bibliographic and iconographic accounts on *I. xanthocyaneus*, see Simonini and Bertolini (2012).

*Imperator rhodopurpureus* f. xanthopurpureus (Fig. 9) differs from *I. occultus* by a rugulose pileus surface with a very irregular, undulate margin exceeding pores underneath and with a persistent lemon yellow, sulfur yellow to light greenish yellow tint, shorter tubes at maturity, usually up to 1.5 cm long (or at most 2 cm long), yellow pores becoming reddish starting from around the stipe in aged specimens, slightly narrower basidiospores (5.6 µm wide on average based on our measurements, 5.2 µm according to Muñoz 2005, 5.3 µm according to the re-examination of the holotype material of *I. xanthocyaneus* by Estadès and Deïana 2004) and the occurrence on acidic soil (Smotlacha 1952; Hlaváček 1986; Redeuilh 1992a; Simonini 1995, 1998; Hills 1997; Cazzoli and Consiglio 2001; Estadès and Deïana 2004; Estadès and Lannoy 2004; Muñoz 2005; Röger 2007; Šutara et al. 2009; Simonini and Bertolini 2012; Galli 2013; Mikšík et al. 2025). Several recent collections identified as *B. xanthocyaneus* by experienced mycologists from France following Romagnesi’s tradition were all confirmed as *I. rhodopurpureus* by ITS sequences.

Unfortunately, despite several attempts to sequence the holotype of *B. xanthocyaneus* (currently preserved in the Fungarium of the National Museum of Natural History, Paris, France, PC 0798992), using a set of the most frequently used sequencing technologies currently available (Sanger, Oxford Nanopore, and Illumina), the very poor condition of the type collection prevented us from generating useful genetic material.

### Morphological comparison with *Cyanoboletus poikilochromus*

The thermophilic, strictly Mediterranean species *Cyanoboletus poikilochromus* (Pöder, Cetto & Zuccher.) M. Carbone, D. Puddu & P. Alvarado is well recognisable and easily discriminated from *I. occultus* by the comparatively less robust basidiomes with a smaller size (pileus 4.6–12.2 cm but sometimes up to 13–15 cm diam.) and lighter specific weight, yellow pores and tubes which tend to become copper-orange spontaneously with age, stipe base moderately to deeply rooting, stipe reticulum more pronounced and with elongated meshes, a conspicuous, strong and persistent sweetish odour recalling that of fermented fruit or reminiscent of propolis, liquor, dried cinnamon or cottonwood (which also persists and intensifies in dried material), olive-brown spore print, formation of tiny, glassy acicular crystal concretions upon drying (visible under a stereomicroscope and SEM) resulting from the presence of a yellow substance exuding from the hymenophore, smaller basidiospores measuring (11.0) 11.8 ± 0.7 (12.4) × (4.5) 5.0 ± 0.3 (5.4) μm, Qm = 2.36 ± 0.16, V = 154 μm^3^, slightly longer cheilocystidia [(22) 32–63 (68) × 7–10 μm] and pleurocystidia [(28) 33–61 (70) × 7–13 μm], larger and numerous gloeocystidia 38–92 × 7–19 μm which are moderately dextrinoid and staining almost blackish in brilliant cresyl blue, longer caulobasidia 37–90 × 9–13 μm and a trichoderm pileipellis (Pöder 1987; Simonini et al. 1988; Migliozzi and Coccia 1991; Simonini 1995, 1998; Migliozzi and Camboni 1999; Calonge and Redeuilh 2000; Lunghini and Perrone 2002; Muñoz 2005; Redeuilh and Alpago Novello 2005; Simonini and Bertolini 2012; Galli 2013; Gelardi et al. 2015; Biketova et al. 2016, 2026; Mikšík et al. 2025; this study).

### Conservation status

As far as the conservation status is concerned, *I. torosus* is generally considered a rare species, which is included in the red lists of Austria (Dämon et al. 2023) as “vulnerable”, Bulgaria as “data deficient” (Gyosheva et al. 2006; Assyov and Denchev 2010), Croatia (Tkalčec et al. 2008) as “vulnerable”, Czechia (Zíbarová et al. 2024) as “critically endangered”, Germany (Zehfuß et al. 2000; Karasch and Hahn 2009) as “endangered”, France (Corriol 2014; UICN Comité français et al. 2024) as “endangered”, Montenegro as a protected species (Kasom and Miličković 2010), Romania (Anghelescu and Costa 2023) as “vulnerable/rare”, Slovakia (Kautmanová 2008) as “vulnerable”, Slovenia (Logar 2012) as “rare”, Switzerland (Senn-Irlet et al. 1997, 2007) as “endangered”, as well as in that of the United Kingdom as “data deficient” (Ainsworth et al. 2013) (in the UK this species was previously assessed as “vulnerable” by Evans et al. 2006 but authentic records are lacking). In Russia, it is included in the Red Data Book of the Republic of Adygea as “endangered in critical condition” (1A) (Kiyashko 2023) and it is protected in the Caucasian State Reserve (Vaasma et al. 1986). It has also been assessed as Endangered (EN) in a regional red-list study of *Boletales* in central Italy (Wagensommer et al. 2021). On the other hand, because of its local abundance *I. torosus* has recently been listed in the category “Least Concern (LC)” in the Auvergne-Rhône-Alpes red list (Van Vooren et al. 2023). Further studies of distribution and population structure of both *I. torosus* and *I. occultus* are needed to evaluate protection status regionally and globally and to apply necessary protection measures.

## Conclusion

In the present work, an ITS-LSU phylogenetic reconstruction, along with PD and PNS analyses, allows us to redefine the *I. torosus* species concept. In addition, molecular analysis and careful morphological study unquestionably indicate *I. torosus* as a species complex and infer the recognition of a hitherto undescribed cryptic taxon. *Imperator occultus* is therefore introduced as a novel species. The critical species *I. xanthocyaneus* is morphologically assessed, although the holotype was not molecularly characterized. Based on acquired evidence, all genetically proven collections initially identified as *I. xanthocyaneus* are confirmed as yellow-coloured aspects of *I. rhodopurpureus*, namely *I. rhodopurpureus* f. xanthopurpureus, by DNA barcoding and phylogenetic analysis.

## Identification key to the known species of *Imperator*

**Table:** 

1	Pileus yellowish or yellow-ochraceous then pale brown or leaden-grey to leaden-brown sometimes with olivaceous tinges; pores deep yellow, sometimes orange to red at different developmental stages; odour medicines-like, unpleasant; growing in ECM association with both conifers and broadleaved trees	**2**
–	Pileus and pores with a different combination of colours; odour fruity and agreeable; growing in ECM association with broadleaved trees only	**3**
2	Pileus leaden-grey to leaden-brown over a yellow background, sometimes with olivaceous tinges; pores typically remaining yellow, rarely developing reddish hues, particularly in aged basidiomes, smaller at maturity (not exceeding 1 mm in diameter); stipe with a more prominent, irregularly arranged reticulum with denser meshes; basidiospores longer and slightly narrower (13.8 × 5.6 μm) with a Q ratio 2.5; occurrence in mesophilic, continental habitats, generally at higher altitudes, with *A. alba*, *F. sylvatica* and *P. abies*	***Imperator torosus***
–	Pileus whitish-cream then yellow-ochraceous with scattered reddish-purple areas to pale brown; pores yellow, sometimes red to red-orange already in young specimens, larger at maturity (up to 2 mm in diameter); stipe with a less prominent, more elongated and more regularly arranged reticulum; basidiospores shorter and slightly wider (13.5 × 5.9 μm) with a Q ratio 2.3; occurrence in more thermophilic habitats, mainly with *Quercus* spp. and *F. sylvatica*, less frequently with conifers (*Abies* spp.)	***Imperator occultus***
3	Pileus persistently yellow over the entire life cycle; pores yellow, sometimes developing orange to reddish hues in aged basidiomes, beginning around the stipe; pileus rugulose	***Imperator rhodopurpureus* f. xanthopurpureus** (= ***I. xanthocyaneus***)
–	Pileus initially yellow or pinkish yellow then reddish orange, copper red to reddish brown at maturity or bright pink to purplish red since the beginning, then dirty gray or gray-brown at maturity, pileus rugulose or smooth, pores orange-red to blood red, sometimes initially yellow	**4**
4	Pileus smooth, initially yellow or pinkish yellow then reddish orange, copper red to reddish brown at maturity; stipe orange since the beginning, covered with a prominent and well-developed red reticulum	***Imperator luteocupreus***
–	Pileus rugulose, bright pink to purplish red since the beginning, then dirty grey or grey-brown at maturity; stipe yellowish or purplish red since the beginning, covered with a fine reticulum usually restricted to the upper half	**5**
5	Pileus bright pink to purplish red; pores initially yellow but soon red-orange to deep red; stipe yellowish at first	***Imperator rhodopurpureus* var. *rhodopurpureus* f. *rhodopurpureus***
–	Whole fruiting body (pileus, pores and stipe) deep crimson red to purple red from the beginning	***Imperator rhodopurpureus* var. *polypurpureus***

## Supplementary Material

XML Treatment for Imperator torosus

XML Treatment for Imperator occultus

## Additional information

### Artificial Intelligence (AI) use

The authors accept full responsibility for the content of the manuscript, including the disclosure of any use of AI.

No AI tools were used in the preparation of this manuscript.

### Author ORCIDs

M. Gelardi https://orcid.org/0009-0008-0903-852X

N. Ferenčak https://orcid.org/0009-0000-1644-8641

A.Yu. Biketova https://orcid.org/0000-0003-0801-0660

M. Požnjak https://orcid.org/0009-0002-7296-598X

A.D. Treindl https://orcid.org/0000-0001-7347-2891

A. Gross https://orcid.org/0000-0002-2489-8105

G. Saar https://orcid.org/0000-0002-6365-7929

M. Cartabia https://orcid.org/0000-0002-1986-3178

L. Albert https://orcid.org/0000-0003-4505-3010

M. Mikšík https://orcid.org/0000-0003-4415-7184

E. Zupan https://orcid.org/0009-0008-2595-1888

P.-A. Moreau https://orcid.org/0000-0003-4783-3643

J.-M. Bellanger https://orcid.org/0000-0001-9289-2349

B. Assyov https://orcid.org/0000-0002-7365-2443

B. Dima https://orcid.org/0000-0003-2099-3903

A. Vizzini https://orcid.org/0000-0001-8390-6446

### Data availability

All of the data that support the findings of this study are available in the main text or Supplementary Information.

## Supplementary materials

Supplementary material 1 Information on specimens Data type docx Explanation note **table SS1**. Information on specimens used in phylogenetic analysis and their accession numbers. https://binary.pensoft.net/file/1826842This dataset is made available under the Open Database License (http://opendatacommons.org/licenses/odbl/1.0/). The Open Database License (ODbL) is a license agreement intended to allow users to freely share, modify, and use this Dataset while maintaining this same freedom for others, provided that the original source and author(s) are credited. Matteo Gelardi, Neven Ferenčak, Alona Yu. Biketova, Marko Požnjak, Artemis Diana Treindl, Andrin Gross, Günter Saar, Marco Cartabia, Jacques Guinberteau, László Albert, Michal Mikšík, Eva Zupan, Pierre-Arthur Moreau, Jean-Michel Bellanger, Boris Assyov, Bálint Dima, Alfredo Vizzini

Supplementary material 2 Complete depth-specific values and monthly values Data type docx Explanation note **table SS2**. SoilGrids v2.0 median (Q0.5) point predictions for pH in water (phh2o) and coarse-fragment content (cfvo) at the four targeted localities. **table SS3**. Monthly and annual climatic context of historical Sauvabelin (1811–1847) and Monticolo/Montiggl (1977–2013), the observational proxy for the Castelvecchio epitype locality. https://binary.pensoft.net/file/1826843This dataset is made available under the Open Database License (http://opendatacommons.org/licenses/odbl/1.0/). The Open Database License (ODbL) is a license agreement intended to allow users to freely share, modify, and use this Dataset while maintaining this same freedom for others, provided that the original source and author(s) are credited. Matteo Gelardi, Neven Ferenčak, Alona Yu. Biketova, Marko Požnjak, Artemis Diana Treindl, Andrin Gross, Günter Saar, Marco Cartabia, Jacques Guinberteau, László Albert, Michal Mikšík, Eva Zupan, Pierre-Arthur Moreau, Jean-Michel Bellanger, Boris Assyov, Bálint Dima, Alfredo Vizzini

Supplementary material 3 ITS-LSU alignment Data type fas Explanation note Concatenated ITS–LSU alignment of 94 specimens, comprising 1,958 aligned positions including gaps, used for the phylogenetic analyses presented in Fig. 1. https://binary.pensoft.net/file/1826844This dataset is made available under the Open Database License (http://opendatacommons.org/licenses/odbl/1.0/). The Open Database License (ODbL) is a license agreement intended to allow users to freely share, modify, and use this Dataset while maintaining this same freedom for others, provided that the original source and author(s) are credited. Matteo Gelardi, Neven Ferenčak, Alona Yu. Biketova, Marko Požnjak, Artemis Diana Treindl, Andrin Gross, Günter Saar, Marco Cartabia, Jacques Guinberteau, László Albert, Michal Mikšík, Eva Zupan, Pierre-Arthur Moreau, Jean-Michel Bellanger, Boris Assyov, Bálint Dima, Alfredo Vizzini
