# Supplementary material for: An integrated taxonomic approach refines the Imperator torosus species concept and reveals the cryptic I. occultus sp. nov. (Boletales, Boletaceae)
Source: MycoKeys. 2026 Nov 2;142:335–413. doi: 10.3897/mycokeys.142.208955 (PMC13749066; doi:10.3897/mycokeys.142.208955)
Supplement: Supplementary file 1 [file mycokeys-142-335-s001.docx]

**Table S1.** Information on specimens used in phylogenetic analysis and their accession numbers. Newly generated sequences are in boldface. Sequences that were used in PNS and PD analyses, but not included in phylogeny are marked with an asterisk (*).

| **Species** | **Voucher** | **Geographic origin** | **GenBank and UNITE accession numbers** | | | | **Notes** |
| --- | --- | --- | --- | --- | --- | --- | --- |
|  |  |  | **ITS** | **LSU** | ***tef1*** | ***rpb2*** |  |
| ***Cyanoboletus poikilochromus*** | **CNF 10/301** | **Croatia: Šibenik-Knin co.** | **QB013754** | **-** | **-** | **-** | **-** |
| *Cyanoboletus poikilochromus* | IB19960585 | Italy: Emilia-Romagna | PZ244176 | PZ231929 | - | - | Paratype |
| ***Imperator luteocupreus*** | **ZT Myc 0094436** | **Switzerland: Ticino Canton** | **PZ973661** | | **-** | **-** | **-** |
| *Imperator luteocupreus* | ML110112BL | Cyprus: Limassol district | MH011871 | - | - | - | **-** |
| *Imperator luteocupreus* | ML01811RS | Cyprus: Paphos district | MH011873 | - | - | - | **-** |
| *Imperator luteocupreus* | ML211101BL | Cyprus: Paphos district | MH011872 | - | - | - | **-** |
| *Imperator luteocupreus* | PRM924419 | Czechia: South Moravian region | LT223153 | - | - | - | **-** |
| *Imperator luteocupreus* | FR2011127 | France: Occitanie | MH011867 | - | - | - | **-** |
| *Imperator luteocupreus* | GS1220 (17750) | Italy | JF907790 | - | - | - | as *Imperator rhodopurpureus* in GenBank |
| *Imperator luteocupreus* | GS2127 (18431) | Italy | JF907796 | - | - | - | as *Imperator rhodopurpureus* in GenBank |
| *Imperator luteocupreus* | TUF143025 | Italy: Lazio | UDB07676781 | - | - | - | **-** |
| *Imperator luteocupreus* | TUF143032 | Italy: Lazio | UDB07676785 | - | - | - | **-** |
| *Imperator luteocupreus* | LUGO:ECC18071805 | Spain: Galicia | MW376670 | - | - | - | **-** |
| *Imperator luteocupreus* | JAD24102805 | Spain: Galicia | PZ201851 | - | - | - | **-** |
| ***Imperator occultus*** | **K-M001450423 (AB B18-290)** | **Croatia: City of Zagreb** | **QB013768** | **-** | **-** | **-** | **Paratype** |
| ***Imperator occultus*** | **K-M001450422 (AB B18-291)** | **Croatia: City of Zagreb** | **QB013767** | **-** | **-** | **-** | **Paratype** |
| ***Imperator occultus*** | **K-M001450420 (AB B18-292)** | **Croatia: City of Zagreb** | **QB013766** | **-** | **-** | **-** | **Paratype** |
| ***Imperator occultus*** | **CNF 10/247** | **Croatia: City of Zagreb** | **QB013757** | **-** | **-** | **-** | **Paratype** |
| ***Imperator occultus*** | **CNF 10/319** | **Croatia: City of Zagreb** | **QB013758** | **-** | **-** | **-** | **Paratype** |
| ***Imperator occultus*** | **CNF 10/34** | **Croatia: Varaždin co.** | **PZ973662** | | **PZ973364*** | **PZ973366*** | **Holotype** |
| ***Imperator occultus*** | **CNF 10/131** | **Croatia: Varaždin co.** | **QB013755** | **-** | **-** | **-** | **Paratype** |
| ***Imperator occultus*** | **CNF 10/132** | **Croatia: Varaždin co.** | **QB013756** | **-** | **-** | **-** | **Paratype** |
| ***Imperator occultus*** | **CNF 10/325** | **Croatia: Zagreb co.** | **QB013759** | **-** | **-** | **-** | **Paratype** |
| ***Imperator occultus*** | **CNF 10/326** | **Croatia: Zagreb co.** | **QB013760** | **-** | **-** | **-** | **Paratype** |
| ***Imperator occultus*** | **FR2024497** | **France: Île-de-France** | **QB013762** | **-** | **-** | **-** | **Paratype** |
| ***Imperator occultus*** | **JG2108011** | **France: Provence-Alpes-Côte d'Azur** | **QB013763** | **-** | **-** | **-** | **Paratype** |
| ***Imperator occultus*** | **GS-2018-12** | **Germany: Baden-Württemberg** | **QB013774** | **-** | **-** | **-** | **Paratype** |
| ***Imperator occultus*** | **GS-2018-13** | **Germany: Baden-Württemberg** | **QB013775** | **-** | **-** | **-** | **Paratype** |
| ***Imperator occultus*** | **GS-25-16** | **Germany: Baden-Württemberg** | **QB013773** | **-** | **-** | **-** | **Paratype** |
| ***Imperator occultus*** | **PRM965781** | **Italy: Emilia-Romagna** | **QB013784** | **-** | **-** | **-** | **Paratype** |
| ***Imperator occultus*** | **PRM965782** | **Italy: Emilia-Romagna** | **QB013785** | **-** | **-** | **-** | **Paratype** |
| ***Imperator occultus*** | **MC20230919.1** | **Italy: Lombardy** | **QB013770** | **-** | **-** | **-** | **Paratype** |
| ***Imperator occultus*** | **MG1026** | **Italy: Marche** | **QB013776** | **-** | **-** | **-** | **Paratype** |
| ***Imperator occultus*** | **MG1027** | **Italy: Marche** | **QB013777** | **-** | **-** | **-** | **Paratype** |
| ***Imperator occultus*** | **MG1028** | **Italy: Marche** | **QB013778** | **-** | **-** | **-** | **Paratype** |
| ***Imperator occultus*** | **CR-14-08-2016** | **Italy: Trentino-Alto Adige** | **QB013761** | **-** | **-** | **-** | **Paratype** |
| ***Imperator occultus*** | **MG1034-1 (FRJ010-2017)** | **Italy: Trentino-Alto Adige** | **QB013779** | **-** | **-** | **-** | **Paratype** |
| ***Imperator occultus*** | **MG1034-2** | **Italy: Trentino-Alto Adige** | **QB013780** | **-** | **-** | **-** | **Paratype** |
| ***Imperator occultus*** | **MG1035 (FRJ106-2018)** | **Italy: Trentino-Alto Adige** | **QB013781** | **-** | **-** | **-** | **Paratype** |
| ***Imperator occultus*** | **PRM965780** | **Italy: Trentino-Alto Adige** | **QB013783** | **-** | **-** | **-** | **Paratype** |
| ***Imperator occultus*** | **LJF8893** | **Slovenia: Kočevje admin. unit** | **QB013769** | **PZ979656** | **-** | **-** | **Paratype** |
| ***Imperator occultus*** | **PRM965779** | **Spain: Catalonia** | **QB013782** | **-** | **-** | **-** | **Paratype** |
| ***Imperator occultus*** | **JS 02/18** | **Switzerland: Basel-Landschaft canton** | **QB013764** | **-** | **-** | **-** | **Paratype** |
| ***Imperator occultus*** | **JS 03/18** | **Switzerland: Basel-Landschaft canton** | **QB013765** | **-** | **-** | **-** | **Paratype** |
| ***Imperator occultus*** | **ZT Myc 0094430** | **Switzerland: Basel-Landschaft canton** | **QB013771** | **PZ979657** | **-** | **-** | **Paratype** |
| ***Imperator occultus*** | **ZT Myc 0094437** | **Switzerland: Basel-Landschaft canton** | **QB013772** | **-** | **-** | **-** | **Paratype** |
| *Imperator rhodopurpureus* | B19 (IS32) | Czechia: Central Bohemian region | LT223151 | - | - | - | **-** |
| *Imperator rhodopurpureus* | BRNM 766829 (B20, IS379) | Czechia: South Moravian region | LT223152 | - | - | - | **-** |
| *Imperator rhodopurpureus* | FR2011123 | France: Occitanie | MH011866 | - | - | - | **-** |
| *Imperator rhodopurpureus* | FR2015605 | France: Auvergne-Rhône-Alpes | MH011868 | - | - | - | **-** |
| *Imperator rhodopurpureus* | FR2015611 | France: Auvergne-Rhône-Alpes | MH011869 | - | - | - | **-** |
| *Imperator rhodopurpureus* | MB241006/01 | Italy: Tuscany | PQ569372 | - | - | - | **-** |
| *Imperator rhodopurpureus* | UF503 | Portugal | HM347664 | - | - | - | **-** |
| *Imperator rhodopurpureus* | JAM0639 | Spain | HM347647 | - | - | - | **-** |
| *Imperator rhodopurpureus* | F-1030582 | Sweden: Östergötland co. | ON000903 | - | - | - | **-** |
| *Imperator rhodopurpureus* | FR2015613 | Switzerland: Geneva canton | MH011870 | - | - | - | **-** |
| *Imperator rhodopurpureus* | AT1996058 | United Kingdom: England | UDB000654 | - | - | - | **-** |
| ***Imperator rhodopurpureus* f. *rhodopurpureus*** | **CNF 10/322** | **Croatia: City of Zagreb** | **QB013786** | **-** | **-** | **-** | **-** |
| ***Imperator rhodopurpureus* f. *xanthopurpureus*** | **CNF 10/53** | **Croatia: Varaždin co.** | **QB013787** | **-** | **-** | **-** | **-** |
| ***Imperator rhodopurpureus* f. *xanthopurpureus*** | **CNF 10/135** | **Croatia: Varaždin co.** | **QB013788** | **-** | **-** | **-** | **-** |
| ***Imperator rhodopurpureus* f. *xanthopurpureus*** | **FR2020646** | **France: Bourgogne-Franche-Comté** | **QB013789** | **-** | **-** | **-** | **-** |
| ***Imperator rhodopurpureus* f. *xanthopurpureus*** | **FR2024494** | **France: Bourgogne-Franche-Comté** | **QB013790** | **-** | **-** | **-** | **-** |
| ***Imperator rhodopurpureus* f. *xanthopurpureus*** | **FR2024495** | **France: Île-de-France** | **QB013791** | **-** | **-** | **-** | **-** |
| ***Imperator rhodopurpureus* f. *xanthopurpureus*** | **MG1038** | **Italy: Friuli Venezia Giulia** | **QB013793** | **-** | **-** | **-** | **-** |
| ***Imperator rhodopurpureus* f. *xanthopurpureus*** | **MG1039** | **Italy: Friuli Venezia Giulia** | **QB013794** | **-** | **-** | **-** | **-** |
| ***Imperator rhodopurpureus* f. *xanthopurpureus*** | **LJF9368** | **Slovenia: Koper admin. unit** | **QB013792** | **PZ979658** | **-** | **-** | **-** |
| ***Imperator torosus*** | **SOMF30963** | **Bulgaria: Pazardzhik province** | **QB013811** | **-** | **-** | **-** | **-** |
| ***Imperator torosus*** | **SOMF30964** | **Bulgaria: Pazardzhik province** | **QB013812** | **-** | **-** | **-** | **-** |
| ***Imperator torosus*** | **CNF 10/249** | **Croatia: Primorje-Gorski Kotar co.** | **PZ973659** | | **PZ973365*** | **PZ973367*** | **Proposed conserved type** |
| ***Imperator torosus*** | **CNF 10/323** | **Croatia: Primorje-Gorski Kotar co.** | **QB013800** | **-** | **-** | **-** | **-** |
| ***Imperator torosus*** | **CNF 10/79** | **Croatia: Primorje-Gorski Kotar co.** | **QB013798** | **-** | **-** | **-** | **-** |
| ***Imperator torosus*** | **PRM965778** | **Czechia: Zlín region** | **QB013810** | **-** | **-** | **-** | **-** |
| ***Imperator torosus*** | **FR2017509** | **France: Auvergne-Rhône-Alpes** | **QB013801** | **-** | **-** | **-** | **-** |
| ***Imperator torosus*** | **GS-47** | **Germany: Bavaria** | **QB013802** | **-** | **-** | **-** | **-** |
| ***Imperator torosus*** | **JS 05/07** | **Germany: Bavaria** | **QB013797** | **-** | **-** | **-** | **-** |
| ***Imperator torosus*** | **JS 01/09** | **Germany: Rhineland-Palatinate** | **QB013796** | **-** | **-** | **-** | **-** |
| ***Imperator torosus*** | **KW-2023-08-27-1** | **Germany: Rhineland-Palatinate** | **QB013803** | **-** | **-** | **-** | **-** |
| ***Imperator torosus*** | **KW-2023-08-27-2** | **Germany: Rhineland-Palatinate** | **QB013804** | **-** | **-** | **-** | **-** |
| ***Imperator torosus*** | **KW-2023-08-27-3** | **Germany: Rhineland-Palatinate** | **QB013805** | **-** | **-** | **-** | **-** |
| ***Imperator torosus*** | **AL10/144** | **Hungary: Heves co.** | **QB013795** | **PZ979659** | **-** | **-** | **-** |
| ***Imperator torosus*** | **MG1020** | **Italy: Veneto** | **QB013807** | **-** | **-** | **-** | **-** |
| ***Imperator torosus*** | **MG1033 (FRJ009-2017)** | **Italy: Veneto** | **QB013808** | **-** | **-** | **-** | **-** |
| ***Imperator torosus*** | **MG958** | **Italy: Veneto** | **QB013806** | **-** | **-** | **-** | **-** |
| ***Imperator torosus*** | **PN-2025-07-22** | **Romania: Suceava co.** | **QB013809** | **-** | **-** | **-** | **-** |
| ***Imperator torosus*** | **CNF 10/320** | **Slovenia: Črnomelj admin. unit** | **QB013799** | **-** | **-** | **-** | **-** |
| ***Imperator torosus*** | **ZT Myc 0094431** | **Switzerland: Bern canton** | **QB013813** | **PZ979660** | **-** | **-** | **-** |
| ***Imperator torosus*** | **ZT Myc 0094438** | **Switzerland: Glarus canton** | **QB013817** | **PZ979663** | **-** | **-** | **-** |
| ***Imperator torosus*** | **ZT Myc 0094439** | **Switzerland: Neuchâtel canton** | **PZ973660** | | **-** | **-** | **-** |
| ***Imperator torosus*** | **ZT Myc 0094434** | **Switzerland: Schwyz canton** | **QB013815** | **-** | **-** | **-** | **-** |
| ***Imperator torosus*** | **ZT Myc 0094432** | **Switzerland: Zürich canton** | **QB013814** | **PZ979661** | **-** | **-** | **-** |
| ***Imperator torosus*** | **ZT Myc 0094435** | **Switzerland: Zürich canton** | **QB013816** | **PZ979662** | **-** | **-** | **-** |
| *Imperator torosus* | Btor1 | Germany: Bavaria | - | DQ534661 | - | - | **-** |
| *Imperator torosus* | MB000258 | Germany | MW520192 | MW521084 | MW566748* | MW560082* | **-** |
| *Imperator* sp. | WPM426 | China | - | - | PP060044* | - | **-** |
| *Rubroboletus sinicus* | HKAS68620 | China: Yunnan province | KJ951991, MW520195 | MW520195, KY418896 | - | - | **-** |
