# Supplementary material for: An integrated taxonomic approach refines the Imperator torosus species concept and reveals the cryptic I. occultus sp. nov. (Boletales, Boletaceae)
Source: MycoKeys. 2026 Nov 2;142:335–413. doi: 10.3897/mycokeys.142.208955 (PMC13749066; doi:10.3897/mycokeys.142.208955)
Supplement: Supplementary file 2 [file mycokeys-142-335-s002.docx]

**Supplementary Table S2.** SoilGrids v2.0 median (Q0.5) point predictions for pH in water (phh2o) and coarse-fragment content (cfvo) at the four targeted localities. Predictions are modelled at 250-m resolution; mapped integers were divided by 10 to give pH units and cm³/100 cm³ (vol.%), respectively.

| **Locality / country** | **Depth** | **pH(H₂O)** | **Coarse fragments (vol.%)** |
| --- | --- | --- | --- |
| Sauvabelin, Switzerland | 0–5 cm | 5.6 | 5.0 |
| Sauvabelin, Switzerland | 5–15 cm | 6.0 | 6.0 |
| Sauvabelin, Switzerland | 15–30 cm | 6.0 | 6.0 |
| Sauvabelin, Switzerland | 30–60 cm | 5.9 | 6.0 |
| Sauvabelin, Switzerland | 60–100 cm | 6.0 | 6.0 |
| Sauvabelin, Switzerland | 100–200 cm | 6.2 | 9.0 |
| Castelvecchio/Caldaro, Italy | 0–5 cm | 6.4 | 19.0 |
| Castelvecchio/Caldaro, Italy | 5–15 cm | 6.7 | 31.0 |
| Castelvecchio/Caldaro, Italy | 15–30 cm | 6.7 | 26.0 |
| Castelvecchio/Caldaro, Italy | 30–60 cm | 6.2 | 20.0 |
| Castelvecchio/Caldaro, Italy | 60–100 cm | 5.8 | 20.0 |
| Castelvecchio/Caldaro, Italy | 100–200 cm | 6.0 | 20.0 |
| Podlesje, Slovenia | 0–5 cm | 5.8 | 3.9 |
| Podlesje, Slovenia | 5–15 cm | 6.4 | 4.0 |
| Podlesje, Slovenia | 15–30 cm | 6.4 | 3.8 |
| Podlesje, Slovenia | 30–60 cm | 6.2 | 5.1 |
| Podlesje, Slovenia | 60–100 cm | 6.4 | 6.4 |
| Podlesje, Slovenia | 100–200 cm | 6.1 | 6.4 |
| Stari Trg ob Kolpi, Slovenia | 0–5 cm | 6.1 | 3.1 |
| Stari Trg ob Kolpi, Slovenia | 5–15 cm | 6.4 | 3.0 |
| Stari Trg ob Kolpi, Slovenia | 15–30 cm | 6.4 | 2.8 |
| Stari Trg ob Kolpi, Slovenia | 30–60 cm | 6.5 | 2.7 |
| Stari Trg ob Kolpi, Slovenia | 60–100 cm | 6.4 | 3.0 |
| Stari Trg ob Kolpi, Slovenia | 100–200 cm | 6.4 | 3.1 |

**Locality basis and voucher linkage.** Sauvabelin, Switzerland: the presumptive original-locality point published by Simonini et al. (2025) for Secretan’s historical find; Castelvecchio/Caldaro, Italy: the locality of MCVE18453, the epitype of *Boletus torosus*; Podlesje, Slovenia: voucher LJF 8893 (*Imperator occultus*); Stari Trg ob Kolpi, Slovenia: voucher CNF 10/320 (*Imperator torosus* s. str.).

Focal coordinates (WGS84): Sauvabelin, 46.531389° N, 6.642500° E; Castelvecchio, 46.382500° N, 11.238333° E; Podlesje, 45.503000° N, 14.992167° E; Stari Trg ob Kolpi, 45.515444° N, 15.078167° E.

Sources: Poggio et al. (2021), SoilGrids 2.0, doi:10.5194/soil-7-217-2021; ISRIC SoilGrids documentation and point-query service. Variables: phh2o and cfvo; Q0.5; d_factor = 10. Values were checked against the corresponding official SoilGrids Web Map Service layers.

**Supplementary Table S3.** Monthly and annual climatic context of historical Sauvabelin (1811–1847) and Monticolo/Montiggl (1977–2013), the observational proxy for the Castelvecchio epitype locality.

| **Month** | **CH Tmean (°C)** | **IT Tmean (°C)** | **CH Tmax (°C)** | **IT Tmax (°C)** | **CH P (mm)** | **IT P (mm)** |
| --- | --- | --- | --- | --- | --- | --- |
| January | -0.2 | 1.9 | 1.9 | 5.9 | 57.2 | 32.1 |
| February | 1.3 | 4.0 | 4.1 | 9.2 | 53.7 | 27.6 |
| March | 4.8 | 8.7 | 8.0 | 14.7 | 65.0 | 43.1 |
| April | 8.0 | 12.1 | 11.3 | 18.5 | 71.7 | 61.2 |
| May | 12.3 | 16.0 | 15.9 | 22.5 | 91.3 | 87.9 |
| June | 15.5 | 19.6 | 19.7 | 26.3 | 102.6 | 90.4 |
| July | 17.7 | 21.8 | 22.2 | 28.9 | 97.6 | 102.3 |
| August | 16.9 | 21.5 | 21.3 | 28.4 | 100.8 | 91.3 |
| September | 13.8 | 17.3 | 17.7 | 23.4 | 104.2 | 74.2 |
| October | 9.8 | 12.1 | 12.4 | 17.1 | 99.7 | 90.4 |
| November | 4.7 | 6.1 | 6.8 | 10.2 | 92.9 | 70.7 |
| December | 1.7 | 2.2 | 3.5 | 5.8 | 60.3 | 43.6 |
| Annual | 8.9 | 12.0 | 12.1 | 17.6 | 997.0 | 814.6 |

CH: reconstructed Sauvabelin climate, 1811–1847; IT: official Monticolo/Montiggl station data, 1977–2013 (89100MS; 530 m a.s.l.). Tmean denotes mean temperature, Tmax the mean of daily maximum temperatures, and P the mean monthly precipitation total. Annual P denotes mean annual precipitation, not the mean of monthly amounts.

Swiss values average 37 complete monthly or annual reconstructions for the grid cell LV95 E 2538500, N 1153500 (46.529968° N, 6.637037° E). Monticolo Tmean was recalculated by averaging all available published year-specific monthly and annual values for 1977–2013; Tmax and precipitation retain the provider’s published multiannual summaries. The available Italian temperature series have 34 years per month except June (33); annual temperature has 33 years. Precipitation covers all 37 years. No missing observations were imputed.

Monticolo Tmean follows the provider’s definition (mean daily minimum + mean daily maximum)/2. Values are rounded to one decimal place only after calculation; sums of rounded monthly precipitation means need not equal the annual mean. Annual Tmax is the mean of daily maximum temperatures over the year.

Sources: Swiss Tmean and precipitation, Imfeld et al. (2022, 2023): PANGAEA data, doi:10.1594/PANGAEA.950236; method, doi:10.5194/cp-19-703-2023. Swiss Tmax, Imfeld & Brönnimann (2025, 2026): BORIS data v1.1, doi:10.48620/87086; method, doi:10.5194/essd-18-4019-2026. Monticolo: Provincia Autonoma di Bolzano, Meteo Alto Adige, station 89100MS, 1977–2013: monthly temperature workbook; monthly and annual precipitation workbook.
